# Pharmacological Activation of cGAS for Cancer Immunotherapy

**DOI:** 10.3389/fimmu.2021.753472

**Published:** 2021-11-26

**Authors:** Kyle M. Garland, Jonah C. Rosch, Carcia S. Carson, Lihong Wang-Bishop, Ann Hanna, Sema Sevimli, Casey Van Kaer, Justin M. Balko, Manuel Ascano, John T. Wilson

**Affiliations:** ^1^ Department of Chemical and Biomolecular Engineering, Vanderbilt University, Nashville, TN, United States; ^2^ Department of Biomedical Engineering, Vanderbilt University, Nashville, TN, United States; ^3^ Department of Medicine, Vanderbilt University Medical Center, Nashville, TN, United States; ^4^ Department of Bioengineering, Northeastern University, Boston, MA, United States; ^5^ Vanderbilt-Ingram Cancer Center, Vanderbilt University Medical Center, Nashville, TN, United States; ^6^ Department of Biochemistry, Vanderbilt University Medical Center, Nashville, TN, United States; ^7^ Vanderbilt Institute for Infection, Immunology, and Inflammation, Vanderbilt University Medical Center, Nashville, TN, United States; ^8^ Vanderbilt Center for Immunobiology, Vanderbilt University Medical Center, Nashville, TN, United States; ^9^ Vanderbilt Institute of Chemical Biology, Vanderbilt University Medical Center, Nashville, TN, United States

**Keywords:** cancer, cGAS/STING pathway, endosomal escape, immunotherapy, innate immune agonist, intratumoral, nanoparticles, nucleic acid therapy

## Abstract

When compartmentally mislocalized within cells, nucleic acids can be exceptionally immunostimulatory and can even trigger the immune-mediated elimination of cancer. Specifically, the accumulation of double-stranded DNA in the cytosol can efficiently promote antitumor immunity by activating the cGAMP synthase (cGAS) / stimulator of interferon genes (STING) cellular signaling pathway. Targeting this cytosolic DNA sensing pathway with interferon stimulatory DNA (ISD) is therefore an attractive immunotherapeutic strategy for the treatment of cancer. However, the therapeutic activity of ISD is limited by several drug delivery barriers, including susceptibility to deoxyribonuclease degradation, poor cellular uptake, and inefficient cytosolic delivery. Here, we describe the development of a nucleic acid immunotherapeutic, NanoISD, which overcomes critical delivery barriers that limit the activity of ISD and thereby promotes antitumor immunity through the pharmacological activation of cGAS at the forefront of the STING pathway. NanoISD is a nanoparticle formulation that has been engineered to confer deoxyribonuclease resistance, enhance cellular uptake, and promote endosomal escape of ISD into the cytosol, resulting in potent activation of the STING pathway *via* cGAS. NanoISD mediates the local production of proinflammatory cytokines *via* STING signaling. Accordingly, the intratumoral administration of NanoISD induces the infiltration of natural killer cells and T lymphocytes into murine tumors. The therapeutic efficacy of NanoISD is demonstrated in preclinical tumor models by attenuated tumor growth, prolonged survival, and an improved response to immune checkpoint blockade therapy.

## Introduction

Nucleic acid sensing is a fundamental part of the innate immune system that can galvanize immune responses against pathogens and diseased cells (1). During cellular homeostasis, DNA is largely sequestered from the cytosol inside the nucleus and mitochondria (2). Accordingly, the abnormal accumulation of DNA inside the cytosol is indicative of cellular distress. The aberrant presence of such “danger signals” within the cytosol can trigger various pattern recognition receptors (PRRs) and lead to a myriad of immunological responses (3). Moreover, the physiochemical properties of cytosolic DNA (*e.g.* nucleotide sequence, base pair (BP) length, *etc.*) can drastically influence the nature of the resultant immune response by modulating PRR activation (4).

The stimulator of interferon genes (STING) cellular signaling pathway is a major DNA sensing pathway that bridges the gap between innate and adaptive immunity. The STING protein is located on the endoplasmic reticulum (5) and is directly activated by cyclic dinucleotides (CDNs) (6), such as the endogenous second messenger, 2′3′-cyclic guanosine monophosphate–adenosine monophosphate (cGAMP) (7). Molecules of cGAMP are produced intracellularly by cGAMP synthase (cGAS) when the enzyme detects double-stranded DNA (dsDNA) in the cytosol (7–10). Notably, the recognition of cytosolic dsDNA by cGAS is independent of nucleotide sequence (11), and therefore this DNA sensing pathway is broadly applicable to a vast number of microbial infections as well as the detection of self dsDNA leakage resulting from cellular malfunction, a common feature of many precancerous cells.

STING activation results in the local production of type-I interferons (IFN-I) and various other proinflammatory cytokines, the specific profile of which depends on cellular context as well as the type, intensity, and duration of the stimulant (12). This dynamic cytokine response generally creates an inflammatory microenvironment, which in certain settings, can promote robust cellular immune responses towards pathogens and diseases (13). Notably, localized STING signaling has been identified as critical for the spontaneous induction of antitumor immunity (14). Indeed, STING knockout (KO) mice (*i.e. Tmem173^–/–^*) exhibit defective tumor control in some murine tumor models and demonstrate a significantly reduced therapeutic response to immune checkpoint blockade (ICB) therapy relative to wildtype mice (14). Moreover, these preclinical findings have corresponded with clinical data from human cancer patients that has positively correlated cGAS/STING activation with the presence of tumor infiltrating T lymphocytes (*i.e.* T cells) (15) as well as T cell–inflamed tumors with increased overall survival (16) and responsiveness to ICB therapy (17, 18).

Under the proper conditions, STING signaling can mediate cancer cell death either directly (19, 20) or indirectly by supporting cytotoxic T lymphocyte (CTL) (21) and natural killer (NK) cell (22, 23) responses. Additionally, the STING pathway is iatrogenically activated by many of the classical cancer therapies (*e.g.* radiation, certain chemotherapies, *etc.*) and may contribute to enhanced therapeutic responses in such cases (24, 25). Indeed, in murine tumor models, antitumor immune responses generated by STING signaling are essential to achieving maximum therapeutic efficacy in response to radiotherapy (26). These discoveries have collectively motivated the development of synthetic STING pathway agonists for applications in cancer immunotherapy.

Numerous preclinical studies using synthetic STING agonists have now shown that targeted activation of the STING pathway within established murine tumors can shift the immune profile of an immunosuppressive tumor microenvironment (TME) toward an immunogenic state that is conducive to productive antitumor immunity and to enhancing the therapeutic efficacy of multiple immunotherapeutic modalities (21, 27, 28). Accordingly, many synthetic STING agonists are currently being explored as cancer therapeutics in human clinical trials (29, 30). However, it is worth noting that all of the STING pathway agonists currently in clinical development are direct activators of the STING protein or inhibit antagonists of the pathway (28). Compared to the STING protein, cGAS has been relatively underappreciated as a druggable target for cancer immunotherapy (31), despite the potential of a cGAS-targeting therapeutic to more closely mimic endogenous STING signaling by simulating natural, endogenous DNA sensing.

There are many drug delivery challenges that must be overcome to activate cGAS with interferon stimulatory DNA (ISD), which may explain why the development of cGAS agonists has been remarkably limited thus far. Efficient cytosolic delivery of ISD is critical to the pharmacological activation of cGAS, yet freely administered ISD experiences negligible cellular uptake and is quickly cleared and degraded (32). Furthermore, cGAS possesses several DNA-length dependencies that affect both the activation of the pathway (33) and the strength of STING signaling (*i.e.* the amount of STING-driven gene expression) (34). Here, we have engineered a nucleic acid immunotherapeutic, NanoISD, which can target cGAS and exploit the DNA sensing pathway in the context of local cancer immunotherapy *via* the cytosolic delivery of noncoding, immunostimulatory dsDNA.

**Figure 1:**
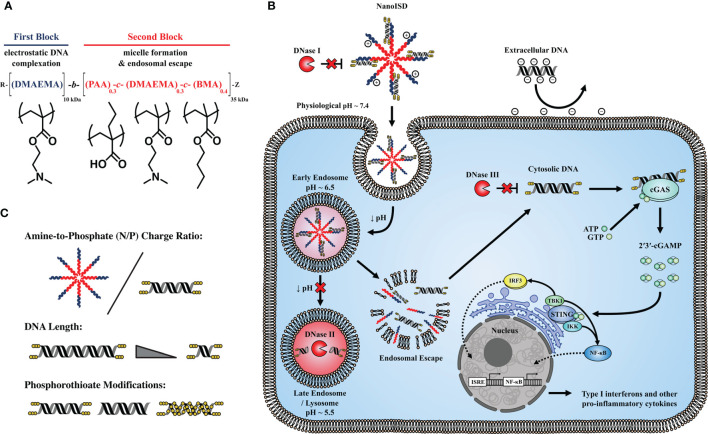
NanoISD – A nanoscale activator of the cGAS/STING pathway. NanoISD is fabricated *via* the self-assembly of an optimized interferon stimulatory DNA (ISD) sequence in complex with endosome-destabilizing polymer nanoparticles. **(A)** Chemical composition of poly[(DMAEMA)-*block*-(PAA-*co*-DMAEMA-*co*-BMA)] (D-PDB). **(B)** Schematic representation of NanoISD activating cytosolic cGAS by evading major deoxyribonuclease and mediating cellular uptake and endosomal escape. **(C)** Design variables explored for DNA/polymer complexes include N/P charge ratio, dsDNA length, and degree and location of phosphorothioate backbone modifications.

The well-established, endosomolytic polymer, poly[(DMAEMA)-*block*-(PAA-*co*-DMAEMA-*co*-BMA)] (D-PDB) (35–51) was used to electrostatically complex dsDNA into environmentally responsive nanoparticles capable of achieving cytosolic delivery. The DNA/polymer complexes were characterized using a library of synthetic ISD to study the effects of both N/P charge ratio (*i.e.* molar amount of protonated amines on the polymer corona / molar amount of phosphates on the nucleic acid backbone) and dsDNA composition on nanoparticle stability, transfection efficiency, cGAS activation, and antitumor immunity. *In vitro* screening of various DNA/nanoparticle complexes resulted in the identification of an optimized cGAS adjuvant, a phosphorothioate-capped 95-BP dsDNA/D-PDB complex, termed NanoISD. NanoISD is a nanoparticle formulation that confers deoxyribonuclease resistance, cellular uptake, endosomal escape, and potent activation of the STING pathway *via* cGAS (**Figure 1**). Notably, the direct injection of NanoISD into murine tumors triggers the production of proinflammatory cytokines, which leads to the tumor infiltration of both NK cells and T lymphocytes. Finally, the therapeutic efficacy of NanoISD is demonstrated in preclinical tumor models by attenuated tumor growth, increased survival, and an improved therapeutic response to ICB therapy.

## Results and Discussion

### Engineering DNA/Polymer Nanoparticles for Intracellular Activation of cGAS

A library of synthetic ISD was created with a distinct set of design principles intended to yield structurally optimized cGAS ligands (**Supplementary Figure 1**). The library contains 4 dsDNA sequences of different lengths (*i.e.* 20-BP, 45-BP, 70-BP, and 95-BP dsDNA). To the extent possible, based on the designated dsDNA length, the individual ISD strands comprise poly(AC) and poly(AAC) repeats, which are each 20 nucleotides in length and are interspersed with random sequence spacers that are each 5 nucleotides in length. This unique composition of the ISD sequences should provide enough footing to minimize strand slippage. Additionally, the individual ISD strands exhibit positive free energies for secondary structure formation and are therefore not disposed to hairpins and self-dimerization. Moreover, the ISD has melting temperatures that are sufficiently high to maintain double-stranded morphologies at biologically relevant temperatures (*i.e.* 37°C). Lastly, the synthetic ISD sequence contains three terminal phosphorothioate bonds (*i.e.* “caps”) on both ends of each complementary DNA strand to inhibit exonuclease degradation, a known feature of such modifications (52).

To overcome the delivery barriers that limit the activity of ISD, we employed a diblock copolymer, D-PDB, which has previously been used primarily for the cytosolic delivery of small-interfering RNA (siRNA) (35–51). Under a physiological pH of ~ 7.4, D-PDB self-assembles into colloidally stable, nanoparticle micelles with a cationic corona that can electrostatically load nucleic acids. In response to the decrease in endosomal pH that follows cellular uptake, these nanoparticles disassemble. The hydrophobic moieties of the polymer become accessible and then disrupt the endosomal membrane, whereupon the exogenous nucleic acid cargo escapes from the endosome into the cytosol of the cell. While nuclear localization is required for most applications of intracellular DNA delivery (*e.g.* gene therapy), DNA delivery to the cytosol is adequate and perhaps better for pharmacologically targeting cGAS, since the PRR is primarily activated by DNA within the cytosol (8). Thus, in terms of maximizing cGAS activation, D-PDB has potential to be advantageous relative to nanocarriers that are designed to deliver their nucleic acid cargo to the nucleus of cells.

**Figure 2:**
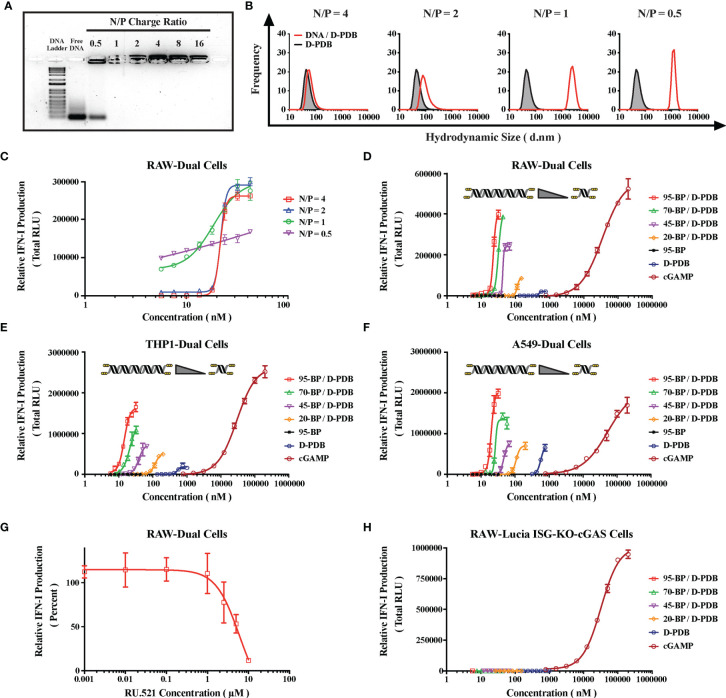
Engineering DNA/Polymer Nanoparticles for Intracellular Activation of cGAS. **(A)** Agarose gel image. DNA Ladder refers to the TrackIt™ 1 Kb Plus DNA Ladder, and Free DNA refers to uncomplexed phosphorothioate-capped 95-BP dsDNA. Lanes comprise 1 µg DNA mixed with the indicated amount of D-PDB. **(B)** DLS analysis of phosphorothioate-capped 95-BP dsDNA/D-PDB complexes at varying N/P charge ratios. Frequency indicates the number-based particle size distribution. Hydrodynamic size indicates the particle diameter in nm. **(C)** RAW-Dual reporter cell assay of phosphorothioate-capped 95-BP dsDNA/D-PDB complexes at varying N/P charge ratios. **(D)** RAW-Dual reporter cell assay of synthetic, variable-length ISD library complexed to D-PDB at an N/P charge ratio of 4, and indicated experimental controls were used. **(E)** THP1-Dual reporter cell assay of synthetic, variable-length ISD library complexed to D-PDB at an N/P charge ratio of 4, and indicated experimental controls were used. **(F)** A549-Dual reporter cell assay of synthetic, variable-length ISD library complexed to D-PDB at an N/P charge ratio of 4, and indicated experimental controls were used. **(G)** Dose response of the cGAS inhibitor, RU.521 in RAW-Dual reporter cells. After a 4 hour incubation with RU.521, cells were treated with 25 nM phosphorothioate-capped 95-BP dsDNA complexed to D-PDB at an N/P charge ratio of 4. **(H)** RAW-Lucia ISG-KO-cGAS reporter cell assay of synthetic, variable-length ISD library complexed to D-PDB at an N/P charge ratio of 4, and indicated experimental controls were used. The dose response curves for free D-PDB are positioned along the x-axis in terms of the molar amount of polymer chains rather than molar amount of loaded dsDNA, and each dose response that utilized the polymer was administered using equivalent D-PDB concentrations.

To determine an ideal N/P charge ratio (*i.e.* molar amount of protonated amines on the polymer corona / molar amount of phosphates on the nucleic acid backbone) for the ISD and polymer, polymeric micelles of D-PDB were complexed with varying concentrations of phosphorothioate-capped 95-BP dsDNA, one of the ISD molecules from the starting library. The resultant complexes were then analyzed *in vitro via* agarose gel electrophoresis, dynamic light scattering (DLS), and reporter cell assays for IFN-I production (**Figure 2**).

Agarose gel electrophoresis was run to determine the N/P charge ratio at which complete complexation is achieved (**Figure 2A**). Consistent with previous findings for D-PBD with shorter double-stranded RNA molecules (35, 50), it was determined that N/P charge ratios of 1 and greater enabled complete loading of the phosphorothioate-capped 95-BP dsDNA. Conversely, an N/P charge ratio of 0.5 exhibited incomplete complexation, as demonstrated by the migration of unbound DNA, which formed a band corresponding to that of the free DNA.

DLS was subsequently performed to characterize the size and polydispersity of the complexes (**Figure 2B**). DLS analysis demonstrated that uncomplexed D-PDB micelles are ~ 45-60 nm in diameter and that loading phosphorothioate-capped 95-BP dsDNA at an N/P charge ratio of 4 results in slightly larger nanoparticles that are ~ 60-90 nm in diameter. As the N/P charge ratio was lowered, the measured hydrodynamic size significantly increased to micrometer diameters that are indicative of particle aggregation. Notably, larger particles (*i.e.* greater than 100 nm) are not ideal for *in vivo* cancer applications, since particle permeability and distribution within tumors are known to decrease with increasing particle size (53).

To determine the *in vitro* activity of the complexes, a reporter cell assay for cellular IFN-I production was utilized (**Figure 2C**). The reporter cells stably express a secreted luciferase downstream of interferon-stimulated response elements, and therefore luminescence can be used to track relative IFN-I production. RAW-Dual murine macrophages were treated with phosphorothioate-capped 95-BP dsDNA/D-PDB complexes that were formulated at different N/P charge ratios. Supernatants were collected 24 hours after the cells were treated, and the relative IFN-I production was quantified *via* luminescence. Notably, immunostimulatory activity was detected from all of the complexes. A maximum efficacy of ~ 275,000 Relative Light Units (RLU) was consistent for N/P charge ratios of 4, 2, and 1. Alternatively, the maximum efficacy for the N/P charge ratio of 0.5 over the same concentration range was substantially lower at ~ 170,000 RLU, which is likely due to the incomplete loading of the DNA that was observed in the agarose gel assay. Additionally, half-maximal effective concentration (EC_50_) values were determined for each dose response curve to allow for the comparison of *in vitro* potency. The calculated EC_50_ values for the N/P charge ratios of 4, 2, 1, and 0.5 were 22 nM, 22 nM, 15 nM, and 3 nM, respectively. Since *in vitro* potency is inversely related to EC_50_ values, the potency is greater for the N/P charge ratios of 1 and 0.5, both of which also exhibit larger sizes as determined by DLS. The apparent increase in potency accompanied by an increase in particle size is consistent with a recent report that larger, micrometer-sized polyplexes enhance *in vitro* transfection efficiency relative to compositionally-equivalent nanometer-sized polyplexes due to increased gravitational sedimentation (54). Interestingly, we characterized a second ISD library of relatively larger PCR-amplified dsDNA (**Supplementary Figure 2**) with D-PDB and found that the effects of N/P charge ratio on particle complexation, size, and activity were well conserved with dsDNA up to at least 5000-BP in length (**Supplementary Figure 3**). Based on these initial *in vitro* characterizations of the complexes, an N/P charge ratio of 4 was selected for all complexes used in the subsequent studies.

The degree of cGAS activation is directly proportional to the length of dsDNA recognized by cGAS (34, 55), yet larger molecular weight dsDNA can also compromise the colloidal stability of non-viral vectors (56) and thereby limit transfection efficiency. Moreover, there exist DNA-length thresholds for cGAS activation that are species-specific due to some small variations in the amino acid composition of the protein (33). For *in vitro* cell-based assays, a minimum dsDNA length of ~ 45-BP is required to activate human cGAS (hcGAS) (33), whereas dsDNA as low as ~ 20-BP in length can activate murine cGAS (mcGAS) (57, 58). Thus, the entire library of variable-length, synthetic ISD was evaluated, so that the molecular weight (*i.e.* BP length) of the ISD in complex with D-PDB micelles could be optimized.

DLS analysis of D-PDB and the synthetic ISD library revealed that while keeping the N/P charge ratio consistent at 4, particle size slightly increased as the BP length of the DNA increased (**Supplementary Figure 4**). This relationship was also observed for D-PDB complexed to the second ISD library of larger PCR-amplified dsDNA, though size appeared to plateau at ~ 140 nm in diameter once a dsDNA length of 1250-BP was reached (**Supplementary Figure 5**). For the N/P charge ratio of 4, colloidal stability of the complexes was lost when dsDNA length reached 10,000-BP, as evident from the complex’s nonuniform and highly polydisperse size range.

Reporter cell assays for IFN-I production were again utilized to evaluate *in vitro* activity of the complexes. RAW-Dual murine macrophages (**Figure 2D**), THP1-Dual human monocytes (**Figure 2E**), and A549-Dual adenocarcinomic human alveolar basal epithelial cells (**Figure 2F**) were all treated with each of the varied-length, synthetic ISD complexed to D-PDB over a range of ISD concentrations to generate dose response curves. The endogenous STING ligand, 2′3′-cGAMP was used as a positive control for IFN-I induction, and free D-PDB (*i.e.* not loaded with dsDNA) was used as a vehicle control. Additionally, free phosphorothioate-capped 95-BP dsDNA was used as a negative control to demonstrate the importance of the polymeric drug delivery vehicle. Maximum efficacy and EC_50_ values for each of the treatments can be found in the supplementary information (**Supplementary Figure 6**). Consistent with previous observations that cGAS is activated in a dsDNA length dependent manner (34), both the potency and efficacy of the complexes generally increased with increasing BP length of the dsDNA cargo in all three reporter cell lines. Interestingly, free D-PDB demonstrated a small but significant dose response, suggesting that the polymer has an intrinsic capacity for stimulating some degree of IFN-I production.

In accordance with the established dsDNA length thresholds for species-specific cGAS activation, the phosphorothioate-capped 20-BP dsDNA complexed to D-PDB (*i.e.* 20-BP/D-PDB) enhanced maximum efficacy relative to that of free D-PDB in the murine RAW-Dual reporter cells (*i.e.* ~ 85,000 *vs.* ~ 20,000 RLU, respectively) and did not affect baseline efficacy in the human A549-Dual reporter cells (*i.e.* both treatments ~ 70,000 RLU). However, in the human THP1-Dual reporter cells, the 20-BP/D-PDB treatment did slightly outperform free D-PDB in terms of maximum efficacy (*i.e.* ~ 50,000 RLU *vs.* ~ 20,000 RLU, respectively), despite the 20-BP dsDNA being shorter than the empirically established threshold for human cGAS activation (*i.e.* ~ 45-BP) (33). This subtle discrepancy may be due to cell line–specific phenomenon coupled with the phosphorothioate modifications of the ISD, as the threshold established in previous reports was determined using unmodified dsDNA (59, 60).

The role of cGAS in the immunostimulatory activity of the compounds was investigated in the RAW-Dual reporter cells by pretreating the cells with a dose response of the established small molecule inhibitor of cGAS, RU.521 (33, 61, 62) (**Figure 2G**). Four hours after incubation with RU.521, the cells were treated with the EC75 concentration of 95-BP/D-PDB (*i.e.* 25 nM), a treatment known to be consistently active. Analysis of the supernatant 24 hours after treatment revealed that the cGAS-specific inhibitor was able to significantly diminish the IFN-I signal at the higher concentrations, suggesting that the observed activity of the DNA/polymer complexes is indeed cGAS-dependent. Notably, RU.521 exhibited a half-maximal inhibitory concentration (IC_50_) value of ~ 5 µM.

To further explore the dependence of cGAS on the activity of the treatments, RAW-Lucia ISG-KO-cGAS reporter cells, which do not express cGAS, were treated with each of the varied-length, synthetic ISD complexed to D-PDB (**Figure 2H**). Free D-PDB and cGAMP were again used as controls for the experiment. While cGAMP, which activates STING downstream of cGAS, retained its IFN-I activity, no activity was detected from DNA/polymer complexes, suggesting that the activity from those treatments observed in the wildtype reporter cells were largely, if not entirely, cGAS-dependent. These findings also suggest that if alternative IFN-inducing DNA sensors, such as IFI204 (*e.g.* the murine ortholog of IFI16), are involved in the response to the DNA/polymer complexes, they must operate as dependent cofactors of cGAS. Interestingly, the activity of free D-PDB was also completely abolished in the RAW-Lucia ISG-KO-cGAS reporter cells. While D-PDB is unlikely to be a direct cGAS ligand, D-PDB may indirectly activate cGAS in the wildtype reporter cells by inducing the cytosolic accumulation of mitochondrial DNA. Indeed, cationic nanocarriers have been linked to toll-like receptor 9 (TLR9) (*i.e.* a PRR for unmethylated DNA rich in CpG motifs) and STING activation *via* their intrinsic capacity for mitochondrial damage and the subsequent release of mitochondrial DNA (63, 64).

Similar cGAS-dependent activity in the RAW-Dual reporter cells was also demonstrated for the larger PCR-amplified dsDNA library complexed to D-PDB (**Supplementary Figure 7**). The DNA length–dependent trends were conserved for the larger PCR-amplified dsDNA library in the wildtype reporter cells, though the maximum efficacy of the DNA/polymer complexes did saturate at ~ 615,000 RLU when a dsDNA length of 625-BP was reached. Additionally, the colloidally unstable 10,000-BP/D-PDB complexes exhibited a reduced maximum efficacy of ~ 470,000 RLU over the same concentration range, which could be attributed to its extensive polydispersity of size. Furthermore, the synthetic phosphorothioate-capped 95-BP dsDNA complexed to D-PDB, which had a maximum efficacy of ~ 1,000,000 RLU, drastically outperformed all of the PCR-amplified dsDNA complexed to D-PDB in terms of maximum efficacy, which is likely a consequence of its exonuclease resistance and highlights the importance of such modifications for enhancing cGAS activation.

The starting ISD library used for the experiments in **Figure 2** comprised synthetic dsDNA molecules that were produced *via* solid-phase phosphoramidite-based synthesis, which can accommodate routine, scalable production of dsDNA up to ~ 95-BP in length as well as the molecular modification of dsDNA (65, 66). Conversely, PCR-mediated amplification of dsDNA utilizes polymerase-based synthesis that does not allow for site-specific DNA modification outside of the primer sequence, and therefore PCR-mediated amplification of dsDNA is not readily amenable to phosphorothioate-capping. Accordingly, the synthetic, phosphorothioate-capped 95-BP dsDNA became the lead cGAS ligand. Thus, the nanoparticle complex of D-PDB and the phosphorothioate-capped 95-BP dsDNA at an N/P charge ratio of 4, herein referred to as NanoISD, was employed as a potent cGAS adjuvant for the subsequent studies investigating its utility in cancer immunotherapy.

### NanoISD Exhibits Deoxyribonuclease Resistance

**Figure 3:**
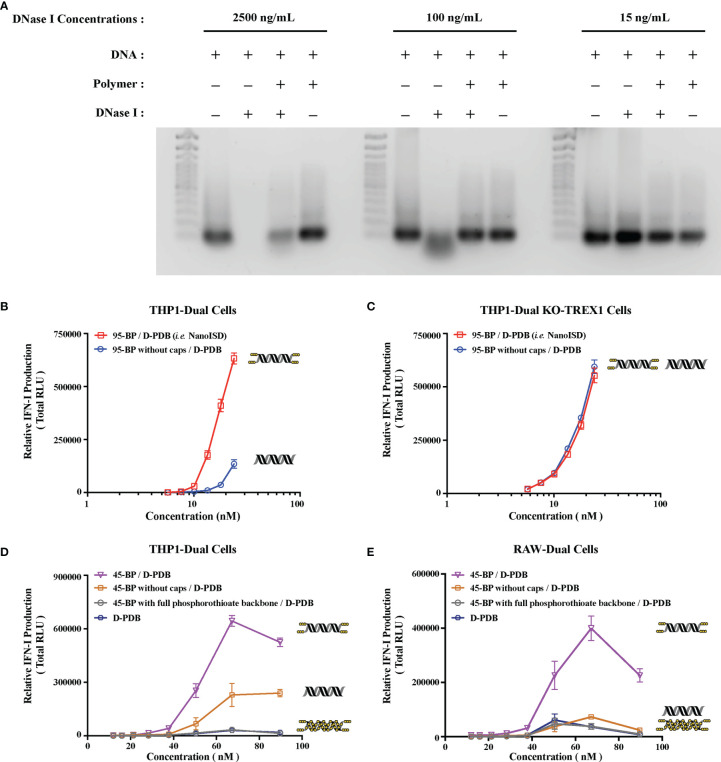
NanoISD Exhibits Deoxyribonuclease Resistance. **(A)** Agarose gel image. Lanes are as indicated. The TrackIt™ 100 bp DNA Ladder was used for reference. The DNA used in these studies was the phosphorothioate-capped 95-BP dsDNA at a concentration of 1 µg DNA/lane, and the polymer used was D-PDB at an N/P charge ratio of 4. **(B)** THP1-Dual reporter cell assay of 95-BP dsDNA with and without phosphorothioate caps complexed to D-PDB at an N/P charge ratio of 4. **(C)** THP1-Dual KO-TREX1 reporter cell assay of 95-BP dsDNA with and without phosphorothioate caps complexed to D-PDB at an N/P charge ratio of 4. **(D)** THP1-Dual reporter cell assay of synthetic 45-BP dsDNA complexed to D-PDB at an N/P charge ratio of 4, and D-PDB was used as an experimental control. Each 45-BP/D-PDB treatment comprised DNA with varying levels of phosphorothioate incorporation as indicated. **(E)** RAW-Dual reporter cell assay of synthetic 45-BP dsDNA complexed to D-PDB at an N/P charge ratio of 4, and D-PDB was used as an experimental control. Each 45-BP/D-PDB treatment comprised DNA with varying levels of phosphorothioate incorporation as indicated. The dose response curves for free D-PDB are positioned along the x-axis corresponding to their equivalent dsDNA-loaded treatments, as each dose response that utilized the polymer was administered using equivalent D-PDB concentrations.

Mammalian cells constitutively express many deoxyribonucleases (DNases) to prevent the potentially inflammatory accumulation of DNA outside of protective organelles. Notably, DNA present in systemic circulation, lysosomes, and cytosols is degraded by DNase I, DNase II (*i.e.* Acid DNase), and DNase III (*i.e.* TREX1), respectively (67–70). The inhibition of such nucleases can allow immunostimulatory dsDNA to remain intact for an extended period of time during delivery, which can lead to improved functionality. Notably, the length of cytosolic dsDNA directly influences the rate and extent of cGAS activation and thereby the amount of cGAMP produced (34). Thus, when dsDNA strands are not rapidly broken down into smaller fragments, they can exploit the length-dependence of the protein to promote maximal STING signaling. As the stability of DNA is essential for cGAS activation, the deoxyribonuclease resistance of NanoISD was evaluated (**Figure 3**).

Both free phosphorothioate-capped 95-BP dsDNA and NanoISD were incubated with three different concentrations of the endonuclease, DNase I (**Figure 3A**). 15 ng/mL was selected as it is the physiological level of DNase I in human serum (71), 100 ng/mL was selected as it is the concentration of recombinant human DNase I that can mediate the effective removal of DNA from blood circulation (72), and 2500 ng/mL was selected as an extreme high-dose control. Following incubation with DNase I, samples were heat-inactivated, and SDS was added to break apart the complexes. The samples were then run on a gel along with free phosphorothioate-capped 95-BP dsDNA and NanoISD that were not exposed to DNase I. While free phosphorothioate-capped 95-BP dsDNA was susceptible to degradation by the higher concentrations of DNase I, NanoISD exhibited marked protection of its DNA cargo from deoxyribonuclease degradation, which is likely due to polymer-mediated steric hindrance of the nuclease (*i.e.* nanoparticle packaging).

Since cGAS activation is greatly dependent on the length, concentration, and persistence of dsDNA in the cytosol, a particularly important negative regulator of the STING pathway is the exonuclease, TREX1 (*i.e.* DNase III). Indeed, it was recently discovered that DNA oxidized by reactive oxygen species (ROS) can significantly impede the exonuclease activity of TREX1, and such TREX1 inhibition was found to significantly potentiate STING signaling (73). Accordingly, the inhibition of TREX1 has recently been proposed as an immunotherapeutic strategy for the treatment of cancer (74).

The phosphorothioate caps of the synthetic ISD were implemented to boost immunostimulatory activity by obstructing the TREX1-mediated degradation of dsDNA that limits STING pathway activation. To further test the deoxyribonuclease resistance of the chemically modified ISD, reporter cell assays for IFN-I production were once again utilized. Phosphorothioate-capped 95-BP dsDNA and 95-BP dsDNA without caps were complexed with D-PDB micelles and incubated with THP1-Dual cells (**Figure 3B**) and THP1-Dual KO-TREX1 cells (**Figure 3C**).

In the wildtype reporter cells, the efficacy and potency of NanoISD were both significantly increased relative to D-PDB loaded with 95-BP dsDNA without phosphorothioate caps. As the caps inhibit TREX1 activity, it is likely that they enable a prolonged presence of the dsDNA in the cytosol and thereby enhance cGAS activation. This theory is supported by the finding that phosphorothioate caps on a 45-BP dsDNA also enhanced activity relative to 45-BP dsDNA without caps when delivered with D-PDB micelles to wildtype reporter cells (**Figures 3D, E**). Notably, it was also demonstrated that complete phosphorothioate modification of the dsDNA backbone rendered 45-BP dsDNA inactive, which is consistent with previous observations that phosphodiester bonds on dsDNA are required for cGAS activation (59, 75). One possible future opportunity for further enhancing the efficacy and potency of the ISD might involve incorporating intermittent phosphorothioate modifications along the DNA strands, which could potentially improve the deoxyribonuclease resistance and stability of the DNA while also maintaining a capacity for cGAS oligomerization/activation. The distance between each modification would likely need to be optimized to avoid deleterious effects on cGAS activation.

Moreover, in the TREX1 (*i.e.* DNase III) KO reporter cells, the efficacy and potency of the nanoparticles loaded with dsDNA lacking phosphorothioate caps were insignificantly different from that of NanoISD (**Figure 3C**), suggesting that in the wildtype reporter cells, TREX1 is mainly responsible for the reduced *in vitro* activity of the nanoparticles carrying unprotected dsDNA. Thus, in addition to the deoxyribonuclease resistance afforded by nanoparticle packaging, deoxyribonuclease activity was found to be further inhibited through the chemical modification of the synthetic dsDNA.

Notably, the IFN-I activity of the synthetic ISD library in the THP1-Dual reporter cells is entirely lost when delivered with the non-endosomolytic polymer, poly[(DMAEMA)-*block*-(BMA)] (D-B) at a consistent DNA concentration and N/P charge ratio (**Supplementary Figure 8**). D-B forms micelles that do not disassemble at low pH, and accordingly the polymer does not facilitate the cytosolic delivery of nucleic acid (50), which is necessary for cGAS activation. Conversely, D-PDB mediates endosomal escape at the onset of endosomal acidification due to the composition of the polymer (35) and the resultant loss of particle morphology under minimally acidic conditions (*e.g.* pH ~ 6.5), which leads to endosomal membrane disruption (50). Therefore, the dsDNA cargo loaded on D-PDB is likely released into the cytosol before endosomes can fully acidify. Since DNase II is mostly active under highly acidic conditions (*e.g.* pH ~ 5.5) (76), it is probable that the enzyme has a reduced opportunity to degrade the ISD when delivered with D-PDB. Indeed, the observed cGAS activation from NanoISD treatment is evidence that the dsDNA ligands are not appreciably degraded by DNase II in lysosomes. Thus, the chemical and physical composition of NanoISD as well as its intrinsic delivery route protect its cGAS ligand from three major deoxyribonucleases and thereby constitute NanoISD as an exceptionally potent cGAS adjuvant.

### NanoISD Enhances Cellular Uptake and Immunostimulatory Activity of ISD *In Vitro*

**Figure 4:**
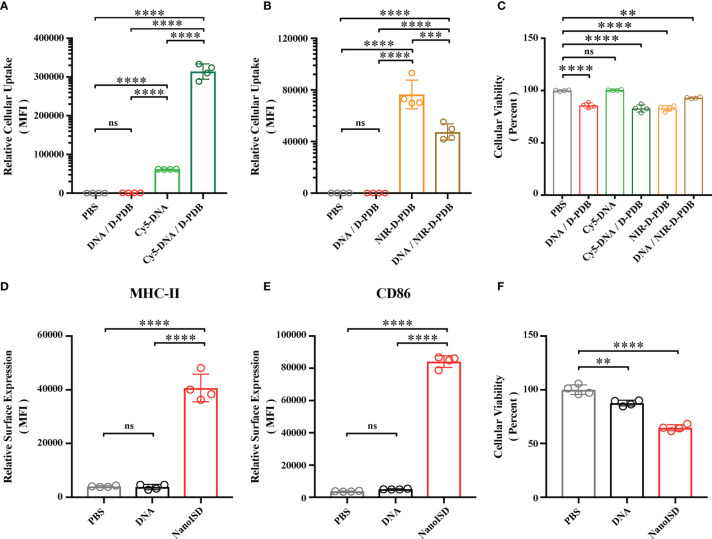
NanoISD Enhances Cellular Uptake and Immunostimulatory Activity of ISD *In Vitro*. **(A)** Flow cytometry analysis on the cellular uptake of 45 nM DNA (*i.e.* Cy5-labeled phosphorothioate-capped 95-BP dsDNA). Flow cytometry was conducted 4 hours after indicated treatment. The median fluorescence intensity (MFI) of Cy5-labeled DNA was quantified. **(B)** Flow cytometry analysis on the cellular uptake of 1.1 µM D-PDB (*i.e.* NIR-D-PDB), which corresponds to 45 nM DNA for a N/P charge ratio of 4. Flow cytometry was conducted 4 hours after indicated treatment. The MFI of NIR-664-labeled D-PDB was quantified. **(C)** Cellular viability determined 4 hours after indicated treatment as assessed by DAPI staining. Percent viable is relative to cells treated with PBS. **(D)** Flow cytometry analysis of the BMDC maturation marker, MHC-II conducted 24 hours after treatment of either PBS, 45 nM DNA (*i.e.* phosphorothioate-capped 95-BP dsDNA), or NanoISD at a dose corresponding to 45 nM. The MFI of anti-MHC-II-APC-Cy7 was quantified. **(E)** Flow cytometry analysis of the BMDC maturation marker, CD86 conducted 24 hours after treatment of either PBS, 45 nM DNA (*i.e.* phosphorothioate-capped 95-BP dsDNA), or NanoISD at a dose corresponding to 45 nM. The MFI of anti-CD86-PE-Cy7 was quantified. **(F)** Cellular viability determined 24 hours after indicated treatment as assessed by DAPI staining. Percent viable is relative to cells treated with PBS. A one-way ANOVA with Tukey test was used for statistical analysis. ****p < 0.0001, ***p < 0.005, **p < 0.01. ns, not significant.

DNA by itself does not readily pass through the negatively-charged plasma membrane of cells due to the relatively large, negatively-charged, and hydrophilic nature of DNA (77). However, when ISD is complexed at an N/P charge ratio of 4 with D-PDB micelles that exhibit a positive surface charge of +16.27 mV, the resultant DNA-loaded nanoparticles also exhibit a positive surface charge (**Supplementary Figure 9**) and can be efficiently endocytosed by DC2.4 dendritic cells *in vitro* as determined by flow cytometry analysis of fluorescently-labeled phosphorothioate-capped 95-BP dsDNA (*i.e.* Cy5-DNA) (**Figure 4A**). It is likely that the overall positive surface charge of NanoISD (*i.e.* +14.87 mV) afforded by D-PDB drives the cellular uptake of the nanoparticles, especially since free fluorescently-labeled D-PDB (*i.e.* NIR-D-PDB) is also efficiently endocytosed (**Figure 4B**). The positive charge of NanoISD does however dictate that the therapeutic be administered locally, as positively charged nanoparticles are typically poorly tolerated when administered systemically (78). There are many advantages to using local administration, especially for the delivery of a cancer immunotherapeutic (79). Indeed, while the direct injection of many classical cancer therapeutics (*e.g.* various chemotherapies) into solid tumors often results in therapeutic responses that are limited to the treated tumors, the local administration of a cancer immunotherapeutic can generate a systemic immune response with potential to clear untreated metastatic tumors (*i.e.* abscopal effect). Additionally, D-PDB treatment also confers a minor but significant degree of toxicity relative to cells treated with phosphate buffered saline (PBS) (**Figure 4C**). Notably, some toxicity may actually be beneficial in the context of killing cancer cells following local administration (80) and releasing tumor antigens, which can then be processed by APCs to promote the cancer immunity cycle (81).

The activation of APCs is a key feature of many innate immune agonists and is essential for cancer immunotherapies that are aimed at promoting antitumor T cells (82). Since STING pathway activation has been linked to APC maturation and T cell activation (83, 84), NanoISD was evaluated for its ability to promote APC maturation. Murine bone marrow-derived dendritic cells (BMDCs) were treated with either PBS, DNA (*i.e.* phosphorothioate-capped 95-BP dsDNA), or NanoISD. Markers of BMDC maturation (*i.e.* cell surface expression of CD86 and MHC-II) were quantified *via* flow cytometry 24 hours post treatment. It was determined that NanoISD evokes significantly enhanced maturation *in vitro* as compared to PBS-treated BMDCs and DNA-treated BMDCs (**Figures 4D, E**). Additionally, viability of the BMDCs after NanoISD treatment was comparable to that of the DC2.4 cells treated with the same concentration of NanoISD (**Figure 4F**).

### NanoISD Enhances Delivery and Immunostimulatory Activity of ISD *In Vivo*

**Figure 5:**
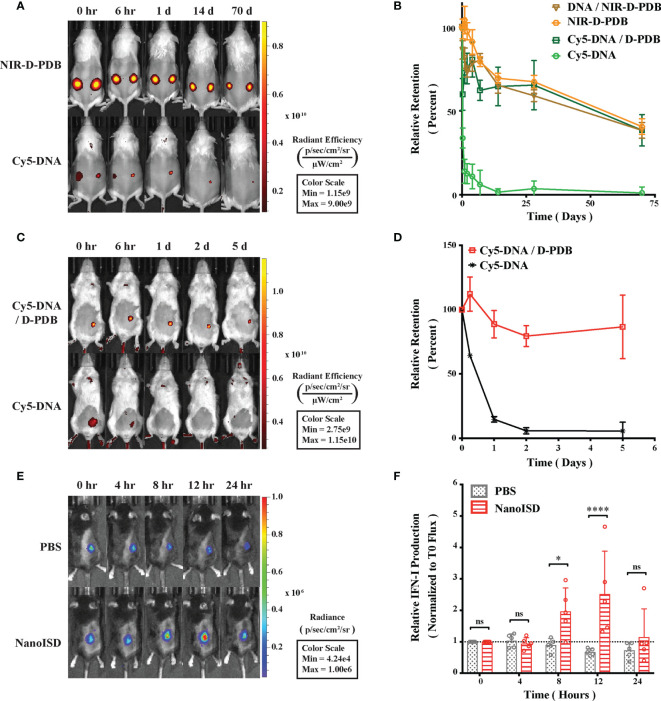
NanoISD Enhances Delivery and Immunostimulatory Activity of ISD *In Vivo*. **(A)** Representative fluorescence IVIS images evaluating the subcutaneous retention of NanoISD in CD-1 mice. D-PDB labeled with NIR-664-iodoacetamide (*i.e.* NIR-D-PDB) was used to track the polymer, and phosphorothioate-capped 95-BP dsDNA labeled with Cy5 (*i.e.* Cy5-DNA) was used to track the DNA. On the left flank of each mouse, individual uncomplexed agents were administered, and on the right flank of each mouse, complexes at an N/P charge ratio of 4 with the indicated fluorescent agent were administered. A subcutaneous injection was given as a single 100 µL dose of 2 µg DNA and/or 36 µg of polymer. **(B)** Retention profiles of NIR-D-PDB and Cy5-DNA either uncomplexed or complexed with unlabeled counterparts following subcutaneous administration in CD-1 mice. **(C)** Representative fluorescence IVIS images evaluating the tumor retention of NanoISD in BALB/cJ mice bearing orthotopic 4T1 tumors. Phosphorothioate-capped 95-BP dsDNA labeled with Cy5 (*i.e.* Cy5-DNA) was used to track the DNA. Cy5-DNA was administered by itself or in complex with D-PDB at an N/P charge ratio of 4. An intratumoral injection was given as a single 100 µL dose of 2 µg DNA. **(D)** Retention profiles of Cy5-DNA complexed to D-PDB and free Cy5-labeled DNA following intratumoral administration into orthotopic 4T1 breast tumors growing in BALB/c mice. **(E)** Representative luminescence IVIS images evaluating tumor IFN activity in C57BL/6J mice bearing B16.F10 IFN-LUC tumors. An intratumoral injection was given as a single 100 µL dose of either PBS or NanoISD at a dose corresponding to 2 µg DNA. **(F)** Longitudinal analysis of IFN activity following treatment. A two-way ANOVA with Sidak test was used for statistical analysis. ****p < 0.0001, *p < 0.05. ns, not significant.

By packaging dsDNA into cationic nanoparticles, it was hypothesized that NanoISD would address the rapid clearance of dsDNA by promoting local cellular uptake at the site of injection. To evaluate this, NanoISD and free ISD were injected subcutaneously into mice and the *in vivo* retention was evaluated *via* IVIS imaging using both fluorescently-labeled phosphorothioate-capped 95-BP dsDNA (*i.e.* Cy5-DNA) and fluorescently-labeled D-PDB (*i.e.* NIR-D-PDB) (**Figures 5A, B**). As anticipated, the free ISD was rapidly cleared from the injection site (*i.e.* half-life < 6 hours). Interestingly, D-PDB was retained at the injection site for an extended timeframe (*i.e.* half-life ~ 50 days) and also dramatically enhanced the retention of the dsDNA (*i.e.* half-life ~ 50 days).

The intratumoral retention of the fluorescently-labeled phosphorothioate-capped 95-BP dsDNA (*i.e.* Cy5-DNA) with and without the polymeric carrier (*i.e.* D-PDB) was then investigated using a murine orthotopic tumor model of 4T1 breast cancer (**Figures 5C, D**). Consistent with the subcutaneous retention data, the free ISD dispersed quickly (*i.e.* half-life ~ 12 hours), and the ISD complexed to the polymer (*i.e.* NanoISD) exhibited sustained retention (*i.e.* half-life > 5 days). The matching pharmacokinetic clearance profiles of free D-PDB and the ISD complexed to D-PDB is consistent with prolonged *in vivo* association of the two species. Additionally, this finding is disparate with previous data that has consistently reported a short retention profile (*e.g.* half-life < 1 day) for siRNA complexed to the same polymer (42, 47, 48, 51). This discrepancy is likely attributable to the higher valency of the polymer interaction with the significantly larger dsDNA cargo and/or the extra deoxyribonuclease resistance afforded by the phosphorothioate caps of the dsDNA. Notably, the local delivery of many innate immune agonists (*e.g.* CpG DNA, CDN STING agonists, *etc.*) results in widespread dissemination that can cause systemic inflammation and contribute to relatively low dose-limiting toxicities (85–87), while the enhanced local retention of NanoISD inherently limits the escape of nanoparticles into systemic circulation and therefore reduces the potential for systemic toxicity.

B16.F10 murine melanoma cells, which had been previously engineered to express luciferase upon IFN induction (*i.e.* B16.F10 IFN-LUC cells) (88), were next employed to assess whether the immunostimulatory activity of NanoISD was conserved in the non-immune, cancer cells and if so, to identify the *in vivo* kinetics of signaling. By quantifying luminescence *via* IVIS imaging following exposure to the substrate, D-luciferin, it was established that an *in vitro* treatment of NanoISD could activate luciferase production (*i.e.* IFN production) in the melanoma reporter cells, suggesting that the immunostimulatory capacity of the dsDNA was indeed conserved in the B16.F10 cell line (**Supplementary Figure 10**).

An intravital kinetics study of IFN production was subsequently performed to study the pharmacodynamics of NanoISD (**Figures 5E, F**). Mice were subcutaneously inoculated with the B16.F10 IFN-LUC cells, and when the tumors were ~ 50 mm^3^, mice were given a single intratumoral injection of either PBS or NanoISD. At preselected timepoints, mice were administered D-luciferin, and luminescence was measured 15 minutes thereafter. The longitudinal IVIS imaging confirmed *in vivo* IFN production with peak protein production occurring 12 hours post treatment. The level of *in vivo* IFN signaling returned to baseline at 24 hours post treatment despite the extended local retention profile of NanoISD. Therefore, though NanoISD is likely still present and intact within the tumor, we suspect that over time other factors, such as inhibitory pathways within cells or extracellular exclusion (*e.g.* fibrotic entrapment), might inactivate the nanoparticle complex and/or locally down regulate IFN signaling. Moreover, cancer cell stress or death induced by the treatment may also contribute to the decreased IFN signal over time, especially since the cancer cells are serving as the IFN reporter. Regardless, the acute IFN activity of NanoISD *in vivo* motivates the use of a therapeutic dosing regimen involving multiple injections spaced days apart [*e.g.* every three days (*q3d*)].

### NanoISD Reprograms the Immune Profile of the Tumor Microenvironment

**Figure 6:**
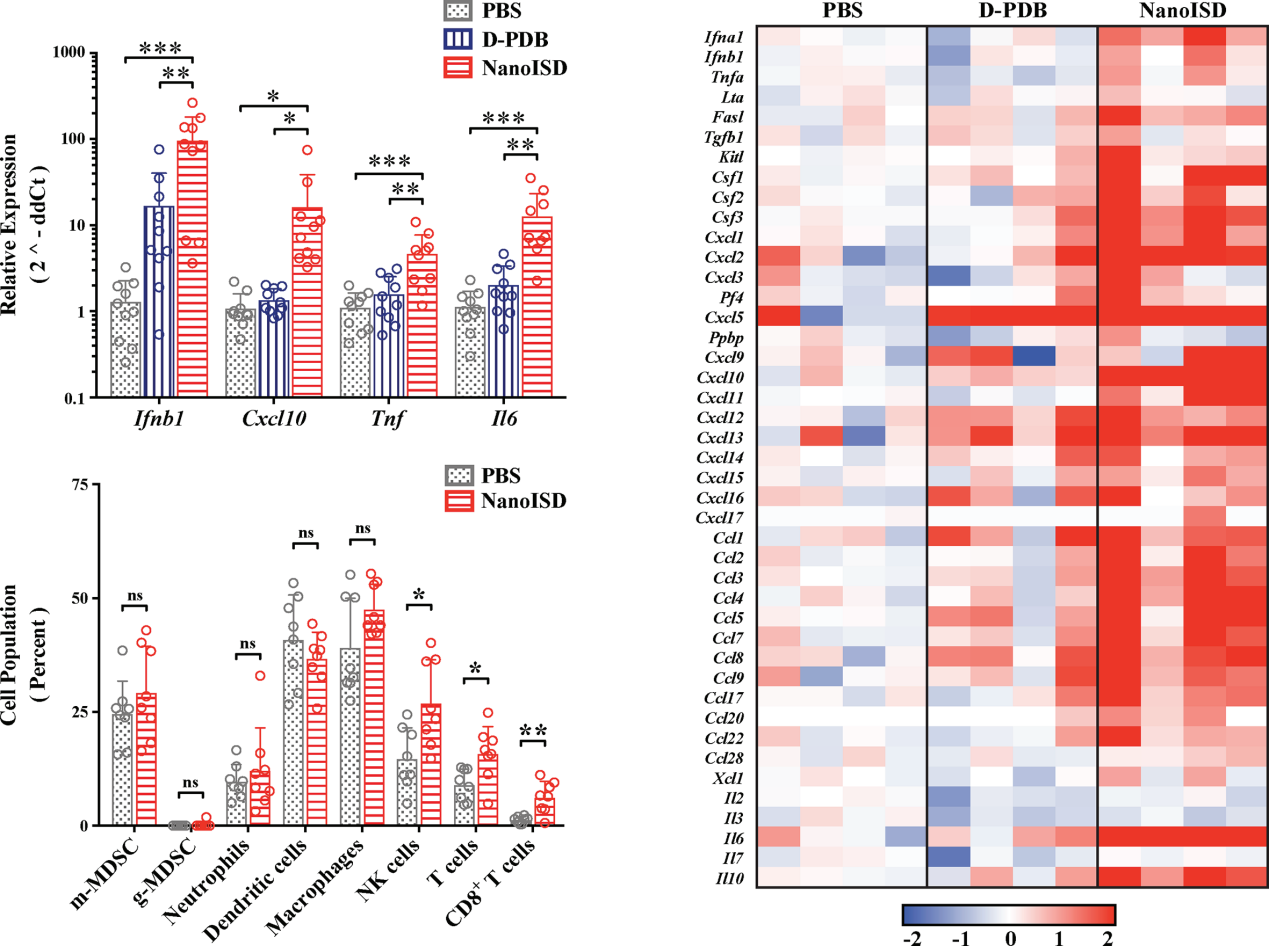
NanoISD Reprograms the Immune Profile of the Tumor Microenvironment. **(A)** Quantitative polymerase chain reaction (qPCR) analysis of B16.F10 tumors 6 hours following a single 100µL intratumoral treatment of either PBS, D-PDB, or NanoISD at a dose corresponding to 2 µg DNA. A one-way ANOVA with Tukey test was used for statistical analysis. **(B)** NanoString analysis of B16.F10 tumors 6 hours following a single 100µL intratumoral treatment of either PBS, D-PDB, or NanoISD at a dose corresponding to 2 µg DNA. Data is presented as log2 fold change relative to PBS treatment. **(C)** Flow cytometry analysis of the cellular composition of B16.F10 tumors treated intratumorally with 100 µL of either PBS or NanoISD at a dose corresponding to 2 µg DNA. Tumors were harvested 48 hours after the third intratumoral injection of a *q3d* dosing regimen. Data is presented as percent of CD45^+^ live cells. A two-way ANOVA with Sidak test was used for statistical analysis. ***p < 0.005, **p < 0.01, *p < 0.05. ns, not significant.

The immunological effects of intratumorally administered NanoISD were initially quantified by measuring changes in the gene expression of certain signature cytokines for STING pathway activation. B16.F10 tumors were harvested 6 hours after a single intratumoral treatment of either PBS, D-PDB, or NanoISD, and the relative mRNA levels of *Ifnb1*, *Cxcl10*, *Tnf*, and *Il6* in the tumor were determined *via* quantitative polymerase chain reaction (qPCR) (**Figure 6A**). The relative gene expression of these proinflammatory molecules was significantly elevated as compared to that of tumors treated with either PBS or free D-PDB, which is in accordance with STING pathway activation in the TME (89). Free D-PDB also exhibited increased *Ifnb1* expression, though not to the extent of NanoISD treatment, which is consistent with the *in vitro* activity assays that indicated that the D-PDB polymer acts as a weak cGAS adjuvant.

NanoString gene expression analysis was subsequently performed to provide a more robust transcriptomic analysis of the immune response in the treated tumors (**Figure 6B**). Using a slight variation of a gene expression panel that had been previously developed for myeloid cell characterization (90), exact mRNA levels were quantified for 43 different immunomodulatory cytokines. As determined by one-way ANOVA main effect, a single intratumoral NanoISD treatment upregulated the myeloid activation markers of the panel relative to PBS treatment (*i.e.* p = 0.0376) and D-PDB treatment (*i.e.* p = 0.0596). Notably, cytokines involved in myeloid recruitment (*i.e. Cxcl1*, *Cxcl2*, *Cxcl3*), myeloid differentiation (*i.e. Csf1*, *Csf2*, *Csf3*), and T cell recruitment (*i.e. Cxcl9*, *Cxcl10*, *Cxcl11*, *Cxcl12*) were markedly upregulated in the TME after NanoISD treatment. Additionally, D-PDB treatment was insignificantly different from PBS treatment (*i.e.* p = 0.9809) with regard to the myeloid activation markers of the panel. These results from the NanoString study further support the qPCR findings and provide additional insight into the immune profile of the treated tumors, demonstrating that a proinflammatory phenotype is indeed induced by intratumorally administered NanoISD.

To characterize the immunocellular changes within the TME that were likely to follow the local cytokine response, flow cytometry was conducted on B16.F10 tumors 48 hours after the final injection of a three treatment *q3d* dosing regimen (**Figure 6C**). Cell populations of interest were quantified using a myeloid cell panel (**Supplementary Figure 11**) and a T cell panel (**Supplementary Figure 12**). No marked changes occurred for the tumor populations of macrophages (*i.e.* CD45^+^ CD11b^+^ F4/80^+^), dendritic cells (*i.e.* CD45^+^ CD11c^+^ MHC-II^+^), monocytic myeloid-derived suppressor cells (m-MDSCs) (*i.e.* CD45^+^ CD11b^+^ Ly6C^+^), granulocytic MDSCs (g-MDSCs) (*i.e.* CD45^+^ CD11b^+^ Ly6G^+^ SSC ^hi^), and neutrophils (*i.e.* CD45^+^ CD11b^+^ Ly6G^+^, SSC ^lo^). However, the relative concentrations of NK cells (*i.e.* CD45^+^ NK1.1^+^), total T cells (*i.e.* CD45^+^ CD3^+^), and CD8^+^ T cells (*i.e.* CD45^+^ CD3^+^ CD8^+^) within the tumor were significantly elevated following NanoISD treatment, consistent with the established effects of STING pathway activation in tumors (14, 22, 23, 26). Thus, NanoISD can also propagate the adaptive arm of the cancer immunity cycle *via* the initial activation of innate immunity.

In addition to altering the migration and proliferation of lymphoid-derived immune cells, STING activation can also lead to improved cytotoxic immune responses by repolarizing immunosuppressive M2-like macrophages to M1-like macrophages that can promote antitumor immunity (91, 92). Thus, while not assessed in this work, it is possible that NanoISD also induces the M1-like phenotype in tumor macrophages, thereby further enhancing the antitumor immunity that is stimulated by NanoISD. Future work could study exactly how NanoISD affects macrophage polarization and the importance of such effects.

### NanoISD Exerts Antitumor Effects

Cancer therapy studies were conducted in murine tumor models to establish the therapeutic effect of NanoISD. Initially, the antitumor effects of NanoISD and free D-PDB were investigated in a poorly immunogenic B16 model of melanoma that has been engineered to express the foreign antigen, OVA (*i.e.* B16-OVA) in order to increase its antigenicity and therefore potential to respond to cancer immunotherapies. Mice bearing B16-OVA murine melanoma tumors were intratumorally treated with either PBS, D-PDB, or NanoISD for a total of three injections administered *q3d* (**Supplementary Figure 13**). Notably, NanoISD significantly restricted tumor growth and prolonged survival relative to both free D-PDB and PBS, which is consistent with a previous finding that phosphorothioate-capped dsDNA delivered intratumorally with a cationic transfection agent can mediate antitumor immune effects in the B16-OVA tumor model (93). Additionally, while D-PDB acts as a weak cGAS adjuvant, the free polymer did not demonstrate therapeutic efficacy *in vivo*, suggesting that the intrinsic effects of the D-PDB are insufficient to initiate STING-driven antitumor immune programs in the TME.

**Figure 7:**
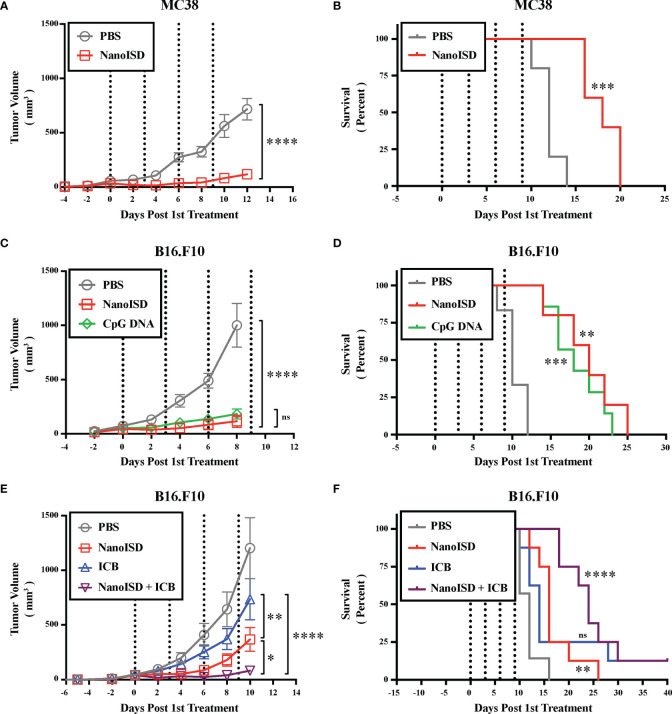
NanoISD Exerts Antitumor Effects. **(A)** Tumor growth plot for MC38 tumors intratumorally treated with 100 µL of either PBS or NanoISD at a dose corresponding to 2 µg DNA (n = 5 per treatment group). Treatments were administered four times *q3d* as indicated by the dotted lines. Tumor growth curves were truncated to the first day that a mouse in any treatment group reached the study endpoint. A two-way ANOVA with Sidak test was used for statistical analysis. The statistical analysis presented is for the final day shown (*i.e.* day 12). **(B)** Kaplan-Meier survival curve for MC38 tumors intratumorally treated with 100 µL of either PBS or NanoISD. Log rank (Mantel-Cox) test was used for statistical analysis. **(C)** Tumor growth plot for B16.F10 tumors intratumorally treated with 100 µL of either PBS, CpG DNA (*i.e.* ODN 1826), or NanoISD (n = 5 or greater per treatment group). Both the CpG DNA and NanoISD doses corresponded to 2 µg DNA. Treatments were administered four times *q3d* as indicated by the dotted lines. Tumor growth curves were truncated to the first day that a mouse in any treatment group reached the study endpoint. A two-way ANOVA with Tukey test was used for statistical analysis. The statistical analysis presented is for the final day shown (*i.e.* day 8). **(D)** Kaplan-Meier survival curve for B16.F10 tumors intratumorally treated with 100 µL of either PBS, CpG DNA (*i.e.* ODN 1826), or NanoISD. Log rank (Mantel-Cox) test was used for statistical analysis. **(E)** Tumor growth plot for B16.F10 tumors treated with 100 µL of either PBS, ICB (*i.e.* anti-PD-1 + anti-CTLA-4 monoclonal antibody therapy), NanoISD, or NanoISD + ICB (n = 8 per treatment group). NanoISD and PBS were administered intratumorally, while ICB was administered intraperitoneally. The NanoISD dose corresponded to 2 µg DNA. The ICB treatment corresponded to 100 µg of both anti-PD-1 and anti-CTLA-4 monoclonal antibodies. Treatments were administered four times *q3d* as indicated by the dotted lines. Tumor growth curves were truncated to the first day that a mouse in any treatment group reached the study endpoint. A two-way ANOVA with Tukey test was used for statistical analysis. The statistical analysis presented is for the final day shown (*i.e.* day 10). **(F)** Kaplan-Meier survival curve for B16.F10 tumors treated with 100 µL of either PBS, ICB (*i.e.* anti-PD-1 + anti-CTLA-4 monoclonal antibody therapy), NanoISD, or NanoISD + ICB (*i.e.* anti-PD-1 + anti-CTLA-4 monoclonal antibody therapy). Log rank (Mantel-Cox) test was used for statistical analysis. ****p < 0.0001, ***p < 0.005, **p < 0.01, *p < 0.05. ns, not significant.

NanoISD was subsequently explored as a therapeutic treatment for the less immunogenic tumor models, B16.F10 murine melanoma and MC38 murine colon cancer, both of which lack the expression of a foreign antigen (**Figure 7**). Treatments were again intratumorally administered *q3d* for a total of four injections. Relative to PBS-treated controls, the NanoISD treatment attenuated tumor growth (**Figures 7A, C**), prolonged murine survival (**Figures 7B, D**), and was well-tolerated by mice as demonstrated by insignificant differences in total mouse weight over time (**Supplementary Figure 14**). Furthermore, in the B16.F10 model, NanoISD treatment performed comparably to the well-established innate immune activator, CpG DNA when administered at the same dose (*i.e.* 2 µg DNA) (**Figures 7C, D**).

TLR9 agonists can function in a similar manner to that of cGAS/STING pathway agonists by promoting the cancer immunity cycle. Indeed, CpG DNA can induce B16 tumor regression in mice *via* NK cell-dependent, tumor antigen-specific T cell cross-priming (94). Accordingly, CpG DNA is also currently being investigated in human clinical trials for the treatment of cancer and they have recently demonstrated great potential for overcoming PD-1 blockade resistance in humans with advanced melanoma (95). However, CpG DNA relies on the cellular expression of TLR9, which is mostly restricted to plasmacytoid dendritic cells and B cells in humans (96). Alternatively, both the cGAS and STING proteins are rather ubiquitously expressed in mammalian cells (97–99). Moreover, TLR9 signaling can only occur in cells that are directly exposed to CpG DNA, while STING signaling can locally propagate from cell-to-cell *via* endogenous cGAMP transfer following DNA-induced cGAS activation (100–102). Thus, the cGAS/STING pathway might represent a more accessible pathway for promoting antitumor immunity *via* cytosolic DNA sensing. Regardless, cGAS/STING pathway agonists increase the arsenal of potential immunotherapeutic treatments, which can dramatically enhance overall patient outcomes by providing more opportunities for application-specific treatments. For example, CpG-based immunotherapy can impair the antitumor activity of BRAF inhibitors in a B cell–dependent manner when used in combination to treat cancer (103), whereas STING agonists can actually sensitize melanoma cells to BRAF inhibitors (104) and might thereby improve therapeutic efficacy in such a scenario.

To determine the impact of NanoISD treatment on the therapeutic response to ICB treatment (*i.e.* combined anti-PD-1 and anti-CTLA-4 monoclonal antibody therapy), B16.F10-bearing mice were treated with either PBS, NanoISD, ICB, or a combination of NanoISD and ICB for a total of four injections administered *q3d* (**Figures 7E, F**). Notably, the NanoISD treatment outperformed the ICB treatment, and the combination treatment of NanoISD and ICB was most effective at inhibiting the growth of treated tumors, indicating that NanoISD treatment can indeed improve therapeutic responses to murine ICB therapy. We note that there is still much room for improvement regarding the therapeutic efficacy of NanoISD, as the treatment in combination with ICB resulted in only one complete response (*i.e.* complete tumor elimination), matching that of ICB alone.

Since NanoISD consists of a self-assembling multi-phasic structure and is highly amenable to the integration of reactive handles (105), it should support various chemical and biomolecular engineering strategies to co-deliver multiple therapeutic agents (*e.g.* potentiators of the cGAS/STING pathway). One potential strategy for increasing the efficacy of NanoISD could include coupling NanoISD treatment with MEK inhibition or CXCR2 inhibition in order to block the expression and/or function of potentially undesirable cytokines (*e.g.* CXCL1 and CXCL2) that can enhance MDSC activity (90) and thereby reduce immune-mediated tumor clearance. Indeed, such a strategy has been previously employed to alleviate certain immunosuppressive effects of STING signaling that can accompany STING agonists and radiotherapy (106).

Other future considerations for NanoISD might involve further improving upon the design of the cGAS ligand and/or the cytosolic delivery agent as well as exploring strategies that could enable intravenous administration and/or tumor targeting of the cGAS agonist.

One variable not examined in this work is whether NanoISD activates other intracellular DNA sensors, such as AIM2, which can limit the magnitude of STING signaling upon *in vitro* stimulation (107). Future studies could investigate whether AIM2 is involved in the response to NanoISD. We note that it is unlikely that AIM2 plays a large role in the response to NanoISD, since the BP length threshold for robust AIM2 activation *in vitro* (*i.e.* ~ 150-BP) is greater than that of the optimized cGAS ligand (*i.e.* 95-BP) (4). However, if AIM2 is involved in the response to NanoISD, strategies could be employed to reduce AIM2 activation with the goal of enhancing STING signaling.

In this work, D-PDB was employed because of its previous success as a vehicle for the cytosolic delivery of immunostimulatory nucleic acids. Indeed, in multiple murine tumor models, an intratumoral treatment regimen of D-PDB loaded with immunostimulatory 5′ triphosphate RNA demonstrated significant therapeutic efficacy by promoting the activation of RIG-I (*i.e.* another cytosolic PRR that can drive antitumor immunity) (49, 50). While the work in this paper demonstrates that D-PDB can also be used to induce a therapeutic response *via* the cytosolic delivery of ISD and the pharmacological activation of cGAS, it is possible that other nanocarriers may elicit enhanced ISD delivery and improved therapeutic responses. Thus, future work aimed at improving therapeutic efficacy could explore the comparison of other nanocarriers for the cytosolic delivery of the optimized ISD (*i.e.* phosphorothioate-capped 95-BP dsDNA).

Lastly, we note that NanoISD may also have utility in other therapeutic areas (*e.g.* vaccinations for infectious diseases), as the DNA/polymer complex is a versatile adjuvant that can indiscriminately generate a local proinflammatory response, which can be advantageous for treating various diseases.

## Conclusion

Through an iterative experimental screen, the nucleic acid immunotherapeutic, NanoISD was engineered to trigger local cGAS/STING signaling *via* DNA-induced activation of the cGAS enzyme within the cytosol. The effects of formulation conditions (*i.e.* N/P charge ratio), DNA molecular weight (*i.e.* BP length), and DNA composition (*i.e.* phosphorothioate modifications) were investigated using a rationally designed synthetic ISD library in combination with a pH-responsive, endosome-destabilizing polymeric delivery vehicle. This yielded a potent nanoparticulate cGAS adjuvant that can evade major deoxyribonucleases, enhance cellular uptake, promote cytosolic delivery *via* endosomal escape, and trigger the cGAS/STING pathway in a cGAS-directed manner. Furthermore, NanoISD induces proinflammatory cytokine production, prompts the maturation of antigen presenting cells, promotes the tumor infiltration of NK cells and CD8^+^ T cells, reduces tumor burden, and enhances responses to ICB therapy. Thus, NanoISD represents a novel immunostimulant with clear indications for the treatment of immunologically cold cancers.

## Materials and Methods

### Polymer Synthesis and Characterization

Reversible addition-fragmentation chain transfer (RAFT) polymerization was employed to synthesize the amphiphilic diblock copolymer, *poly*[dimethylaminoethyl methacrylate]_10kDa_-*block*-[(propylacrylic acid)_0.3_-*co*-(dimethylaminoethyl methacrylate)_0.3_-*co*-(butyl methacrylate)_0.4_]_35kDa_ [*p*(DMAEMA)_10kDa_-*bl*-(PAA_0.3_-*co*-DMAEMA_0.3_-*co*-BMA_0.4_)_35kDa;_ D-PDB] as previously described (36). Briefly, the chain transfer agent (CTA) and mass initiator for the RAFT polymerizations were 4-cyano-4-(ethylsulfanylthiocarbonyl)sulfanylpentanoic acid (ECT; Boron Molecular) and 2,2’-azobis(4-methoxy-2,4-dimethyl valeronitrile) (V-70; Wako Chemicals), respectively. An analytical mass balance (XSE205DU DualRange; Mettler Toledo) was used for all mass measurements. Inhibitors were removed from monomer stocks by gravity filtration in columns that were packed with aluminum oxide.

For the first block of the polymer, filtered dimethylaminoethyl methacrylate (DMAEMA) was added to measured CTA in a glass vial with a target degree of polymerization of 100. A mass initiator stock was prepared by dissolving the initiator in the reaction solvent, dioxane. An appropriate amount of the mass initiator stock was added to the solution of CTA and DMAEMA at a molar ratio of 100:1:0.05 representing total monomer, CTA, and initiator, respectively. Additional dioxane was then added to the reaction vessel to attain a 40 wt% monomer solution. The solution was sealed and purged with nitrogen gas for 30 minutes on ice and then allowed to react at 40°C in an oil bath.

The reaction was stopped after 22 hours by opening the reaction vessel and exposing the mixture to air. The resultant polymer was then purified by precipitation into cold pentane and subsequent dialysis. The crude product was precipitated six times by transferring the polymer solution into cold pentane. Centrifugation (5000 rpm, 5 min, 4°C) was used to pellet the polymer mixture, and the supernatant was then discarded. Small volumes of acetone were added to dissolve the pelleted polymer, thereby enabling the polymer to be transferred to new precipitation tubes. The polymer mixture was then collected in a 3.5 MWCO SnakeSkin™ dialysis membrane (Cat. No. 68035; Thermo Fisher Scientific) and further purified *via* membrane dialysis against pure acetone (3x), half-acetone and half deionized water (2x), and then pure deionized water (2x) for 4 hour intervals each. Following dialysis, poly(DMAEMA) was frozen at -80°C for 5 hours and then lyophilized for 3 days.

For the second block of the polymer, poly(DMAEMA) was used as a macroCTA (mCTA). Filtered DMAEMA, PAA, and BMA (at a molar ratio of 30:30:40) were added to measured mCTA in a glass vial with a target degree of polymerization of 450. PAA was synthesized using diethyl propylmalonate as the precursor as previously described (108). A mass initiator stock was prepared by dissolving the initiator in the reaction solvent, N,N-dimethylacetamide (DMAC). An amount of the mass initiator stock was added to the solution of mCTA and monomers at a molar ratio of 450:1:0.4 representing total monomer, mCTA, and initiator, respectively. Note that a greater Initiator/CTA ratio is required to get PAA to incorporate into the polymer chains. Additional DMAC was then added to the reaction vessel to attain a 40 wt% mCTA and monomer solution. The solution was sealed and purged with nitrogen gas for 30 minutes on ice and then allowed to react at 40°C in an oil bath.

The reaction was stopped after 24 hours by opening the reaction vessel and exposing the mixture to air. The resultant polymer was then purified by precipitation into cold pentane:ether (80:20) and subsequent dialysis. The crude product was precipitated six times by transferring the polymer solution into cold pentane:ether (80:20). Centrifugation (5000 rpm, 5 min, 4°C) was used to pellet the polymer mixture and remove the supernatant. Again, small volumes of acetone were added to dissolve the pelleted polymer, thereby enabling the polymer to be transferred to new precipitation tubes. The polymer mixture was then collected in a 10 MWCO SnakeSkin™ dialysis membrane (Cat. No. 68100; Thermo Fisher Scientific) and further purified *via* membrane dialysis against pure acetone (3x), half-acetone and half deionized water (2x), and then pure deionized water (2x) for 4 hour intervals each. Following dialysis, poly(DMAEMA) was frozen at -80°C for 5 hours and then lyophilized for 3 days. All lyophilized polymer was stored at -20°C prior to use.

^1^H NMR Spectroscopy (CDCl_3_ with TMS, 400 MHz) was used to calculate the experimental degree of polymerization, polymer composition, and theoretical molecular weight of the polymers (**Supplementary Figure 15**). Subsequently, the experimental molecular weight and a polydispersity index were measured by gel permeation chromatography (GPC) (mobile phase HPLC-grade dimethylformamide (DMF) containing 0.1% LiBr) with inline light scattering (Wyatt Technology) and refractive index (Agilent) detectors (**Supplementary Figure 16**). The ASTRA V Software (Wyatt Technology) was used for all GPC-related calculations. Additionally, The *poly*(dimethylaminoethyl methacrylate)_10kDa_-*block*-(butyl methacrylate)_34kDa_ (*p*DMAEMA_10kDa_-*bl*- BMA_34kDa;_ D-B) polymer was previously prepared (50).

Near-infrared D-PDB (NIR-D-PDB) was created by labeling D-PDB with NIR-664-iodoacetamide (CAS 149021-66-9; Santa Cruz) at a molar ratio of 1:1. Briefly, 72 µL of a 12.5 mg/mL stock of NIR-664-iodoacetamide dissolved in methanol was added to 50 mg of D-PDB dissolved in 1 mL methanol. The mixture was vortexed, and the reaction was allowed to proceed at room temperature overnight while continuously stirring and protected from light. The mixture was then transferred to a 3.5 MWCO SnakeSkin™ dialysis membrane (Cat. No. 68035; Thermo Fisher Scientific) and purified *via* membrane dialysis against pure methanol (3x), half-methanol and half deionized water (2x), and then pure deionized water (2x) for 4 hour intervals each, all the while kept at 4°C and protected from light. Following dialysis, the sample was run through a PD-10 desalting column (17085101; Cytiva) into H_2_O. The fully purified sample was frozen at -80°C for 5 hours and then lyophilized for 2 days. NIR-D-PDB was stored at -20°C prior to use.

### Preparation of ISD Libraries

The synthetic library of phosphorothioate-capped dsDNA (**Supplementary Figure 1**) and other associated DNA sequences were purchased as a duplex from Integrated DNA Technologies (IDT) unless otherwise specified. The second ISD library of PCR-amplified dsDNA (**Supplementary Figure 2**) was prepared as follows. The 10,183-BP lentiGuide-Puro plasmid (Plasmid #52963; Addgene) was used to generate custom BP length dsDNA PCR products. In brief, the lentiGuide-Puro agar stab was spread over standard 0.5 mg/mL puromycin agar plates and placed in a 37°C bacteria incubator overnight. The following day, individual bacteria colonies were isolated and placed in liquid LB broth with 0.5 mg/mL puromycin, swirled, loosely covered with sterile cap, and left to incubate at 37°C for 12 hours. Bacteria growths were purified with the QIAprep Miniprep kit (Cat. No. 27104; Qiagen), resuspended in sterile H_2_O, and DNA concentration was quantified by ultraviolet–visible (UV-Vis) spectroscopy (Nanodrop 2000 Spectrophotometer; Thermo Fisher Scientific).

Forward and reverse primers were designed using the NCBI Primer Blast tool for dsDNA sequences of variable BP length (*i.e.* 95, 156, 313, 625, 1250, 2500, 5000, and 10000 BP). For the PCR-amplification of each length of dsDNA, individual reactions were set up with 4 µL of 5x Phusion GC Buffer, 0.4 µL of 10 mM dNTPs (D7295; MilliporeSigma), 1 µL of 10 µM forward primer, 1 µL of 10 µM reverse primer, 0.6 µL of DMSO, 0.2 µL of Phusion^®^ High-Fidelity DNA Polymerase (M0530; New England Biolabs), 4 µL of 1 ng/µL (4 ng) of lentiGuide-Puro plasmid template DNA (Plasmid #52963; Addgene), and 8.8 µL of H_2_O, per 20 µL reaction. Thermocycling conditions were 98°C for 30 seconds, followed by 35 cycles of 98°C for 10 seconds, 54°C for 30 seconds, 72°C for 30 seconds per kb of PCR length, followed by 72°C for 10 minutes. PCR products were concentrated using standard ethanol precipitation and clear bands were observed on a 2% agarose gel for each PCR length. Each PCR-amplified product was stored at -20°C prior to use.

### Nanoparticle Formulation

Lyophilized D-PDB was dissolved in ethanol to 50 mg/mL. Aliquots of this polymer stock were then diluted in phosphate buffer (pH 7.0, 100 mM) to a concentration of 10 mg/mL, allowing the polymer chains to self-assemble into micelles. The 10 mg/mL polymer solution was then concentrated into PBS (pH 7.4; Gibco) through 4 cycles of centrifugal filtration with Amicon^®^ Ultra 0.5 mL Centrifugal Filter Units (Ultracel^®^ - 3K, Regenerated Cellulose 3,000 NMWL; MilliporeSigma) following manufacturer’s instructions. The final concentrated polymer solution was collected, and an aliquot was taken to determine the polymer concentration relative to a standard curve. Using a 96-well plate (REF 655180; Greiner Bio-One), the polymer concentration was calculated from UV-vis spectroscopy (Synergy H1 Multi-Mode Microplate Reader; Biotek) based on absorbance at 310 nm. The micelle solution was diluted to 1 mg/mL with PBS and passed through a 0.2 µm Whatman^®^ Puradisc polyethersulfone sterile filter (WHA67801302; MilliporeSigma). A fixed amount of the sterile-filtered polymer stock was then added to an aqueous solution containing a set amount of nucleic acid, which corresponded to the desired N/P charge ratio. Again, note that the first block of the diblock copolymer composed of poly(DMAEMA) is estimated to exhibit 50% protonation at pH 7.4 for the purposes of determining N/P ratios. Upon the addition of the polymer micelles to the nucleic acid, the solution was rapidly mixed by pipetting and then incubated at room temperature for 20 minutes to allow for complete electrostatic complexation.

### Nanoparticle Physical Characterization

Hydrodynamic size of the polymeric micelles and DNA/polymer complexes was measured *via* digital light scattering (DLS) using either the Zetasizer Nano ZS instrument (Malvern Panalytical) or the Litesizer 500 instrument (Anton Paar) as indicated in figure captions. Additionally, the zeta potential of the polymeric micelles and DNA/polymer complexes was determined using the Zetasizer Nano ZS instrument (Malvern Panalytical). Polymer concentrations were normalized to 1 mg/mL and samples were run at physiological pH 7.4. DNA concentrations correspond to the N/P charge ratios, which were set to 4 unless otherwise indicated.

2% agarose gels were prepared by dissolving 3 grams of UltraPure™ Agarose powder (16500100; Thermo Fisher Scientific) in 150 mL of 1x TAE buffer that had been diluted with deionized H_2_O from a 10x TAE buffer stock (REF 46010CM; Corning). The mixture was microwaved in 30 second intervals until the agarose was fully dissolved. The solution was then cast into a gel. DNA and DNA/polymer complexes were then prepared. For the DNase I activity experiment, the indicated concentrations of DNase I (M0303; New England Biolabs) were incubated with the indicated samples for 15 minutes at 37°C. The resultant mixtures and controls were then incubated at 75°C for 15 minutes to heat-inactivate the DNase I, and a volume of 10% sodium dodecyl sulfate (SDS) (RGE3230; K-D Medical) was subsequently added to the mixtures and controls such that a final concentration of 1% SDS was achieved, which allowed for decomplexation of the DNA from the polymer. All of the samples were mixed with a volume of glycerol such that a final concentration of 5% glycerol was achieved prior to gel loading. Samples were loaded into wells of the agarose gel at a concentration of 1 µg DNA/lane. Polymer concentrations correspond to the indicated N/P charge ratio. The TrackIt™ 100 bp DNA Ladder (Cat. No. 10488058; Thermo Fisher Scientific), the TrackIt™ 1 Kb Plus DNA Ladder (Cat. No. 10488085; Thermo Fisher Scientific), or the NEB 1 kb DNA Ladder (N3232; New England Biolabs) were used for references as indicated in figure captions. Gel electrophoresis was then performed at 120 V for 45 minutes. Gels were subsequently stained with SYBR Safe dye (S33102; Thermo Fisher Scientific) for 30 minutes while protected from light and then imaged with a Digital ChemiDoc MP system (Bio-Rad).

### Cell Lines

All cell lines were maintained according to supplier specifications and/or technical data sheets. RAW-Dual cells (InvivoGen) and RAW-Lucia ISG-KO-cGAS cells (InvivoGen) were cultured in Dulbecco’s Modified Eagle Medium (DMEM; Gibco) supplemented with 2 mM _L_-glutamine, 4.5 g/L glucose, 10% heat-inactivated fetal bovine serum (HI-FBS; Gibco), 100 U ml^−1^ penicillin/100 μg ml^−1^ streptomycin (Gibco), and 100 µg/mL Normocin. For the continual selection of these cell lines, Zeocin was added on every other cell passage at a concentration of 200 µg/mL. THP1-Dual cells (InvivoGen) and THP1-Dual KO-TREX1 cells (InvivoGen) were cultured in Roswell Park Memorial Institute (RPMI) 1640 Medium (Gibco) supplemented with 2 mM _L_-glutamine, 25 mM HEPES, 10% heat-inactivated fetal bovine serum (HI-FBS; Gibco), 100 U ml^−1^ penicillin/100 μg ml^−1^ streptomycin (Gibco), and 100 µg/mL Normocin. For the continual selection of these cell lines, Blasticidin and Zeocin were added after every cell passage at concentrations of 10 µg/mL and 100 µg/mL, respectively. A549-Dual cells (InvivoGen) were cultured in Dulbecco’s Modified Eagle Medium (DMEM; Gibco) supplemented with 2 mM _L_-glutamine, 4.5 g/L glucose, 10% heat-inactivated fetal bovine serum (HI-FBS; Gibco), 100 U ml^−1^ penicillin/100 μg ml^−1^ streptomycin (Gibco), and 100 µg/mL Normocin. For the continual selection of this cell line, Blasticidin and Zeocin were added after every cell passage at concentrations of 10 µg/mL and 100 µg/mL, respectively. DC2.4 cells were cultured in Roswell Park Memorial Institute (RPMI) 1640 Medium (Gibco) supplemented with 2 mM _L_-glutamine, 1× non-essential amino acids (Cellgro), 10 mM HEPES (Invitrogen), 50 μM 2-mercaptoethanol (Gibco), 10% heat-inactivated fetal bovine serum (HI-FBS; Gibco), and 100 U ml^−1^ penicillin/100 μg ml^−1^ streptomycin (Gibco). 4T1 cells (ATCC) were cultured in Dulbecco’s Modified Eagle Medium (DMEM; Gibco) supplemented with 2 mM _L_-glutamine, 4.5 g/L glucose, 10% heat-inactivated fetal bovine serum (HI-FBS; Gibco), and 100 U ml^−1^ penicillin/100 μg ml^−1^ streptomycin (Gibco). B16.F10 cells (ATCC) were cultured in Dulbecco’s Modified Eagle Medium (DMEM; Gibco) supplemented with 2 mM _L_-glutamine, 4.5 g/L glucose, 10% heat-inactivated fetal bovine serum (HI-FBS; Gibco), and 100 U ml^−1^ penicillin/100 μg ml^−1^ streptomycin (Gibco). B16.F10 IFN-LUC cells were cultured in Dulbecco’s Modified Eagle Medium (DMEM; Gibco) supplemented with 2 mM _L_-glutamine, 4.5 g/L glucose, 10% heat-inactivated fetal bovine serum (HI-FBS; Gibco), and 100 U ml^−1^ penicillin/100 μg ml^−1^ streptomycin (Gibco). Puromycin was added after every cell passage at a concentration of 10 µg/mL. B16-OVA cells were cultured in Dulbecco’s Modified Eagle Medium (DMEM; Gibco) supplemented with 2 mM _L_-glutamine, 4.5 g/L glucose, 10% heat-inactivated fetal bovine serum (HI-FBS; Gibco), and 100 U ml^−1^ penicillin/100 μg ml^−1^ streptomycin (Gibco). For the continual selection of this cell line, Geneticin (G418; Gibco) was added after every cell passage at a concentration of 500 µg/mL. MC38 cells were cultured in Dulbecco’s Modified Eagle Medium (DMEM; Gibco) supplemented with 2 mM _L_-glutamine, 0.1 mM non-essential amino acids (Cellgro), 10 mM HEPES (Invitrogen), 1 mM sodium pyruvate, 10% heat-inactivated fetal bovine serum (HI-FBS; Gibco), 100 U ml^−1^ penicillin/100 μg ml^−1^ streptomycin (Gibco), and 50 µg/mL gentamicin sulfate (Gibco). All cells lines were tested for Mycoplasma contamination and kept in a humidified environment with 5% CO at 37°C.

### *In Vitro* Reporter Cell Assays

96-well plates (REF 655180; Greiner Bio-One) were used for screening the DNA/polymer complexes. Reporter cells were seeded at 50,000 cells/well in 100 µL media. When cells became ~ 80% confluent, treatments were administered in 100 µL PBS. Results were collected 24 hours after treatment. Quanti-Luc™ and Quanti-Blue™ (InvivoGen) assays were performed on cell supernatants following manufacturer’s instructions. Luminescence and absorbance were quantified *via* plate reader (Synergy H1 Multi-Mode Microplate Reader; Biotek). Luminescence measurements were performed using white, opaque-bottom 96-well plates (REF 655073; Greiner Bio-One), and absorbance measurements were performed using standard, clear 96-well plates (REF 655180; Greiner Bio-One). The signal for each sample concentration was determined using 3 biological replicates, each with 3 technical replicates. For the *in vitro* IVIS assay with the B16.F10 IFN-LUC cells, black 96-well plates (REF 655096; Greiner Bio-One) were used, and luminescence measurements were performed on an IVIS Lumina III (PerkinElmer) 5 minutes after the addition of Pierce™ D-Luciferin, Monopotassium Salt (88293; Thermo Fisher Scientific) reconstituted in PBS, such that the final concentration of D-luciferin was 150 µg/mL. The *in vitro* IVIS experiment included 3 biological replicates without technical replicates. All reporter cell measurements were normalized by subtracting the average value of a PBS-treated negative control group. All bell-shaped dose response curves were truncated at their plateau. The EC_50_ and IC_50_ values were calculated for each of the dose responses using curve fitting analysis in the GraphPad Prism software.

### *In Vitro* Cellular Uptake Study

DC2.4 cells were seeded in 12-well plates (REF 665180; Greiner Bio-One) at 4 x 10^5^ cells/well and allowed to adhere overnight. Treatments of either PBS, DNA/D-PDB, Cy5-DNA, Cy5-DNA/D-PDB, NIR-D-PDB, or DNA/NIR-D-PDB were administered to the cells for 4 hours at 37°C with 5% CO_2_. Doses were set at 45 nM DNA (*i.e.* theoretical EC_50_ value for NanoISD in RAW-Dual cells normalized to surface area of the tissue culture area on the 12-well plate) and/or the corresponding concentration of polymer for an N/P charge ratio of 4. Following incubation, cells were trypsinized, washed, and resuspended with flow cytometry staining buffer (FACS buffer) (*i.e.* PBS + 2% FBS) supplemented with 1 µg/mL DAPI. Cells were then analyzed using an Amnis CellStream Luminex flow cytometer. Each treatment was performed with 4 technical replicates. Cellular uptake was also analyzed at 24 hours post treatment, and similar results were observed (*data not shown*).

### *In Vitro* BMDC Maturation Study

Bone marrow cells were harvested from femurs and tibias of 6-8 week-old female C57BL/6J mice by flushing them with cold PBS. Cells were centrifuged for 5 minutes at 450 x g and resuspended in complete BMDC culture media (*i.e.* RPMI 1640 medium supplemented with 10% HI FBS, 1% Pen-Strep (*i.e.* 100 U/mL penicillin and 100 µg/mL streptomycin), 2 mM L-glutamine, 10 mM HEPES, 1 mM sodium pyruvate, 1x non-essential amino acids, 50 μM β-mercaptoethanol, and 20 ng/mL GM-CSF). The cell suspension was passed through a 70 μM sterile cell strainer (22363548; Fisherbrand™; Thermo Fisher Scientific), and the cells were then seeded in 100x15 mm non-tissue-culture-treated petri dishes (REF 351029; Corning) and incubated at 37°C with 5% CO_2_. Fresh complete BMDC culture media was added on days 3, 5, and 7. On day 8, the percentage of CD11c^+^ cells (*i.e.* BMDCs) was confirmed to be greater than 80% as measured with by flow cytometry using anti-CD11c-FITC (Clone N418; BioLegend), and the BMDCs were then seeded in 12-well plates (REF 665180; Greiner Bio-One) at 6 x 10^5^ cells/well. Treatments of PBS, 45 nM phosphorothioate-capped 95-BP dsDNA (*i.e.* DNA), 2 µM MPLA, and 45 nM NanoISD (*i.e.* theoretical EC_50_ value for NanoISD in RAW-Dual cells normalized to surface area of the tissue culture area on the 12-well plate) were administered to the BMDCs for 24 hours at 37°C with 5% CO_2_. Following incubation, cells were scrapped, washed with FACs buffer, incubated with Fc-block (anti-CD16/CD32, Clone 2.4G2; Tonbo) for 15 minutes at 4°C, and then stained with antibodies against the markers of DC activation, anti-CD86-PE/Cy7 (Clone GL-1; BioLegend) and anti-MHC-II-APC/Cy7 (Clone M5.114.15.2; BioLegend) for 1 hour at 4°C. Cells were then washed 2x in FACS buffer, resuspended using FACS buffer supplemented with 1 µg/mL DAPI, and analyzed using an Amnis CellStream Luminex flow cytometer. Each treatment was performed with 4 technical replicates, and the experiment was conducted 3 times with similar results.

### *In Vivo* Imaging Experiments

All *in vivo* imaging was performed on the IVIS Lumina III (PerkinElmer). Mice were anesthetized with isoflurane gas and shaved around the injection site as necessary. For all *in vivo* retention experiments, fluorescence was recorded longitudinally as indicated, and corresponding fluorophore-specific filter pairs were used. For the subcutaneous retention study, 6-8 week-old CD-1 mice (Charles River Laboratories) were administered a single 100 µL subcutaneous injection of either PBS, Cy5-DNA, Cy5-DNA/D-PDB, NIR-D-PDB, or DNA/NIR-D-PDB on each rear flank. Individual uncomplexed fluorescent agents were administered on the left flank of the mice, and the complexes at an N/P charge ratio of 4 with the indicated fluorescent agent were administered on the right flank of the mice. Each treatment contained 2 µg DNA and/or the corresponding amount of polymer for an N/P charge ratio of 4. For the intratumoral retention study, 6-8 week-old BALB/c mice (The Jackson Laboratory) were orthotopically inoculated with 4T1 tumors by injecting 1 x 10^6^ cells suspended in 100 µL of a 1:1 mixture of PBS and Type 2 Cultrex Basement Membrane Extract (3532-005-02; R&D Systems) into the left inguinal mammary fat pad. When tumors were ~ 100 mm^3^, the mice were administered a single 100 µL intratumoral injection of either PBS, Cy5-labeled phosphorothioate-capped 95-BP dsDNA (*i.e.* Cy5-DNA), or Cy5-DNA/D-PDB. Each treatment contained 2 µg DNA and/or the corresponding amount of polymer for an N/P charge ratio of 4.

For the *in vivo* IFN activity experiment, 6-8 week-old C57BL/6 mice (The Jackson Laboratory) were inoculated with B16.F10 IFN-LUC tumors by subcutaneously injecting 1 x 10^6^ cells suspended in 100 µL of PBS into the rear right flank. When tumors were ~ 100 mm^3^, the mice were administered a single 100 µL intratumoral injection of either PBS or NanoISD at a 2 µg DNA dose. Luminescence was recorded at set time points (*i.e.* 0, 4, 8, 12, and 24 hours). For each timepoint, the mice were administered a dorsal subcutaneous 150 µL injection of 30 mg/mL Pierce™ D-Luciferin, Monopotassium Salt (88293; Thermo Fisher Scientific) reconstituted in PBS, and a luminescence image was captured 15 minutes thereafter.

### Quantitative RT-PCR and NanoString Analysis

6-8 week-old C57BL/6 mice (The Jackson Laboratory) were inoculated with B16.F10 tumors by subcutaneously injecting 1 x 10^6^ cells suspended in 100 µL of PBS into the rear right flank. When tumors were ~ 200 mm^3^, the mice were administered a single 100 µL intratumoral injection of either PBS, D-PDB, or NanoISD at a 2 µg DNA dose. 6 hours after the intratumoral injection, mice were euthanized and tumors were harvested. Tumors were then homogenized using TissueLyser II (Qiagen), and tumor RNA was isolated using the RNeasy^®^ Plus Mini Kit (Qiagen).

For the qPCR analysis of gene expression, 1 μg of the tumor RNA was reverse transcribed by an iScript cDNA synthesis kit (Bio-Rad) following the manufacturer’s instructions. The qPCR was conducted on the generated cDNA using a Bio-Rad CFX Connect Real-time System, with the threshold cycle number determined by Bio-Rad CFX manager software V.3.0. The following TaqMan gene expression kits (Thermo Fisher Scientific) were used following the manufacturer’s instructions: mouse *Ifnb1* (Mm00439552_s1); mouse *Cxcl10* (Mm00445235_ m1); mouse *Tnf* (Mm00443258_m1); mouse *Il6* (Mm00446190_m1); mouse *Ppib* (Mm00478295_m1). Reactions for each gene were performed in technical duplicate for ten biological samples per treatment group, and the threshold cycle numbers were averaged. Gene expression was normalized to the house-keeping gene, *Ppib* and then normalized to the PBS treatment values using the 2^-ddCt^ method of analysis.

For the NanoString analysis of gene expression, 100 ng of mRNA isolated from tumor tissue was hybridized to a myeloid panel of target-specific fluorescent barcodes. The hybridized samples were analyzed on the NanoString nCounter MAX Analysis system. Subsequent data processing was performed using the NanoString nSolver data analysis software.

### *In Vivo* Tumor Therapy Experiments

6-8 week-old C57BL/6 mice (The Jackson Laboratory) were inoculated with B16-OVA, B16.F10, or MC38 tumors by subcutaneously injecting 1 x 10^6^ cells suspended in 100 µL of PBS into the rear right flank. When tumors were ~ 50 mm^3^, the mice were given four 100 µL intratumoral injections administered *q3d* with treatments of either PBS, D-PDB, CpG DNA (*i.e.* ODN 1826), or NanoISD at a 2 µg DNA dose. For the therapy study with ICB, certain mice were also given four 100 µL intraperitoneal injections on the same days as the intratumoral treatments (*i.e.* administered *q3d*) with a treatment of the monoclonal antibodies, anti-PD-1 (RMP1-14, BE0146; Bio X Cell) and anti-CTLA-4 (9d9, BE0164; Bio X Cell). Tumor volume, total murine mass, and murine well-being were recorded *qod* for the duration of the study. The study endpoint for maximum tumor volume (*i.e.* survival) was 1500 mm^3^.

### Flow Cytometry

6-8 week-old C57BL/6 mice (The Jackson Laboratory) were inoculated with B16.F10 tumors by subcutaneously injecting 1 x 10^6^ cells suspended in 100 µL of PBS into the rear right flank. When tumors were ~ 50 mm^3^, the mice were given three 100 µL intratumoral injections administered *q3d* with treatments of either PBS or NanoISD at a 2 µg DNA dose. 48 hours after the final intratumoral injection, mice were euthanized and tumors were harvested. The tumors were then mechanically dissociated with an OctoMACS separator, and digested in a solution of 125 μg ml−1 Deoxyribonuclease I (Worthington) and 500 μg ml−1 Collagenase III (Worthington) in RPMI 1640 media for 30 minutes at 37°C. The digested tumors were strained through a 70 μM sterile cell strainer (22363548; Fisherbrand™; Thermo Fisher Scientific) and treated with ACK Lysing Buffer (Gibco).

The remaining tumor cells were washed and diluted to a concentration of 1 × 10^7^ cells/mL in FACS buffer supplemented with 50 nM dasatinib, and the cell suspension was aliquoted into a 96-well plate (REF 655180; Greiner Bio-One). 100 µl was add to each well with the number of wells filled corresponding to the number of flow cytometry tests to be performed. After another wash with FACS buffer supplemented with 50 nM dasatinib, the plated cells were incubated with Fc-block (anti-CD16/CD32, Clone 2.4G2; Tonbo) for 15 minutes at 4°C. The relevant fluorescent antibodies were then added for each flow cytometry test, and the cells were incubated for 45 minutes at 4°C while protected from light. Cells were washed twice, suspended in FACS buffer supplemented with 1 µg/mL DAPI, and then analyzed using a 5-laser LSRII flow cytometer (BD).

The samples were stained with the fluorescent antibodies of either a myeloid panel or T cell panel. The following antibodies were used for the myeloid panel: anti-CD45.2-APC (20-0454-U025; Tonbo), anti-CD11b-PerCp-Cy5.5 (550993; BD BioSciences), anti-NK-1.1-PE (108707; BioLegend), anti-F4/80-PE/Cy7 (123113; BioLegend), anti-MHC-II-APC/Cy7 (107628; BioLegend), anti-CD11c-PE/Cy5 (117316; BioLegend), anti-Ly-6G-A488 (127625; BioLegend), and anti-Ly-6C-BV605 (128035; BioLegend). The following antibodies were used for the T cell panel: anti-CD45.2-APC (20-0454-U025; Tonbo), anti-CD3e-PE/Cy7 (552774; BD BioSciences), and anti-CD8a-PE/Cy5 (100710; BioLegend). DAPI was used to discriminate live *versus* dead cells. Representative gating for each panel can be found in the Supplementary Information (**Supplementary Figures 11, 12**).

### Statistical Analysis

The significance for each experiment was determined as indicated in the corresponding figure caption. Statistical analyses were performed using GraphPad Prism software, version 7.0c. The plotted values represent the experimental means, and the error bars represent one standard deviation (SD), except for those in the tumor growth plots, which represent one standard error of the mean (SEM). ****p < 0.0001, ***p < 0.005, **p < 0.01, *p < 0.05.

## Data Availability Statement

The original contributions presented in the study are publicly available. This data can be found at https://www.ncbi.nlm.nih.gov/geo/under the following accession numbers: GSE183144, GPL30574 and GSM5552035-GSM555204.6.

## Ethics Statement

All animal experiments were reviewed and approved by the Vanderbilt University Institutional Animal Care and Use Committee (IACUC), and all surgical and experimental procedures were performed in accordance with the regulations and guidelines of the Vanderbilt University IACUC. All mice were maintained at the animal facilities of Vanderbilt University under pathogen-free conditions.

## Author Contributions

Conceptualization was performed by KG and JW. Methodology was done by KG, JR, CC, and SS. Formal analysis was performed by KG. Experiments were performed by KG, CC, LW-B, AH, and CK. Resources were provided by JR, CC, SS, JB, MA, and JW. Data visualization and figure preparation was done by KG. The original draft was written by KG. The manuscript was reviewed and/or edited by JR, CC, LW-B, AH, SS, CK, JB, MA, and JW. Funding was acquired by JW. All authors contributed to the article and approved the submitted version.

## Funding

This research was supported by grants from the National Science Foundation CBET-1554623 (JW), a Vanderbilt Ingram Cancer Center (VICC) Ambassador Discovery Grant (JW), an American Cancer Society Institutional Research Grant (IRG-58-009-56 to JW), the Congressionally-Directed Medical Research Program (W81XWH-161-0063 to JW), and a Stand Up To Cancer Innovative Research Grant, Grant Number SU2C-AACR-IRG 20-17 (JW). Stand Up To Cancer (SU2C) is a program of the Entertainment Industry Foundation. Research grants are administered by the American Association for Cancer Research, the scientific partner of SU2C. CC acknowledges a National Science Foundation Graduate Research Fellowship under grant numbers DGE-1445197 and DGE-1937963. CK was supported by the Vanderbilt Institute for Infection, Immunology, and Immunity (VI4) Summer Research Scholars Program - Class of 2019.

## Conflict of Interest

The authors declare that the research was conducted in the absence of any commercial or financial relationships that could be construed as a potential conflict of interest.

## Publisher’s Note

All claims expressed in this article are solely those of the authors and do not necessarily represent those of their affiliated organizations, or those of the publisher, the editors and the reviewers. Any product that may be evaluated in this article, or claim that may be made by its manufacturer, is not guaranteed or endorsed by the publisher.
