# Supplementary material for: Pharmacological Activation of cGAS for Cancer Immunotherapy
Source: Front Immunol. 2021 Nov 26;12:753472. doi: 10.3389/fimmu.2021.753472 (PMC8662543; doi:10.3389/fimmu.2021.753472)
Supplement: Supplementary file 1 [file DataSheet_1.pdf]

frontiers in Immunology

Supplementary Material

Sequence 1: **Phosphorothioate-capped 95-BP dsDNA**
A*C*A*CACACACACACACACACCATAA**AACAACAACAACAACAACAA**CACCA**ACACACACACACACACACAC**CAACA**AACAACAACAACAACAA*C*A*A**

Secondary Structure Prediction Website: No Structures to Write

Complementary Sequence 1:
T*T*G*TTGTTGTTGTTGTTTGTTGGTGTGTGTGTGTGTGTGTTGGTGTTGTTGTTGTTGTTGTTGTTTTATGGTGTGTGTGTGTGTG*T*G*T

Secondary Structure Prediction Website: Delta G = **+8.1** (unlikely to create any secondary structures)

Melting Temperature Prediction Website: GC content = 40 % ; T_M = **74 ºC**
IDT OligoAnalyzer Tool Website: Maximum Delta G (*i.e.* free energy of the oligo sequence binding to its perfect complement) = **-163.94 kcal/mole**

Sequence 2: **Phosphorothioate-capped 70-BP dsDNA**
A*C*A*CACACACACACACACACCATAA**AACAACAACAACAACAACAA**CACCA**ACACACACACACACACA*C*A*C**

Secondary Structure Prediction Website: No Structures to Write

Complementary Sequence 2:
G*T*G*TGTGTGTGTGTGTGTTGGTGTTGTTGTTGTTGTTGTTGTTTTATGGTGTGTGTGTGTGTG*T*G*T

Secondary Structure Prediction Website: Delta G = **+5.9** (unlikely to create any secondary structures)

Melting Temperature Prediction Website: GC content = 43 % ; T_M = **73 ºC**
IDT OligoAnalyzer Tool Website: Maximum Delta G (*i.e.* free energy of the oligo sequence binding to its perfect complement) = **-118.35 kcal/mole**

Sequence 3: **Phosphorothioate-capped 45-BP dsDNA**
A*C*A*CACACACACACACACACACACA**AACAACAACAACAACAA*C*A*A**

Secondary Structure Prediction Website: No Structures to Write

Complementary Sequence 3:
T*T*G*TTGTTGTTGTTGTTTGTGTGTGTGTGTGTGTGTG*T*G*T

Secondary Structure Prediction Website: Delta G = **+3.8** (unlikely to create any secondary structures)

Melting Temperature Prediction Website: GC content = 40 % ; T_M = **66 ºC**
IDT OligoAnalyzer Tool Website: Maximum Delta G (*i.e.* free energy of the oligo sequence binding to its perfect complement) = **-74.88 kcal/mole**

Sequence 4: **Phosphorothioate-capped 20-BP dsDNA**
A*C*A*CACACACACACA*C*A*C

Secondary Structure Prediction Website: No Structures to Write

Complementary Sequence 4:
G*T*G*TGTGTGTGTGTG*T*G*T

Secondary Structure Prediction Website: Delta G = **+1.6** (unlikely to create any secondary structures)

Melting Temperature Prediction Website: GC content = 50 % ; T_M = **52 ºC**
IDT OligoAnalyzer Tool Website: Maximum Delta G (*i.e.* free energy of the oligo sequence binding to its perfect complement) = **-31 kcal/mole**

Supplementary Figure 1: The starting ISD library of synthetic dsDNA from IDT.
The star symbol (*i.e.* *) represents a phosphorothioate bond. Theoretical melting temperature values were obtained from the melting temperature prediction website, sciencelauncher.com/oligocalc.html. Theoretical maximum Delta G values were obtained from the IDT OligoAnalyzer Tool website, idtdna.com/calc/analyzer. Theoretical Delta G values for secondary structure formation were obtained from the secondary structure prediction website, rna.urmc.rochester.edu/RNAstructureWeb/Servers/Predict1/Predict1.html.

A

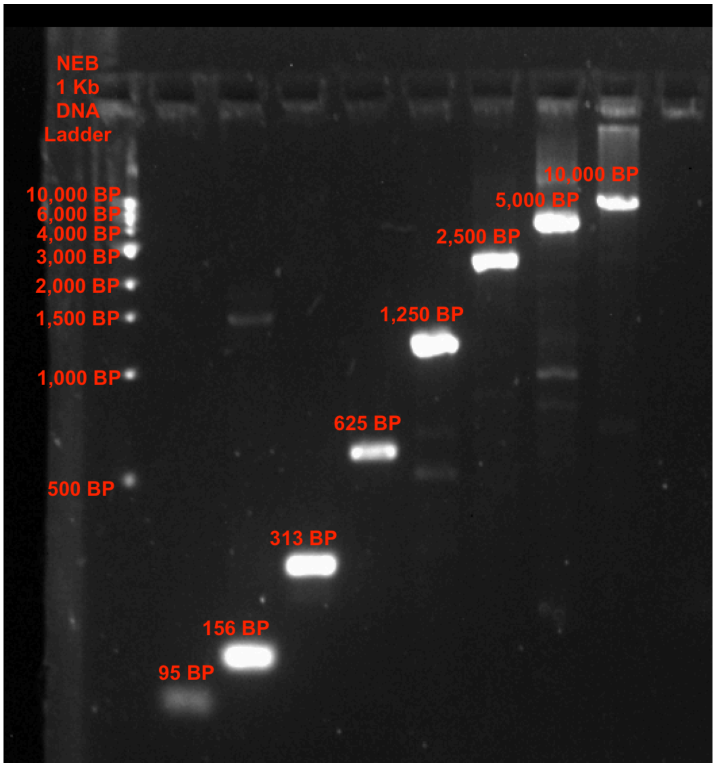

B

95BP TOP: TGATAACACTGCGGCCAACTTACTTCTGACAACGATCGGAGGACCGAAGGAGCTAACCGCTTTTTTGCACAACATGGGGGATCATGTAACTCGCC

95BP BOT: GGCGAGTTACATGATCCCCCATGTTGTGCAAAAAAGCGGTTAGCTCCTTCGGTCCTCCGATCGTTGTCAGAAGTAAGTTGGCCGCAGTGTTATCA

156BP TOP:
TGATAACACTGCGGCCAACTTACTTCTGACAACGATCGGAGGACCGAAGGAGCTAACCGCTTTTTTGCACAACATGGGGGATCATGTAACTCGCCTTGATCGTTG
GGAACCGGAGCTGAATGAAGCCATACCAAACGACGAGCGTGACACCACGAT

156BP BOT:
ATCGTGGTGTCACGCTCGTCGTTTGGTATGGCTTCATTCAGCTCCGGTTCCCAACGATCAAGGCGAGTTACATGATCCCCCATGTTGTGCAAAAAAGCGGTTAGCT
CCTTCGGTCCTCCGATCGTTGTCAGAAGTAAGTTGGCCGCAGTGTTATCA

313BP TOP:
TGATAACACTGCGGCCAACTTACTTCTGACAACGATCGGAGGACCGAAGGAGCTAACCGCTTTTTTGCACAACATGGGGGATCATGTAACTCGCCTTGATCGTTG
GGAACCGGAGCTGAATGAAGCCATACCAAACGACGAGCGTGACACCACGATGCCTGTAGCAATGGCAACAACGTTGCGCAAACTATTAACTGGCGAACTACTTA
CTCTAGCTTCCCGGCAACAATTAATAGACTGGATGGAGGCGGATAAAGTTGCAGGACCACTTCTGCGCTCGGCCCTTCCGGCTGGCTGGTTTATTGCTGATAAA

313BP BOT:
TTTATCAGCAATAAACCAGCCAGCCGGAAGGGCCGAGCGCAGAAGTGGTCCTGCAACTTTATCCGCCTCCATCCAGTCTATTAATTGTTGCCGGGAAGCTAGAGT
AAGTAGTTCGCCAGTTAATAGTTTGCGCAACGTTGTTGCCATTGCTACAGGCATCGTGGTGTCACGCTCGTCGTTTGGTATGGCTTCATTCAGCTCCGGTTCCCAAC
GATCAAGGCGAGTTACATGATCCCCCATGTTGTGCAAAAAAGCGGTTAGCTCCTTCGGTCCTCCGATCGTTGTCAGAAGTAAGTTGGCCGCAGTGTTATCA

625BP TOP:
TGATAACACTGCGGCCAACTTACTTCTGACAACGATCGGAGGACCGAAGGAGCTAACCGCTTTTTTGCACAACATGGGGGATCATGTAACTCGCCTTGATCGTTG
GGAACCGGAGCTGAATGAAGCCATACCAAACGACGAGCGTGACACCACGATGCCTGTAGCAATGGCAACAACGTTGCGCAAACTATTAACTGGCGAACTACTTA
CTCTAGCTTCCCGGCAACAATTAATAGACTGGATGGAGGCGGATAAAGTTGCAGGACCACTTCTGCGCTCGGCCCTTCCGGCTGGCTGGTTTATTGCTGATAAATC
TGGAGCCGGTGAGCGTGGGTCTCGCGGTATCATTGCAGCACTGGGGCCAGATGGTAAGCCCTCCCGTATCGTAGTTATCTACACGACGGGGAGTCAGGCAACTAT
GGATGAACGAAATAGACAGATCGCTGAGATAGGTGCCTCACTGATTAAGCATTGGTAACTGTCAGACCAAGTTTACTCATATATACTTTAGATTGATTTAAAACT
TCATTTTTAATTTAAAAGGATCTAGGTGAAGATCCTTTTTGATAATCTCATGACCAAAATCCCTTAACGTGAGTTTTCGTTCCACTGAGCGTCAGACCCC

625BP BOT:
GGGGTCTGACGCTCAGTGGAACGAAAACTCACGTTAAGGGATTTTGGTCATGAGATTATCAAAAAGGATCTTCACCTAGATCCTTTTAAATTAAAAATGAAGTTT
TAAATCAATCTAAAGTATATATGAGTAAACTTGGTCTGACAGTTACCAATGCTTAATCAGTGAGGCACCTATCTCAGCGATCTGTCTATTTCGTTCATCCATAGTT
GCCTGACTCCCCGTCGTGTAGATAACTACGATACGGGAGGGCTTACCATCTGGCCCCAGTGCTGCAATGATACCGCGAGACCCACGCTCACCGGCTCCAGATTTA

TCAGCAATAAACCAGCCAGCCGGAAGGGCCGAGCGCAGAAGTGGTCCTGCAACTTTATCCGCCTCCATCCAGTCTATTAATTGTTGCCGGGAAGCTAGAGTAAGT
AGTTCGCCAGTTAATAGTTTGCGCAACGTTGTTGCCATTGCTACAGGCATCGTGGTGTCACGCTCGTCGTTTGGTATGGCTTCATTCAGCTCCGGTTCCCAACGATC
AAGGCGAGTTACATGATCCCCCATGTTGTGCAAAAAAGCGGTTAGCTCCTTCGGTCCTCCGATCGTTGTCAGAAGTAAGTTGGCCGCAGTGTTATCA

1250BP TOP:
TGATAACACTGCGGCCAACTTACTTCTGACAACGATCGGAGGACCGAAGGAGCTAACCGCTTTTTTGCACAACATGGGGGATCATGTAACTCGCCTTGATCGTTG
GGAACCGGAGCTGAATGAAGCCATACCAAACGACGAGCGTGACACCACGATGCCTGTAGCAATGGCAACAACGTTGCGCAAACTATTAACTGGCGAACTACTTA
CTCTAGCTTCCCGGCAACAATTAATAGACTGGATGGAGGCGGATAAAGTTGCAGGACCACTTCTGCGCTCGGCCCTTCCGGCTGGCTGGTTTATTGCTGATAAATC
TGGAGCCGGTGAGCGTGGGTCTCGCGGTATCATTGCAGCACTGGGGCCAGATGGTAAGCCCTCCCGTATCGTAGTTATCTACACGACGGGGAGTCAGGCAACTAT
GGATGAACGAAATAGACAGATCGCTGAGATAGGTGCCTCACTGATTAAGCATTGGTAACTGTCAGACCAAGTTTACTCATATATACTTTAGATTGATTTAAAACT
TCATTTTTAATTTAAAAGGATCTAGGTGAAGATCCTTTTTGATAATCTCATGACCAAAATCCCTTAACGTGAGTTTTCGTTCCACTGAGCGTCAGACCCCGTAGAA
AAGATCAAAGGATCTTCTTGAGATCCTTTTTTTCTGCGCGTAATCTGCTGCTTGCAAACAAAAAAACCACCGCTACCAGCGGTGGTTTGTTTGCCGGATCAAGAGC
TACCAACTCTTTTTCCGAAGGTAACTGGCTTCAGCAGAGCGCAGATACCAAATACTGTTCTTCTAGTGTAGCCGTAGTTAGGCCACCACTTCAAGAACTCTGTAGC
ACCGCCTACATACCTCGCTCTGCTAATCCTGTTACCAGTGGCTGCTGCCAGTGGCGATAAGTCGTGTCTTACCGGGTTGGACTCAAGACGATAGTTACCGGATAAG
GCGCAGCGGTCGGGCTGAACGGGGGGTTCGTGCACACAGCCCAGCTTGGAGCGAACGACCTACACCGAACTGAGATACCTACAGCGTGAGCTATGAGAAAGCGC
CACGCTTCCCGAAGGGAGAAAGGCGGACAGGTATCCGGTAAGCGGCAGGGTCGGAACAGGAGAGCGCACGAGGGAGCTTCCAGGGGGAAACGCCTGGTATCTT
TATAGTCCTGTCGGGTTTCGCCACCTCTGACTTGAGCGTCGATTTTTGTGATGCTCGTCAGGGGGGCGGAGCCTATGGAAAAACGCCAGCAACG

1250BP BOT:
CGTTGCTGGCGTTTTTCCATAGGCTCCGCCCCCCTGACGAGCATCACAAAAATCGACGCTCAAGTCAGAGGTGGCGAAACCCGACAGGACTATAAAGA
TACCAGGCGTTTCCCCCTGGAAGCTCCCTCGTGCGCTCTCCTGTTCCGACCCTGCCGCTTACCGGATACCTGTCCGCCTTTCTCCCTTCGGGAAGCGTGGCGCTTTC
TCATAGCTCACGCTGTAGGTATCTCAGTTCGGTGTAGGTCGTTCGCTCCAAGCTGGGCTGTGTGCACGAACCCCCCGTTCAGCCCGACCGCTGCGCCTTATCCGGT
AACTATCGTCTTGAGTCCAACCCGGTAAGACACGACTTATCGCCACTGGCAGCAGCCACTGGTAACAGGATTAGCAGAGCGAGGTATGTAGGCGGTGCTACAGA
GTTCTTGAAGTGGTGGCCTAACTACGGCTACACTAGAAGAACAGTATTTGGTATCTGCGCTCTGCTGAAGCCAGTTACCTTCGGAAAAAGAGTTGGTAGCTCTTG
ATCCGGCAAACAAACCACCGCTGGTAGCGGTGGTTTTTTTGTTTGCAAGCAGCAGATTACGCGCAGAAAAAAAGGATCTCAAGAAGATCCTTTGATCTTTTCTAC
GGGGTCTGACGCTCAGTGGAACGAAAACTCACGTTAAGGGATTTTGGTCATGAGATTATCAAAAAGGATCTTCACCTAGATCCTTTTAAATTAAAAATGAAGTTT
TAAATCAATCTAAAGTATATATGAGTAAACTTGGTCTGACAGTTACCAATGCTTAATCAGTGAGGCACCTATCTCAGCGATCTGTCTATTTCGTTCATCCATAGTT
GCCTGACTCCCCGTCGTGTAGATAACTACGATACGGGAGGGCTTACCATCTGGCCCCAGTGCTGCAATGATACCGCGAGACCCACGCTCACCGGCTCCAGATTTA
TCAGCAATAAACCAGCCAGCCGGAAGGGCCGAGCGCAGAAGTGGTCCTGCAACTTTATCCGCCTCCATCCAGTCTATTAATTGTTGCCGGGAAGCTAGAGTAAGT
AGTTCGCCAGTTAATAGTTTGCGCAACGTTGTTGCCATTGCTACAGGCATCGTGGTGTCACGCTCGTCGTTTGGTATGGCTTCATTCAGCTCCGGTTCCCAACGATC
AAGGCGAGTTACATGATCCCCCATGTTGTGCAAAAAAGCGGTTAGCTCCTTCGGTCCTCCGATCGTTGTCAGAAGTAAGTTGGCCGCAGTGTTATCA

2500BP TOP:
AGGGACAGCAGAGATCCACTTTGGCGCCGGCTCGAGGGGGCCCGGGTGCAAAGATGGATAAAGTTTTAAACAGAGAGGAATCTTTGCAGCTAATGGACCTTCTA
GGTCTTGAAAGGAGTGGGAATTGGCTCCGGTGCCCGTCAGTGGGCAGAGCGCACATCGCCCACAGTCCCCGAGAAGTTGGGGGGAGGGGTCGGCAATTGATCCG
GTGCCTAGAGAAGGTGGCGCGGGGTAAACTGGGAAAGTGATGTCGTGTACTGGCTCCGCCTTTTTCCCGAGGGTGGGGGAGAACCGTATATAAGTGCAGTAGTC
GCCGTGAACGTTCTTTTTCGCAACGGGTTTGCCGCCAGAACACAGGTAAGTGCCGTGTGTGGTTCCCGCGGGCCTGGCCTCTTTACGGGTTATGGCCCTTGCGTGC
CTTGAATTACTTCCACCTGGCTGCAGTACGTGATTCTTGATCCCGAGCTTCGGGTTGGAAGTGGGTGGGAGAGTTCGAGGCCTTGCGCTTAAGGAGCCCCTTCGCC
TCGTGCTTGAGTTGAGGCCTGGCCTGGGCGCTGGGGCCGCCGCGTGCGAATCTGGTGGCACCTTCGCGCCTGTCTCGCTGCTTTCGATAAGTCTCTAGCCATTTAA
AATTTTTGATGACCTGCTGCGACGCTTTTTTTCTGGCAAGATAGTCTTGTAAATGCGGGCCAAGATCTGCACACTGGTATTTCGGTTTTTGGGGCCGCGGGCGGCG
ACGGGGCCCGTGCGTCCCAGCGCACATGTTCGGCGAGGCGGGGCCTGCGAGCGCGGCCACCGAGAATCGGACGGGGGTAGTCTCAAGCTGGCCGGCCTGCTCTG
GTGCCTGGCCTCGCGCCGCCGTGTATCGCCCCGCCCTGGGCGGCAAGGCTGGCCCGGTCGGCACCAGTTGCGTGAGCGGAAAGATGGCCGCTTCCCGGCCCTGCT
GCAGGGAGCTCAAAATGGAGGACGCGGCGCTCGGGAGAGCGGGCGGGTGAGTCACCCACACAAAGGAAAAGGGCCTTTCCGTCCTCAGCCGTCGCTTCATGTGA
CTCCACGGAGTACCGGGCGCCGTCCAGGCACCTCGATTAGTTCTCGAGCTTTTGGAGTACGTCGTCTTTAGGTTGGGGGGAGGGGTTTTATGCGATGGAGTTTCCC
CACACTGAGTGGGTGGAGACTGAAGTTAGGCCAGCTTGGCACTTGATGTAATTCTCCTTGGAATTTGCCCTTTTTGAGTTTGGATCTTGGTTCATTCTCAAGCCTCA
GACAGTGGTTCAAAGTTTTTTTCTTCCATTTCAGGTGTCGTGACGTACGGCCACCATGACCGAGTACAAGCCCACGGTGCGCCTCGCCACCCGCGACGACGTCCCC
AGGGCCGTACGCACCCTCGCCGCCGCGTTCGCCGACTACCCCGCCACGCGCCACACCGTCGATCCGGACCGCCACATCGAGCGGGTCACCGAGCTGCAAGAACT
CTTCCTCACGCGCGTCGGGCTCGACATCGGCAAGGTGTGGGTCGCGGACGACGGCGCCGCCGTGGCGGTCTGGACCACGCCGGAGAGCGTCGAAGCGGGGGCGG
TGTTCGCCGAGATCGGCCCGCGCATGGCCGAGTTGAGCGGTTCCCGGCTGGCCGCGCAGCAACAGATGGAAGGCCTCCTGGCGCCGCACCGGCCCAAGGAGCCC
GCGTGGTTCCTGGCCACCGTCGGAGTCTCGCCCGACCACCAGGGCAAGGGTCTGGGCAGCGCCGTCGTGCTCCCCGGAGTGGAGGCGGCCGAGCGCGCCGGGGT
GCCCGCCTTCCTGGAGACCTCCGCGCCCCGCAACCTCCCCTTCTACGAGCGGCTCGGCTTCACCGTCACCGCCGACGTCGAGGTGCCCGAAGGACCGCGCACCTG
GTGCATGACCCGCAAGCCCGGTGCCTGAACGCGTTAAGTCGACAATCAACCTCTGGATTACAAAATTTGTGAAAGATTGACTGGTATTCTTAACTATGTTGCTCCT
TTTACGCTATGTGGATACGCTGCTTTAATGCCTTTGTATCATGCTATTGCTTCCCGTATGGCTTTCATTTTCTCCTCCTTGTATAAATCCTGGTTGCTGTCTCTTTATG
AGGAGTTGTGGCCCGTTGTCAGGCAACGTGGCGTGGTGTGCACTGTGTTTGCTGACGCAACCCCCACTGGTTGGGGCATTGCCACCACCTGTCAGCTCCTTTCCGG
GACTTTCGCTTTCCCCCTCCCTATTGCCACGGCGGAACTCATCGCCGCCTGCCTTGCCCGCTGCTGGACAGGGGCTCGGCTGTTGGGCACTGACAATTCCGTGGTG
TTGTCGGGGAAATCATCGTCCTTTCCTTGGCTGCTCGCCTGTGTTGCCACCTGGATTCTGCGCGGGACGTCCTTCTGCTACGTCCCTTCGGCCCTCAATCCAGCGGA
CCTTCCTTCCCGCGGCCTGCTGCCGGCTCTGCGGCCTCTTCCGCGTCTTCGCCTTCGCCCTCAGACGAGTCGGATCT

2500BP BOT:
AGATCCGACTCGTCTGAGGGCGAAGGCGAAGACGCGGAAGAGGCCGCAGAGCCGGCAGCAGGCCGCGGGAAGGAAGGTCCGCTGGATTGAGGGCCGAAGGGA
CGTAGCAGAAGGACGTCCCGCGCAGAATCCAGGTGGCAACACAGGCGAGCAGCCAAGGAAAGGACGATGATTTCCCCGACAACACCACGGAATTGTCAGTGCC
CAACAGCCGAGCCCCTGTCCAGCAGCGGGCAAGGCAGGCGGCGATGAGTTCCGCCGTGGCAATAGGGAGGGGGAAAGCGAAAGTCCCGGAAAGGAGCTGACAG
GTGGTGGCAATGCCCCAACCAGTGGGGGTTGCGTCAGCAAACACAGTGCACACCACGCCACGTTGCCTGACAACGGGCCACAACTCCTCATAAAGAGACAGCAA
CCAGGATTTATACAAGGAGGAGAAAATGAAAGCCATACGGGAAGCAATAGCATGATACAAAGGCATTAAAGCAGCGTATCCACATAGCGTAAAAGGAGCAACA
TAGTTAAGAATACCAGTCAATCTTTCACAAATTTTGTAATCCAGAGGTTGATTGTCGACTTAACGCGTTCAGGCACCGGGCTTGCGGGTCATGCACCAGGTGCGCG
GTCCTTCGGGCACCTCGACGTCGGCGGTGACGGTGAAGCCGAGCCGCTCGTAGAAGGGGAGGTTGCGGGGCGCGGAGGTCTCCAGGAAGGCGGGCACCCCGGC
GCGCTCGGCCGCCTCCACTCCGGGGAGCACGACGGCGCTGCCCAGACCCTTGCCCTGGTGGTCGGGCGAGACTCCGACGGTGGCCAGGAACCACGCGGGCTCCT
TGGGCCGGTGCGGCGCCAGGAGGCCTTCCATCTGTTGCTGCGCGGCCAGCCGGGAACCGCTCAACTCGGCCATGCGCGGGCCGATCTCGGCGAACACCGCCCCC
GCTTCGACGCTCTCCGGCGTGGTCCAGACCGCCACGGCGGCGCCGTCGTCCGCGACCCACACCTTGCCGATGTCGAGCCCGACGCGCGTGAGGAAGAGTTCTTGC
AGCTCGGTGACCCGCTCGATGTGGCGGTCCGGATCGACGGTGTGGCGCGTGGCGGGGTAGTCGGCGAACGCGGCGGCGAGGGTGCGTACGGCCCTGGGGACGTC
GTCGCGGGTGGCGAGGCGCACCGTGGGCTTGTACTCGGTCATGGTGGCCGTACGTCACGACACCTGAAATGGAAGAAAAAAACTTTGAACCACTGTCTGAGGCTT
GAGAATGAACCAAGATCCAAACTCAAAAAGGGCAAATTCCAAGGAGAATTACATCAAGTGCCAAGCTGGCCTAACTTCAGTCTCCACCCACTCAGTGTGGGGAA
ACTCCATCGCATAAAACCCCTCCCCCCAACCTAAAGACGACGTACTCCAAAAGCTCGAGAACTAATCGAGGTGCCTGGACGGCGCCCGGTACTCCGTGGAGTCAC
ATGAAGCGACGGCTGAGGACGGAAAGGCCCTTTTCCTTTGTGTGGGTGACTCACCCGCCCGCTCTCCCGAGCGCCGCGTCCTCCATTTTGAGCTCCCTGCAGCAG
GGCCGGGAAGCGGCCATCTTTCCGCTCACGCAACTGGTGCCGACCGGGCCAGCCTTGCCGCCCAGGGCGGGGCGATACACGGCGGCGCGAGGCCAGGCACCAGA
GCAGGCCGGCCAGCTTGAGACTACCCCCGTCCGATTCTCGGTGGCCGCGCTCGCAGGCCCCGCCTCGCCGAACATGTGCGCTGGGACGCACGGGCCCCGTCGCCG
CCCGCGGCCCCAAAAACCGAAATACCAGTGTGCAGATCTTGGCCCGCATTTACAAGACTATCTTGCCAGAAAAAAAGCGTCGCAGCAGGTCATCAAAAATTTTA
AATGGCTAGAGACTTATCGAAAGCAGCGAGACAGGCGCGAAGGTGCCACCAGATTCGCACGCGGCGGCCCCAGCGCCCAGGCCAGGCCTCAACTCAAGCACGA

GGCGAAGGGGCTCCTTAAGCGCAAGGCCTCGAACTCTCCCACCCACTTCCAACCCGAAGCTCGGGATCAAGAATCACGTACTGCAGCCAGGTGGAAGTAATTCA
AGGCACGCAAGGGCCATAACCCGTAAAGAGGCCAGGCCCGCGGGAACCACACACGGCACTTACCTGTGTTCTGGCGGCAAACCCGTTGCGAAAAAGAACGTTCA
CGGCGACTACTGCACTTATATACGGTTCTCCCCCACCCTCGGGAAAAAGGCGGAGCCAGTACACGACATCACTTTCCCAGTTTACCCCGCGCCACCTTCTCTAGGC
ACCGGATCAATTGCCGACCCCTCCCCCCAACTTCTCGGGGACTGTGGGCGATGTGCGCTCTGCCCACTGACGGGCACCGGAGCCAATTCCCACTCCTTTCAAGAC
CTAGAAGGTCCATTAGCTGCAAAGATTCCTCTCTGTTTAAAACTTTATCCATCTTTGCACCCGGGCCCCCTCGAGCCGGCGCCAAAGTGGATCTCTGCTGTCCCT

5000BP TOP:
ATCCGGTGCCTAGAGAAGGTGGCGCGGGGTAAACTGGGAAAGTGATGTCGTGTACTGGCTCCGCCTTTTTCCCGAGGGTGGGGGAGAACCGTATATAAGTGCAG
TAGTCGCCGTGAACGTTCTTTTTCGCAACGGGTTTGCCGCCAGAACACAGGTAAGTGCCGTGTGTGGTTCCCGCGGGCCTGGCCTCTTTACGGGTTATGGCCCTTG
CGTGCCTTGAATTACTTCCACCTGGCTGCAGTACGTGATTCTTGATCCCGAGCTTCGGGTTGGAAGTGGGTGGGAGAGTTCGAGGCCTTGCGCTTAAGGAGCCCCT
TCGCCTCGTGCTTGAGTTGAGGCCTGGCCTGGGCGCTGGGGCCGCCGCGTGCGAATCTGGTGGCACCTTCGCGCCTGTCTCGCTGCTTTCGATAAGTCTCTAGCCA
TTTAAAATTTTTGATGACCTGCTGCGACGCTTTTTTTCTGGCAAGATAGTCTTGTAAATGCGGGCCAAGATCTGCACACTGGTATTTCGGTTTTTGGGGCCGCGGGC
GGCGACGGGGCCCGTGCGTCCCAGCGCACATGTTCGGCGAGGCGGGGCCTGCGAGCGCGGCCACCGAGAATCGGACGGGGGTAGTCTCAAGCTGGCCGGCCTGC
TCTGGTGCCTGGCCTCGCGCCGCCGTGTATCGCCCCGCCCTGGGCGGCAAGGCTGGCCCGGTCGGCACCAGTTGCGTGAGCGGAAAGATGGCCGCTTCCCGGCCC
TGCTGCAGGGAGCTCAAAATGGAGGACGCGGCGCTCGGGAGAGCGGGCGGGTGAGTCACCCACACAAAGGAAAAGGGCCTTTCCGTCCTCAGCCGTCGCTTCAT
GTGACTCCACGGAGTACCGGGCGCCGTCCAGGCACCTCGATTAGTTCTCGAGCTTTTGGAGTACGTCGTCTTTAGGTTGGGGGGAGGGGTTTTATGCGATGGAGTT
TCCCCACACTGAGTGGGTGGAGACTGAAGTTAGGCCAGCTTGGCACTTGATGTAATTCTCCTTGGAATTTGCCCTTTTTGAGTTTGGATCTTGGTTCATTCTCAAGC
CTCAGACAGTGGTTCAAAGTTTTTTTCTTCCATTTCAGGTGTCGTGACGTACGGCCACCATGACCGAGTACAAGCCCACGGTGCGCCTCGCCACCCGCGACGACGT
CCCCAGGGCCGTACGCACCCTCGCCGCCGCGTTCGCCGACTACCCCGCCACGCGCCACACCGTCGATCCGGACCGCCACATCGAGCGGGTCACCGAGCTGCAAG
AACTCTTCCTCACGCGCGTCGGGCTCGACATCGGCAAGGTGTGGGTCGCGGACGACGGCGCCGCCGTGGCGGTCTGGACCACGCCGGAGAGCGTCGAAGCGGGG
GCGGTGTTCGCCGAGATCGGCCCGCGCATGGCCGAGTTGAGCGGTTCCCGGCTGGCCGCGCAGCAACAGATGGAAGGCCTCCTGGCGCCGCACCGGCCCAAGGA
GCCCGCGTGGTTCCTGGCCACCGTCGGAGTCTCGCCCGACCACCAGGGCAAGGGTCTGGGCAGCGCCGTCGTGCTCCCCGGAGTGGAGGCGGCCGAGCGCGCCG
GGGTGCCCGCCTTCCTGGAGACCTCCGCGCCCCGCAACCTCCCCTTCTACGAGCGGCTCGGCTTCACCGTCACCGCCGACGTCGAGGTGCCCGAAGGACCGCGCA
CCTGGTGCATGACCCGCAAGCCCGGTGCCTGAACGCGTTAAGTCGACAATCAACCTCTGGATTACAAAATTTGTGAAAGATTGACTGGTATTCTTAACTATGTTGC
TCCTTTTACGCTATGTGGATACGCTGCTTTAATGCCTTTGTATCATGCTATTGCTTCCCGTATGGCTTTCATTTTCTCCTCCTTGTATAAATCCTGGTTGCTGTCTCTT
TATGAGGAGTTGTGGCCCGTTGTCAGGCAACGTGGCGTGGTGTGCACTGTGTTTGCTGACGCAACCCCCACTGGTTGGGGCATTGCCACCACCTGTCAGCTCCTTT
CCGGGACTTTCGCTTTCCCCCTCCCTATTGCCACGGCGGAACTCATCGCCGCCTGCCTTGCCCGCTGCTGGACAGGGGCTCGGCTGTTGGGCACTGACAATTCCGT
GGTGTTGTCGGGGAAATCATCGTCCTTTCCTTGGCTGCTCGCCTGTGTTGCCACCTGGATTCTGCGCGGGACGTCCTTCTGCTACGTCCCTTCGGCCCTCAATCCAG
CGGACCTTCCTTCCCGCGGCCTGCTGCCGGCTCTGCGGCCTCTTCCGCGTCTTCGCCTTCGCCCTCAGACGAGTCGGATCTCCCTTTGGGCCGCCTCCCCGCGTCGA
CTTTAAGACCAATGACTTACAAGGCAGCTGTAGATCTTAGCCACTTTTTAAAAGAAAAGGGGGGACTGGAAGGGCTAATTCACTCCCAACGAAGACAAGATCTG
CTTTTTGCTTGTACTGGGTCTCTCTGGTTAGACCAGATCTGAGCCTGGGAGCTCTCTGGCTAACTAGGGAACCCACTGCTTAAGCCTCAATAAAGCTTGCCTTGAG
TGCTTCAAGTAGTGTGTGCCCGTCTGTTGTGTGACTCTGGTAACTAGAGATCCCTCAGACCCTTTTAGTCAGTGTGGAAAATCTCTAGCAGTACGTATAGTAGTTC
ATGTCATCTTATTATTCAGTATTTATAACTTGCAAAGAAATGAATATCAGAGAGTGAGAGGAACTTGTTTATTGCAGCTTATAATGGTTACAAATAAAGCAATAGC
ATCACAAATTTCACAAATAAAGCATTTTTTTCACTGCATTCTAGTTGTGGTTTGTCCAAACTCATCAATGTATCTTATCATGTCTGGCTCTAGCTATCCCGCCCCTA
ACTCCGCCCATCCCGCCCCTAACTCCGCCCAGTTCCGCCCATTCTCCGCCCCATGGCTGACTAATTTTTTTTATTTATGCAGAGGCCGAGGCCGCCTCGGCCTCTGA
GCTATTCCAGAAGTAGTGAGGAGGCTTTTTTGGAGGCCTAGGGACGTACCCAATTCGCCCTATAGTGAGTCGTATTACGCGCGCTCACTGGCCGTCGTTTTACAAC
GTCGTGACTGGGAAAACCCTGGCGTTACCCAACTTAATCGCCTTGCAGCACATCCCCCTTTCGCCAGCTGGCGTAATAGCGAAGAGGCCCGCACCGATCGCCCTT
CCCAACAGTTGCGCAGCCTGAATGGCGAATGGGACGCGCCCTGTAGCGGCGCATTAAGCGCGGCGGGTGTGGTGGTTACGCGCAGCGTGACCGCTACACTTGCC
AGCGCCCTAGCGCCCGCTCCTTTCGCTTTCTTCCCTTCCTTTCTCGCCACGTTCGCCGGCTTTCCCCGTCAAGCTCTAAATCGGGGGCTCCCTTTAGGGTTCCGATTT
AGTGCTTTACGGCACCTCGACCCCAAAAAACTTGATTAGGGTGATGGTTCACGTAGTGGGCCATCGCCCTGATAGACGGTTTTTCGCCCTTTGACGTTGGAGTCCA
CGTTCTTTAATAGTGGACTCTTGTTCCAAACTGGAACAACACTCAACCCTATCTCGGTCTATTCTTTTGATTTATAAGGGATTTTGCCGATTTCGGCCTATTGGTTA
AAAAATGAGCTGATTTAACAAAAATTTAACGCGAATTTTAACAAAATATTAACGCTTACAATTTAGGTGGCACTTTTCGGGGAAATGTGCGCGGAACCCCTATTT
GTTTATTTTTCTAAATACATTCAAATATGTATCCGCTCATGAGACAATAACCCTGATAAATGCTTCAATAATATTGAAAAAGGAAGAGTATGAGTATTCAACATTT
CCGTGTCGCCCTTATTCCCTTTTTTGCGGCATTTTGCCTTCCTGTTTTTGCTCACCCAGAAACGCTGGTGAAAGTAAAAGATGCTGAAGATCAGTTGGGTGCACGA
GTGGGTTACATCGAACTGGATCTCAACAGCGGTAAGATCCTTGAGAGTTTTCGCCCCGAAGAACGTTTTCCAATGATGAGCACTTTTAAAGTTCTGCTATGTGGCG
CGGTATTATCCCGTATTGACGCCGGGCAAGAGCAACTCGGTCGCCGCATACACTATTCTCAGAATGACTTGGTTGAGTACTCACCAGTCACAGAAAAGCATCTTA
CGGATGGCATGACAGTAAGAGAATTATGCAGTGCTGCCATAACCATGAGTGATAACACTGCGGCCAACTTACTTCTGACAACGATCGGAGGACCGAAGGAGCTA
ACCGCTTTTTTGCACAACATGGGGGATCATGTAACTCGCCTTGATCGTTGGGAACCGGAGCTGAATGAAGCCATACCAAACGACGAGCGTGACACCACGATGCCT
GTAGCAATGGCAACAACGTTGCGCAAACTATTAACTGGCGAACTACTTACTCTAGCTTCCCGGCAACAATTAATAGACTGGATGGAGGCGGATAAAGTTGCAGG
ACCACTTCTGCGCTCGGCCCTTCCGGCTGGCTGGTTTATTGCTGATAAATCTGGAGCCGGTGAGCGTGGGTCTCGCGGTATCATTGCAGCACTGGGGCCAGATGGT
AAGCCCTCCCGTATCGTAGTTATCTACACGACGGGGAGTCAGGCAACTATGGATGAACGAAATAGACAGATCGCTGAGATAGGTGCCTCACTGATTAAGCATTGG
TAACTGTCAGACCAAGTTTACTCATATATACTTTAGATTGATTTAAAACTTCATTTTTAATTTAAAAGGATCTAGGTGAAGATCCTTTTTGATAATCTCATGACCAA
AATCCCTTAACGTGAGTTTTCGTTCCACTGAGCGTCAGACCCCGTAGAAAAGATCAAAGGATCTTCTTGAGATCCTTTTTTTCTGCGCGTAATCTGCTGCTTGCAA
ACAAAAAAACCACCGCTACCAGCGGTGGTTTGTTTGCCGGATCAAGAGCTACCAACTCTTTTTCCGAAGGTAACTGGCTTCAGCAGAGCGCAGATACCAAATACT
GTTCTTCTAGTGTAGCCGTAGTTAGGCCACCACTT

5000BP BOT:
AAGTGGTGGCCTAACTACGGCTACACTAGAAGAACAGTATTTGGTATCTGCGCTCTGCTGAAGCCAGTTACCTTCGGAAAAAGAGTTGGTAGCTCTTGATCCGGC
AAACAAACCACCGCTGGTAGCGGTGGTTTTTTTGTTTGCAAGCAGCAGATTACGCGCAGAAAAAAAGGATCTCAAGAAGATCCTTTGATCTTTTCTACGGGGTCT
GACGCTCAGTGGAACGAAAACTCACGTTAAGGGATTTTGGTCATGAGATTATCAAAAAGGATCTTCACCTAGATCCTTTTAAATTAAAAATGAAGTTTTAAATCA
ATCTAAAGTATATATGAGTAAACTTGGTCTGACAGTTACCAATGCTTAATCAGTGAGGCACCTATCTCAGCGATCTGTCTATTTCGTTCATCCATAGTTGCCTGACT
CCCCGTCGTGTAGATAACTACGATACGGGAGGGCTTACCATCTGGCCCCAGTGCTGCAATGATACCGCGAGACCCACGCTCACCGGCTCCAGATTTATCAGCAAT
AAACCAGCCAGCCGGAAGGGCCGAGCGCAGAAGTGGTCCTGCAACTTTATCCGCCTCCATCCAGTCTATTAATTGTTGCCGGGAAGCTAGAGTAAGTAGTTCGCC
AGTTAATAGTTTGCGCAACGTTGTTGCCATTGCTACAGGCATCGTGGTGTCACGCTCGTCGTTTGGTATGGCTTCATTCAGCTCCGGTTCCCAACGATCAAGGCGA
GTTACATGATCCCCCATGTTGTGCAAAAAAGCGGTTAGCTCCTTCGGTCCTCCGATCGTTGTCAGAAGTAAGTTGGCCGCAGTGTTATCACTCATGGTTATGGCAG
CACTGCATAATTCTCTTACTGTCATGCCATCCGTAAGATGCTTTTCTGTGACTGGTGAGTACTCAACCAAGTCATTCTGAGAATAGTGTATGCGGCGACCGAGTTG
CTCTTGCCCGGCGTCAATACGGGATAATACCGCGCCACATAGCAGAACTTTAAAAGTGCTCATCATTGGAAAACGTTCTTCGGGGCGAAAACTCTCAAGGATCTT
ACCGCTGTTGAGATCCAGTTCGATGTAACCCACTCGTGCACCCAACTGATCTTCAGCATCTTTTACTTTCACCAGCGTTTCTGGGTGAGCAAAAACAGGAAGGCAA
AATGCCGCAAAAAAGGGAATAAGGGCGACACGGAAATGTTGAATACTCATACTCTTCCTTTTTCAATATTATTGAAGCATTTATCAGGGTTATTGTCTCATGAGCG
GATACATATTTGAATGTATTTAGAAAAATAAACAAATAGGGGTTCCGCGCACATTTCCCCGAAAAGTGCCACCTAAATTGTAAGCGTTAATATTTTGTTAAAATTC
GCGTTAAATTTTTGTTAAATCAGCTCATTTTTTAACCAATAGGCCGAAATCGGCAAAATCCCTTATAAATCAAAAGAATAGACCGAGATAGGGTTGAGTGTTGTTC
CAGTTTGGAACAAGAGTCCACTATTAAAGAACGTGGACTCCAACGTCAAAGGGCGAAAAACCGTCTATCAGGGCGATGGCCCACTACGTGAACCATCACCCTAA
TCAAGTTTTTTGGGGTCGAGGTGCCGTAAAGCACTAAATCGGAACCCTAAAGGGAGCCCCCGATTTAGAGCTTGACGGGGAAAGCCGGCGAACGTGGCGAGAAA
GGAAGGGAAGAAAGCGAAAGGAGCGGGCGCTAGGGCGCTGGCAAGTGTAGCGGTCACGCTGCGCGTAACCACCACACCCGCCGCGCTTAATGCGCCGCTACAG
GGCGCGTCCCATTCGCCATTCAGGCTGCGCAACTGTTGGGAAGGGCGATCGGTGCGGGCCTCTTCGCTATTACGCCAGCTGGCGAAAGGGGGATGTGCTGCAAGG
CGATTAAGTTGGGTAACGCCAGGGTTTTCCCAGTCACGACGTTGTAAAACGACGGCCAGTGAGCGCGCGTAATACGACTCACTATAGGGCGAATTGGGTACGTCC
CTAGGCCTCCAAAAAAGCCTCCTCACTACTTCTGGAATAGCTCAGAGGCCGAGGCGGCCTCGGCCTCTGCATAAATAAAAAAAATTAGTCAGCCATGGGGCGGA
GAATGGGCGGAACTGGGCGGAGTTAGGGGCGGGATGGGCGGAGTTAGGGGCGGGATAGCTAGAGCCAGACATGATAAGATACATTGATGAGTTTGGACAAACC

ACAACTAGAATGCAGTGAAAAAAATGCTTTATTTGTGAAATTTGTGATGCTATTGCTTTATTTGTAACCATTATAAGCTGCAATAAACAAGTTCCTCTCACTCTCT
GATATTCATTTCTTTGCAAGTTATAAATACTGAATAATAAGATGACATGAACTACTATACGTACTGCTAGAGATTTTCCACACTGACTAAAAGGGTCTGAGGGATC
TCTAGTTACCAGAGTCACACAACAGACGGGCACACACTACTTGAAGCACTCAAGGCAAGCTTTATTGAGGCTTAAGCAGTGGGTTCCCTAGTTAGCCAGAGAGCT
CCCAGGCTCAGATCTGGTCTAACCAGAGAGACCCAGTACAAGCAAAAAGCAGATCTTGTCTTCGTTGGGAGTGAATTAGCCCTTCCAGTCCCCCCTTTTCTTTTAA
AAAGTGGCTAAGATCTACAGCTGCCTTGTAAGTCATTGGTCTTAAAGTCGACGCGGGGAGGCGGCCCAAAGGGAGATCCGACTCGTCTGAGGGCGAAGGCGAAG
ACGCGGAAGAGGCCGCAGAGCCGGCAGCAGGCCGCGGGAAGGAAGGTCCGCTGGATTGAGGGCCGAAGGGACGTAGCAGAAGGACGTCCCGCGCAGAATCCA
GGTGGCAACACAGGCGAGCAGCCAAGGAAAGGACGATGATTTCCCCGACAACACCACGGAATTGTCAGTGCCCAACAGCCGAGCCCCTGTCCAGCAGCGGGCA
AGGCAGGCGGCGATGAGTTCCGCCGTGGCAATAGGGAGGGGGAAAGCGAAAGTCCCGGAAAGGAGCTGACAGGTGGTGGCAATGCCCCAACCAGTGGGGGTTG
CGTCAGCAAACACAGTGCACACCACGCCACGTTGCCTGACAACGGGCCACAACTCCTCATAAAGAGACAGCAACCAGGATTTATACAAGGAGGAGAAAATGAA
AGCCATACGGGAAGCAATAGCATGATACAAAGGCATTAAAGCAGCGTATCCACATAGCGTAAAAGGAGCAACATAGTTAAGAATACCAGTCAATCTTTCACAAA
TTTTGTAATCCAGAGGTTGATTGTCGACTTAACGCGTTCAGGCACCGGGCTTGCGGGTCATGCACCAGGTGCGCGGTCCTTCGGGCACCTCGACGTCGGCGGTGA
CGGTGAAGCCGAGCCGCTCGTAGAAGGGGAGGTTGCGGGGCGCGGAGGTCTCCAGGAAGGCGGGCACCCCGGCGCGCTCGGCCGCCTCCACTCCGGGGAGCAC
GACGGCGCTGCCCAGACCCTTGCCCTGGTGGTCGGGCGAGACTCCGACGGTGGCCAGGAACCACGCGGGCTCCTTGGGCCGGTGCGGCGCCAGGAGGCCTTCCA
TCTGTTGCTGCGCGGCCAGCCGGGAACCGCTCAACTCGGCCATGCGCGGGCCGATCTCGGCGAACACCGCCCCCGCTTCGACGCTCTCCGGCGTGGTCCAGACCG
CCACGGCGGCGCCGTCGTCCGCGACCCACACCTTGCCGATGTCGAGCCCGACGCGCGTGAGGAAGAGTTCTTGCAGCTCGGTGACCCGCTCGATGTGGCGGTCCG
GATCGACGGTGTGGCGCGTGGCGGGGTAGTCGGCGAACGCGGCGGCGAGGGTGCGTACGGCCCTGGGGACGTCGTCGCGGGTGGCGAGGCGCACCGTGGGCTT
GTACTCGGTCATGGTGGCCGTACGTCACGACACCTGAAATGGAAGAAAAAAACTTTGAACCACTGTCTGAGGCTTGAGAATGAACCAAGATCCAAACTCAAAAA
GGGCAAATTCCAAGGAGAATTACATCAAGTGCCAAGCTGGCCTAACTTCAGTCTCCACCCACTCAGTGTGGGGAAACTCCATCGCATAAAACCCCTCCCCCCAAC
CTAAAGACGACGTACTCCAAAAGCTCGAGAACTAATCGAGGTGCCTGGACGGCGCCCGGTACTCCGTGGAGTCACATGAAGCGACGGCTGAGGACGGAAAGGC
CCTTTTCCTTTGTGTGGGTGACTCACCCGCCCGCTCTCCCGAGCGCCGCGTCCTCCATTTGAGCTCCCTGCAGCAGGGCCGGGAAGCGGCCATCTTTCCGCTCAC
GCAACTGGTGCCGACCGGGCCAGCCTTGCCGCCCAGGGCGGGGCGATACACGGCGGCGCGAGGCCAGGCACCAGAGCAGGCCGGCCAGCTTGAGACTACCCCC
GTCCGATTCTCGGTGGCCGCGCTCGCAGGCCCCGCCTCGCCGAACATGTGCGCTGGGACGCACGGGCCCCGTCGCCGCCCGCGGCCCCAAAAACCGAAATACCA
GTGTGCAGATCTTGGCCCGCATTTACAAGACTATCTTGCCAGAAAAAAAGCGTCGCAGCAGGTCATCAAAAATTTTAAATGGCTAGAGACTTATCGAAAGCAGCG
AGACAGGCGCGAAGGTGCCACCAGATTCGCACGCGGCGGCCCCAGCGCCCAGGCCAGGCCTCAACTCAAGCACGAGGCGAAGGGGCTCCTTAAGCGCAAGGCC
TCGAACTCTCCCACCCACTTCCAACCCGAAGCTCGGGATCAAGAATCACGTACTGCAGCCAGGTGGAAGTAATTCAAGGCACGCAAGGGCCATAACCCGTAAAG
AGGCCAGGCCCGCGGGAACCACACACGGCACTTACCTGTGTTCTGGCGGCAAACCCGTTGCGAAAAAGAACGTTCACGGCGACTACTGCACTTATATACGGTTCT
CCCCCACCCTCGGGAAAAAGGCGGAGCCAGTACACGACATCACTTTCCCAGTTTACCCCGCGCCACCTTCTCTAGGCACCGGAT

10000BP TOP:
ATCGTTTCAGACCCACCTCCCAACCCCGAGGGGACCCAGAGAGGGCCTATTTCCCATGATTCCTTCATATTTGCATATACGATACAAGGCTGTTAGAGAGATAATT
AGAATTAATTTGACTGTAAACACAAAGATATTAGTACAAAATACGTGACGTAGAAAGTAATAATTTCTTGGGTAGTTTGCAGTTTTAAAATTATGTTTTAAAATGG
ACTATCATATGCTTACCGTAACTTGAAAGTATTTCGATTTCTTGGCTTTATATATCTTGTGGAAAGGACGAAACACCGGAGACGGTTGTAAATGAGCACACAAAAT
ACACATGCTAAAATATTATATTCTATGACCTTTATAAAATCAACCAAAATCTTCTTTTTAATAACTTTAGTATCAATAATTAGAATTTTTATGTTCCTTTTTGCAAA
CTTTTAATAAAAATGAGCAAAATAAAAAAACGCTAGTTTTAGTAACTCGCGTTGTTTTCTTCACCTTTAATAATAGCTACTCCACCACTTGTTCCTAAGCGGTCAG
CTCCTGCTTCAATCATTTTTTGAGCATCTTCAAATGTTCTAACTCCACCAGCTGCTTTAACTAAAGCATTGTCTTTAACAACTGACTTCATTAGTTTAACATCTTCAA
ATGTTGCACCTGATTTTGAAAATCCTGTTGATGTTTTAACAAATTCTAATCCAGCTTCAACAGCTATTTCACAAGCTTTCATGATTTCTTCTTTTGTTAATAAACAAT
TTTCCATAATACATTTAACAACATGTGATCCAGCTGCTTTTTTTACAGCTTTCATGTCTTCTAAAACTAATTCATAATTTTTGTCTTTTAATGCACCAATATTTAATA
CCATATCAATTTCTGTTGCACCATCTTTAATTGCTTCAGAAACTTCGAATGCTTTTGTAGCTGTTGTGCATGCACCTAGAGGAAAACCTACAACATTTGTTATTCCT
ACATTTGTGCCTTTTAATAATTCTTTACAATAGCTTGTTCAATATGAATTAACACAAACTGTTGCAAAATCAAATTCAATTGCTTCATCACATAATTGTTTAATTTC
AGCTTTCGTAGCATCTTGTTTTAATAATGTGTGATCTATATATTTGTTTAGTTTCATTTTTTCTCCTATATATTCATTTTTAATTTTAATTCTTTAATAATTTCGTCTAC
TTTAACTTTAGCGTTTTGAACAGATTCACCAACACCTATAAAATAAATTTTTAGTTTAGGTTCAGTTCCACTTGGGCGAACAGCAAATCATGACTTATCTTCTAAAT
AAAATTTTAGTAAGTCTTGTCCTGGCATATTATACATTCCATCGATGTAGTCTTCAACATTAACAACTTTAAGTCCAGCAATTTGAGTTAAGGGTGTTGCTCTCAAT
GATTTCATTAATGGTTCAATTTTTAATTTCTTTTCTTCTGGTTTAAAATTCAAGTTTAAAGTGAAAGTGTAATATGCACCCATTTCTTTAAATAAATCTTCTAAATAG
TCTACTAATGTTTTATTTTGTTTTTTATAAAATCAAGCAGCCTCTGCTATTAATATAGAAGCTTGTATTCCATCTTTATCTCTAGCTGAGTCATCAATTACATATCCA
TAACTTTCTTCATAAGCAAAAACAAAATTTAATCCGTTATCTTCTTCTTTAGCAATTTCTCTACCCATTCATTTAAATCCAGTTAAAGTTTTTACAATATTAACTCCA
TATTTTTCATGAGCGATTCTATCACCCAAATCACTTGTTACAAAACTTGAATATAGAGCCGGATTTTTTGGAATGCTATTTAAGCGTTTTAGATTTGATAATTTTCA
ATCAATTAAAATTGGTCCTGTTTGATTTCCATCTAATCTTACAAAATGACCATCATGTTTTATTGCCATTCCAAATCTGTCAGCATCTGGGTCATTCATAATAATAA
TATCTGCATCATGTTTAATACCATATTCAAGCGGTATTTTTCATGCAGGATCAAATTCTGGATTTGGATTTACAACATTTTTAAATGTTTCATCTTCAAATGCATGC
TCTTCAACCTCAATAACGTTATATCCTGATTCACGTAATATTTTTGGGGTAAATTTAGTTCCTGTTCCATTAACTGCGCTAAAAATAATTTTAAATCTTTTTAGCT
TCTTGCTCTTTTTTGTACGTCTCTGTTTTAGAGCTAGAAATAGCAAGTTAAAATAAGGCTAGTCCGTTATCAACTTGAAAAAGTGGCACCGAGTCGGTGCTTTTTTA
AGCTTGGCGTAACTAGATCTTGAGACAAATGGCAGTATTCATCCACAATTTTAAAAGAAAAGGGGGGATTGGGGGGTACAGTGCAGGGGAAAGAATAGTAGACA
TAATAGCAACAGACATACAAACTAAAGAATTACAAAAACAAATTACAAAAATTCAAAATTTTCGGGTTTATTACAGGGACAGCAGAGATCCACTTTGGCGCCGG
CTCGAGGGGGCCCGGGTGCAAAGATGGATAAAGTTTTAAACAGAGAGGAATCTTTGCAGCTAATGGACCTTCTAGGTCTTGAAAGGAGTGGGAATTGGCTCCGG
TGCCCGTCAGTGGGCAGAGCGCACATCGCCCACAGTCCCCGAGAAGTTGGGGGGAGGGGTCGGCAATTGATCCGGTGCCTAGAGAAGGTGGCGCGGGGTAAACT
GGGAAAGTGATGTCGTGTACTGGCTCCGCCTTTTTCCCGAGGGTGGGGGAGAACCGTATATAAGTGCAGTAGTCGCCGTGAACGTTCTTTTTCGCAACGGGTTTGC
CGCCAGAACACAGGTAAGTGCCGTGTGTGGTTCCCGCGGGCCTGGCCTCTTTACGGGTTATGGCCCTTGCGTGCCTTGAATTACTTCCACCTGGCTGCAGTACGTG
ATTCTTGATCCCGAGCTTCGGGTTGGAAGTGGGTGGGAGAGTTCGAGGCCTTGCGCTTAAGGAGCCCCTTCGCCTCGTGCTTGAGTTGAGGCCTGGCCTGGGCGCT
GGGGCCGCCGCGTGCGAATCTGGTGGCACCTTCGCGCCTGTCTCGCTGCTTTCGATAAGTCTCTAGCCATTTAAAATTTTTGATGACCTGCTGCGACGCTTTTTTTC
TGGCAAGATAGTCTTGTAAATGCGGGCCAAGATCTGCACACTGGTATTTCGGTTTTTGGGGCCGCGGGCGGCGACGGGGCCCGTGCGTCCCAGCGCACATGTTCG
GCGAGGCGGGGCCTGCGAGCGCGGCCACCGAGAATCGGACGGGGGTAGTCTCAAGCTGGCCGGCCTGCTCTGGTGCCTGGCCTCGCGCCGCCGTGTATCGCCCC
GCCCTGGGCGGCAAGGCTGGCCCGGTCGGCACCAGTTGCGTGAGCGGAAAGATGGCCGCTTCCCGGCCCTGCTGCAGGGAGCTCAAAATGGAGGACGCGGCGCT
CGGGAGAGCGGGCGGGTGAGTCACCCACACAAAGGAAAAGGGCCTTTCCGTCCTCAGCCGTCGCTTCATGTGACTCCACGGAGTACCGGGCGCCGTCCAGGCAC
CTCGATTAGTTCTCGAGCTTTTGGAGTACGTCGTCTTTAGGTTGGGGGGAGGGGTTTTATGCGATGGAGTTTCCCCACACTGAGTGGGTGGAGACTGAAGTTAGGC
CAGCTTGGCACTTGATGTAATTCTCCTTGGAATTTGCCCTTTTTGAGTTTGGATCTTGGTTCATTCTCAAGCCTCAGACAGTGGTTCAAAGTTTTTTTCTTCCATTTC
AGGTGTCGTGACGTACGGCCACCATGACCGAGTACAAGCCCACGGTGCGCCTCGCCACCCGCGACGACGTCCCCAGGGCCGTACGCACCCTCGCCGCCGCGTTCG
CCGACTACCCCGCCACGCGCCACACCGTCGATCCGGACCGCCACATCGAGCGGGTCACCGAGCTGCAAGAACTCTTCCTCACGCGCGTCGGGCTCGACATCGGCA
AGGTGTGGGTCGCGGACGACGGCGCCGCCGTGGCGGTCTGGACCACGCCGGAGAGCGTCGAAGCGGGGGCGGTGTTCGCCGAGATCGGCCCGCGCATGGCCGA
GTTGAGCGGTTCCCGGCTGGCCGCGCAGCAACAGATGGAAGGCCTCCTGGCGCCGCACCGGCCCAAGGAGCCCGCGTGGTTCCTGGCCACCGTCGGAGTCTCGC
CCGACCACCAGGGCAAGGGTCTGGGCAGCGCCGTCGTGCTCCCCGGAGTGGAGGCGGCCGAGCGCGCCGGGGTGCCCGCCTTCCTGGAGACCTCCGCGCCCCGC
AACCTCCCCTTCTACGAGCGGCTCGGCTTCACCGTCACCGCCGACGTCGAGGTGCCCGAAGGACCGCGCACCTGGTGCATGACCCGCAAGCCCGGTGCCTGAACG
CGTTAAGTCGACAATCAACCTCTGGATTACAAAATTTGTGAAAGATTGACTGGTATTCTTAACTATGTTGCTCCTTTTACGCTATGTGGATACGCTGCTTTAATGCC
TTTGTATCATGCTATTGCTTCCCGTATGGCTTTCATTTTCTCCTCCTTGTATAAATCCTGGTTGCTGTCTCTTTATGAGGAGTTGTGGCCCGTTGTCAGGCAACGTGG
CGTGGTGTGCACTGTGTTTGCTGACGCAACCCCCACTGGTTGGGGCATTGCCACCACCTGTCAGCTCCTTTCCGGGACTTTCGCTTTCCCCCTCCCTATTGCCACGG
CGGAACTCATCGCCGCCTGCCTTGCCCGCTGCTGGACAGGGGCTCGGCTGTTGGGCACTGACAATTCCGTGGTGTTGTCGGGGAAATCATCGTCCTTTCCTTGGCT
GCTCGCCTGTGTTGCCACCTGGATTCTGCGCGGGACGTCCTTCTGCTACGTCCCTTCGGCCCTCAATCCAGCGGACCTTCCTTCCCGCGGCCTGCTGCCGGCTCTGC
GGCCTCTTCCGCGTCTTCGCCTTCGCCCTCAGACGAGTCGGATCTCCCTTTGGGCCGCCTCCCCGCGTCGACTTTAAGACCAATGACTTACAAGGCAGCTGTAGAT
CTTAGCCACTTTTTAAAAGAAAAGGGGGGACTGGAAGGGCTAATTCACTCCCAACGAAGACAAGATCTGCTTTTTGCTTGTACTGGGTCTCTCTGGTTAGACCAG
ATCTGAGCCTGGGAGCTCTCTGGCTAACTAGGGAACCCACTGCTTAAGCCTCAATAAAGCTTGCCTTGAGTGCTTCAAGTAGTGTGTGCCCGTCTGTTGTGTGACT

CTGGTAACTAGAGATCCCTCAGACCCTTTTAGTCAGTGTGGAAAATCTCTAGCAGTACGTATAGTAGTTCATGTCATCTTATTATTCAGTATTTATAACTTGCAAA
GAAATGAATATCAGAGAGTGAGAGGAACTTGTTTATTGCAGCTTATAATGGTTACAAATAAAGCAATAGCATCACAAATTTCACAAATAAAGCATTTTTTTCACT
GCATTCTAGTTGTGGTTTGTCCAAACTCATCAATGTATCTTATCATGTCTGGCTCTAGCTATCCCGCCCCTAACTCCGCCCATCCCGCCCCTAACTCCGCCCAGTTC
CGCCCATTCTCCGCCCCATGGCTGACTAATTTTTTTTATTTATGCAGAGGCCGAGGCCGCCTCGGCCTCTGAGCTATTCCAGAAGTAGTGAGGAGGCTTTTTTGGA
GGCCTAGGGACGTACCCAATTCGCCCTATAGTGAGTCGTATTACGCGCGCTCACTGGCCGTCGTTTTACAACGTCGTGACTGGGAAAACCCTGGCGTTACCCAAC
TTAATCGCCTTGCAGCACATCCCCCTTTCGCCAGCTGGCGTAATAGCGAAGAGGCCCGCACCGATCGCCCTTCCCAACAGTTGCGCAGCCTGAATGGCGAATGGG
ACGCGCCCTGTAGCGGCGCATTAAGCGCGGCGGGTGTGGTGGTTACGCGCAGCGTGACCGCTACACTTGCCAGCGCCCTAGCGCCCGCTCCTTTCGCTTTCTTCCC
TTCCTTTCTCGCCACGTTCGCCGGCTTTCCCCGTCAAGCTCTAAATCGGGGGCTCCCTTTAGGGTTCCGATTTAGTGCTTTACGGCACCTCGACCCCAAAAAACTTG
ATTAGGGTGATGGTTCACGTAGTGGGCCATCGCCCTGATAGACGGTTTTTCGCCCTTTGACGTTGGAGTCCACGTTCTTTAATAGTGGACTCTTGTTCCAAACTGG
AACAACACTCAACCCTATCTCGGTCTATTCTTTTGATTTATAAGGGATTTTGCCGATTTCGGCCTATTGGTTAAAAAATGAGCTGATTTAACAAAAATTTAACGCG
AATTTTAACAAAATATTAACGCTTACAATTTAGGTGGCACTTTTCGGGGAAATGTGCGCGGAACCCCTATTTGTTTATTTTTCTAAATACATTCAAATATGTATCCG
CTCATGAGACAATAACCCTGATAAATGCTTCAATAATATTGAAAAAGGAAGAGTATGAGTATTCAACATTTCCGTGTCGCCCTTATTCCCTTTTTTGCGGCATTTT
GCCTTCCTGTTTTTGCTCACCCAGAAACGCTGGTGAAAGTAAAAGATGCTGAAGATCAGTTGGGTGCACGAGTGGGTTACATCGAACTGGATCTCAACAGCGGTA
AGATCCTTGAGAGTTTTCGCCCCGAAGAACGTTTTCCAATGATGAGCACTTTTAAAGTTCTGCTATGTGGCGCGGTATTATCCCGTATTGACGCCGGGCAAGAGCA
ACTCGGTCGCCGCATACACTATTCTCAGAATGACTTGGTTGAGTACTCACCAGTCACAGAAAAGCATCTTACGGATGGCATGACAGTAAGAGAATTATGCAGTGC
TGCCATAACCATGAGTGATAACACTGCGGCCAACTTACTTCTGACAACGATCGGAGGACCGAAGGAGCTAACCGCTTTTTTGCACAACATGGGGGATCATGTAAC
TCGCCTTGATCGTTGGGAACCGGAGCTGAATGAAGCCATACCAAACGACGAGCGTGACACCACGATGCCTGTAGCAATGGCAACAACGTTGCGCAAACTATTAA
CTGGCGAACTACTTACTCTAGCTTCCCGGCAACAATTAATAGACTGGATGGAGGCGGATAAAGTTGCAGGACCACTTCTGCGCTCGGCCCTTCCGGCTGGCTGGT
TTATTGCTGATAAATCTGGAGCCGGTGAGCGTGGGTCTCGCGGTATCATTGCAGCACTGGGGCCAGATGGTAAGCCCTCCCGTATCGTAGTTATCTACACGACGG
GGAGTCAGGCAACTATGGATGAACGAAATAGACAGATCGCTGAGATAGGTGCCTCACTGATTAAGCATTGGTAACTGTCAGACCAAGTTTACTCATATATACTTT
AGATTGATTTAAAACTTCATTTTTAATTTAAAAGGATCTAGGTGAAGATCCTTTTTGATAATCTCATGACCAAAATCCCTTAACGTGAGTTTTCGTTCCACTGAGCG
TCAGACCCCGTAGAAAAGATCAAAGGATCTTCTTGAGATCCTTTTTTTCTGCGCGTAATCTGCTGCTTGCAAACAAAAAAACCACCGCTACCAGCGGTGGTTTGTT
TGCCGGATCAAGAGCTACCAACTCTTTTTCCGAAGGTAACTGGCTTCAGCAGAGCGCAGATACCAAATACTGTTCTTCTAGTGTAGCCGTAGTTAGGCCACCACTT
CAAGAACTCTGTAGCACCGCCTACATACCTCGCTCTGCTAATCCTGTTACCAGTGGCTGCTGCCAGTGGCGATAAGTCGTGTCTTACCGGGTTGGACTCAAGACGA
TAGTTACCGGATAAGGCGCAGCGGTCGGGCTGAACGGGGGGTTCGTGCACACAGCCCAGCTTGGAGCGAACGACCTACACCGAACTGAGATACCTACAGCGTGA
GCTATGAGAAAGCGCCACGCTTCCCGAAGGGAGAAAGGCGGACAGGTATCCGGTAAGCGGCAGGGTCGGAACAGGAGAGCGCACGAGGGAGCTTCCAGGGGG
AAACGCCTGGTATCTTTATAGTCCTGTCGGGTTTCGCCACCTCTGACTTGAGCGTCGATTTTTGTGATGCTCGTCAGGGGGGCGGAGCCTATGGAAAAACGCCAGC
AACGCGGCCTTTTTACGGTTCCTGGCCTTTTGCTGGCCTTTTGCTCACATGTTCTTTCCTGCGTTATCCCCTGATTCTGTGGATAACCGTATTACCGCCTTTGAGTGA
GCTGATACCGCTCGCCGCAGCCGAACGACCGAGCGCAGCGAGTCAGTGAGCGAGGAAGCGGAAGAGCGCCCAATACGCAAACCGCCTCTCCCCGCGCGTTGGCC
GATTCATTAATGCAGCTGGCACGACAGGTTTCCCGACTGGAAAGCGGGCAGTGAGCGCAACGCAATTAATGTGAGTTAGCTCACTCATTAGGCACCCCAGGCTTT
ACACTTTATGCTTCCGGCTCGTATGTTGTGTGGAATTGTGAGCGGATAACAATTTCACACAGGAAACAGCTATGACCATGATTACGCCAAGCGCGCAATTAACCC
TCACTAAAGGGAACAAAAGCTGGAGCTGCAAGCTTAATGTAGTCTTATGCAATACTCTTGTAGTCTTGCAACATGGTAACGATGAGTTAGCAACATGCCTTACAA
GGAGAGAAAAAGCACCGTGCATGCCGATTGGTGGAAGTAAGGTGGTACGATCGTGCCTTATTAGGAAGGCAACAGACGGGTCTGACATGGATTGGACGAACCAC
TGAATTGCCGCATTGCAGAGATATTGTATTTAAGTGCCTAGCTCGATACATAAACGGGTCTCTCTGGTTAGACCAGATCTGAGCCTGGGAGCTCTCTGGCTAACTA
GGGAACCCACTGCTTAAGCCTCAATAAAGCTTGCCTTGAGTGCTTCAAGTAGTGTGTGCCCGTCTGTTGTGTGACTCTGGTAACTAGAGATCCCTCAGACCCTTTT
AGTCAGTGTGGAAAATCTCTAGCAGTGGCGCCCGAACAGGGACTTGAAAGCGAAAGGGAAACCAGAGGAGCTCTCTCGACGCAGGACTCGGCTTGCTGAAGCGC
GCACGGCAAGAGGCGAGGGGCGGCGACTGGTGAGTACGCCAAAAATTTTGACTAGCGGAGGCTAGAAGGAGAGAGATGGGTGCGAGAGCGTCAGTATTAAGCG
GGGGAGAATTAGATCGCGATGGGAAAAAATTCGGTTAAGGCCAGGGGGAAAGAAAAAATATAAATTAAAACATATAGTATGGGCAAGCAGGGAGCTAGAACGA
TTCGCAGTTAATCCTGGCCTGTTAGAAACATCAGAAGGCTGTAGACAAATACTGGGACAGCTACAACCATCCCTTCAGACAGGATCAGAAGAACTTAGATCATTA
TATAATACAGTAGCAACCCTCTATTGTGTGCATCAAAGGATAGAGATAAAAGACACCAAGGAAGCTTTAGACAAGATAGAGGAAGAGCAAAACAAAAGTAAGA
CCACCGCACAGCAAGCGGCCGCTGATCTTCAGACCTGGAGGAGGAGATATGAGGGACAATTGGAGAAGTGAATTATATAAATATAAAGTAGTAAAAATTGAACC
ATTAGGAGTAGCACCCACCAAGGCAAAGAGAAGAGTGGTGCAGAGAGAAAAAAGAGCAGTGGGAATAGGAGCTTTGTTCCTTGGGTTCTTGGGAGCAGCAGGA
AGCACTATGGGCGCAGCGTCAATGACGCTGACGGTACAGGCCAGACAATTATTGTCTGGTATAGTGCAGCAGCAGAACAATTTGCTGAGGGCTATTGAGGCGCA
ACAGCATCTGTTGCAACTCACAGTCTGGGGCATCAAGCAGCTCCAGGCAAGAATCCTGGCTGTGGAAAGATACCTAAAGGATCAACAGCTCCTGGGGATTTGGG
GTTGCTCTGGAAAACTCATTTGCACCACTGCTGTGCCTTGGAATGCTAGTTGGAGTAATAAATCTCTGGAACAGATTTGGAATCACACGACCTGGATGGAGTGGG
ACAGAGAAATTAACAATTACACAAGCTTAATACACTCCTTAATTGAAGAATCGCAAAACCAGCAAGA

10000BP BOT:
TCTTGCTGGTTTTGCGATTCTTCAATTAAGGAGTGTATTAAGCTTGTGTAATTGTTAATTTCTCTGTCCCACTCCATCCAGGTCGTGTGATTCCAAATCTGTTCCAGA
GATTTATTACTCCAACTAGCATTCCAAGGCACAGCAGTGGTGCAAATGAGTTTTCCAGAGCAACCCCAAATCCCCAGGAGCTGTTGATCCTTTAGGTATCTTTCCA
CAGCCAGGATTCTTGCCTGGAGCTGCTTGATGCCCCAGACTGTGAGTTGCAACAGATGCTGTTGCGCCTCAATAGCCCTCAGCAAATTGTTCTGCTGCTGCACTAT
ACCAGACAATAATTGTCTGGCCTGTACCGTCAGCGTCATTGACGCTGCGCCCATAGTGCTTCCTGCTGCTCCCAAGAACCCAAGGAACAAAGCTCCTATTCCCACT
GCTCTTTTTTCTCTCTGCACCACTCTTCTCTTTGCCTTGGTGGGTGCTACTCCTAATGGTTCAATTTTTACTACTTTATATTTATATAATTCACTTCTCCAATTGTCCC
TCATATCTCCTCCTCCAGGTCTGAAGATCAGCGGCCGCTTGCTGTGCGGTGGTCTTACTTTTGTTTTGCTCTTCCTCTATCTTGTCTAAAGCTTCCTTGGTGTCTTTT
ATCTCTATCCTTTGATGCACACAATAGAGGGTTGCTACTGTATTATATAATGATCTAAGTTCTTCTGATCCTGTCTGAAGGGATGGTTGTAGCTGTCCCAGTATTTG
TCTACAGCCTTCTGATGTTTCTAACAGGCCAGGATTAACTGCGAATCGTTCTAGCTCCCTGCTTGCCCATACTATATGTTTTAATTTATATTTTTTCTTTCCCCCTGG
CCTTAACCGAATTTTTTCCCATCGCGATCTAATTCTCCCCCGCTTAATACTGACGCTCTCGCACCCATCTCTCTCCTTCTAGCCTCCGCTAGTCAAAATTTTTGGCGT
ACTCACCAGTCGCCGCCCCTCGCCTCTTGCCGTGCGCGCTTCAGCAAGCCGAGTCCTGCGTCGAGAGAGCTCCTCTGGTTTCCCTTTCGCTTTCAAGTCCCTGTTCG
GGCGCCACTGCTAGAGATTTTCCACACTGACTAAAAGGGTCTGAGGGATCTCTAGTTACCAGAGTCACACAACAGACGGGCACACACTACTTGAAGCACTCAAG
GCAAGCTTTATTGAGGCTTAAGCAGTGGGTTCCCTAGTTAGCCAGAGAGCTCCCAGGCTCAGATCTGGTCTAACCAGAGAGACCCGTTTATGTATCGAGCTAGGC
ACTTAAATACAATATCTCTGCAATGCGGCAATTCAGTGGTTCGTCCAATCCATGTCAGACCCGTCTGTTGCCTTCCTAATAAGGCACGATCGTACCACCTTACTTC
CACCAATCGGCATGCACGGTGCTTTTTCTCTCCTTGTAAGGCATGTTGCTAACTCATCGTTACCATGTTGCAAGACTACAAGAGTATTGCATAAGACTACATTAAG
CTTGCAGCTCCAGCTTTTGTTCCCTTTAGTGAGGGTTAATTGCGCGCTTGGCGTAATCATGGTCATAGCTGTTTCCTGTGTGAAATTGTTATCCGCTCACAATTCCA
CACAACATACGAGCCGGAAGCATAAAGTGTAAAGCCTGGGGTGCCTAATGAGTGAGCTAACTCACATTAATTGCGTTGCGCTCACTGCCCGCTTTCCAGTCGGGA
AACCTGTCGTGCCAGCTGCATTAATGAATCGGCCAACGCGCGGGGAGAGGCGGTTTGCGTATTGGGCGCTCTTCCGCTTCCTCGCTCACTGACTCGCTGCGCTCGG
TCGTTCGGCTGCGGCGAGCGGTATCAGCTCACTCAAAGGCGGTAATACGGTTATCCACAGAATCAGGGGATAACGCAGGAAAGAACATGTGAGCAAAAGGCCAG
CAAAAGGCCAGGAACCGTAAAAAGGCCGCGTTGCTGGCGTTTTTCCATAGGCTCCGCCCCCCTGACGAGCATCACAAAAATCGACGCTCAAGTCAGAGGTGGCG
AAACCCGACAGGACTATAAAGATACCAGGCGTTTCCCCCTGGAAGCTCCCTCGTGCGCTCTCCTGTTCCGACCCTGCCGCTTACCGGATACCTGTCCGCCTTTCTC
CCTTCGGGAAGCGTGGCGCTTTCTCATAGCTCACGCTGTAGGTATCTCAGTTCGGTGTAGGTCGTTCGCTCCAAGCTGGGCTGTGTGCACGAACCCCCCGTTCAGC
CCGACCGCTGCGCCTTATCCGGTAACTATCGTCTTGAGTCCAACCCGGTAAGACACGACTTATCGCCACTGGCAGCAGCCACTGGTAACAGGATTAGCAGAGCGA
GGTATGTAGGCGGTGCTACAGAGTTCTTGAAGTGGTGGCCTAACTACGGCTACACTAGAAGAACAGTATTTGGTATCTGCGCTCTGCTGAAGCCAGTTACCTTCG
GAAAAAGAGTTGGTAGCTCTTGATCCGGCAAACAAACCACCGCTGGTAGCGGTGGTTTTTTTGTTTGCAAGCAGCAGATTACGCGCAGAAAAAAAGGATCTCAA
GAAGATCCTTTGATCTTTTCTACGGGGTCTGACGCTCAGTGGAACGAAAACTCACGTTAAGGGATTTTGGTCATGAGATTATCAAAAAGGATCTTCACCTAGATCC
TTTTAAATTAAAAATGAAGTTTTAAATCAATCTAAAGTATATATGAGTAAACTTGGTCTGACAGTTACCAATGCTTAATCAGTGAGGCACCTATCTCAGCGATCTG
TCTATTTCGTTCATCCATAGTTGCCTGACTCCCCGTCGTGTAGATAACTACGATACGGGAGGGCTTACCATCTGGCCCCAGTGCTGCAATGATACCGCGAGACCCA
CGCTCACCGGCTCCAGATTTATCAGCAATAAACCAGCCAGCCGGAAGGGCCGAGCGCAGAAGTGGTCCTGCAACTTTATCCGCCTCCATCCAGTCTATTAATTGT
TGCCGGGAAGCTAGAGTAAGTAGTTCGCCAGTTAATAGTTTGCGCAACGTTGTTGCCATTGCTACAGGCATCGTGGTGTCACGCTCGTCGTTTGGTATGGCTTCAT
TCAGCTCCGGTTCCCAACGATCAAGGCGAGTTACATGATCCCCCATGTTGTGCAAAAAAGCGGTTAGCTCCTTCGGTCCTCCGATCGTTGTCAGAAGTAAGTTGGC

CGCAGTGTTATCACTCATGGTTATGGCAGCACTGCATAATTCTCTTACTGTCATGCCATCCGTAAGATGCTTTTCTGTGACTGGTGAGTACTCAACCAAGTCATTCT
GAGAATAGTGTATGCGGCGACCGAGTTGCTCTTGCCCGGCGTCAATACGGGATAATACCGCGCCACATAGCAGAACTTTAAAAGTGCTCATCATTGGAAAACGTT
CTTCGGGGCGAAAACTCTCAAGGATCTTACCGCTGTTGAGATCCAGTTCGATGTAACCCACTCGTGCACCCAACTGATCTTCAGCATCTTTTACTTTCACCAGCGT
TTCTGGGTGAGCAAAAACAGGAAGGCAAAATGCCGCAAAAAAGGGAATAAGGGCGACACGGAAATGTTGAATACTCATACTCTTCCTTTTTCAATATTATTGAA
GCATTTATCAGGGTTATTGTCTCATGAGCGGATACATATTTGAATGTATTTAGAAAAATAAACAAATAGGGGTTCCGCGCACATTTCCCCGAAAAGTGCCACCTA
AATTGTAAGCGTTAATATTTTGTTAAAATTCGCGTTAAATTTTTGTTAAATCAGCTCATTTTTTAACCAATAGGCCGAAATCGGCAAAATCCCTTATAAATCAAAA
GAATAGACCGAGATAGGGTTGAGTGTTGTTCCAGTTTGGAACAAGAGTCCACTATTAAAGAACGTGGACTCCAACGTCAAAGGGCGAAAAACCGTCTATCAGGG
CGATGGCCCACTACGTGAACCATCACCCTAATCAAGTTTTTTGGGGTCGAGGTGCCGTAAAGCACTAAATCGGAACCCTAAAGGGAGCCCCCGATTTAGAGCTTG
ACGGGGAAAGCCGGCGAACGTGGCGAGAAAGGAAGGGAAGAAAGCGAAAGGAGCGGGCGCTAGGGCGCTGGCAAGTGTAGCGGTCACGCTGCGCGTAACCAC
CACACCCGCCGCGCTTAATGCGCCGCTACAGGGCGCGTCCCATTCGCCATTCAGGCTGCGCAACTGTTGGGAAGGGCGATCGGTGCGGGCCTCTTCGCTATTACG
CCAGCTGGCGAAAGGGGGATGTGCTGCAAGGCGATTAAGTTGGGTAACGCCAGGGTTTTCCCAGTCACGACGTTGTAAAACGACGGCCAGTGAGCGCGCGTAAT
ACGACTCACTATAGGGCGAATTGGGTACGTCCCTAGGCCTCCAAAAAAGCCTCCTCACTACTTCTGGAATAGCTCAGAGGCCGAGGCGGCCTCGGCCTCTGCATA
AATAAAAAAAATTAGTCAGCCATGGGGCGGAGAATGGGCGGAACTGGGCGGAGTTAGGGGCGGGATGGGCGGAGTTAGGGGCGGGATAGCTAGAGCCAGACAT
GATAAGATACATTGATGAGTTTGGACAAACCACAACTAGAATGCAGTGAAAAAAATGCTTTATTTGTGAAATTTGTGATGCTATTGCTTTATTTGTAACCATTATA
AGCTGCAATAAACAAGTTCCTCTCACTCTCTGATATTCATTTCTTTGCAAGTTATAAATACTGAATAATAAGATGACATGAACTACTATACGTACTGCTAGAGATT
TTCCACACTGACTAAAAGGGTCTGAGGGATCTCTAGTTACCAGAGTCACACAACAGACGGGCACACACTACTTGAAGCACTCAAGGCAAGCTTTATTGAGGCTTA
AGCAGTGGGTTCCCTAGTTAGCCAGAGAGCTCCCAGGCTCAGATCTGGTCTAACCAGAGAGACCCAGTACAAGCAAAAAGCAGATCTTGTCTTCGTTGGGAGTGA
ATTAGCCCTTCCAGTCCCCCCTTTTCTTTTAAAAAGTGGCTAAGATCTACAGCTGCCTTGTAAGTCATTGGTCTTAAAGTCGACGCGGGGAGGCGGCCCAAAGGGA
GATCCGACTCGTCTGAGGGCGAAGGCGAAGACGCGGAAGAGGCCGCAGAGCCGGCAGCAGGCCGCGGGAAGGAAGGTCCGCTGGATTGAGGGCCGAAGGGAC
GTAGCAGAAGGACGTCCCGCGCAGAATCCAGGTGGCAACACAGGCGAGCAGCCAAGGAAAGGACGATGATTTCCCCGACAACACCACGGAATTGTCAGTGCCC
AACAGCCGAGCCCCTGTCCAGCAGCGGGCAAGGCAGGCGGCGATGAGTTCCGCCGTGGCAATAGGGAGGGGGAAAGCGAAAGTCCCGGAAAGGAGCTGACAGG
TGGTGGCAATGCCCCAACCAGTGGGGGTTGCGTCAGCAAACACAGTGCACACCACGCCACGTTGCCTGACAACGGGCCACAACTCCTCATAAAGAGACAGCAAC
CAGGATTTATACAAGGAGGAGAAAATGAAAGCCATACGGGAAGCAATAGCATGATACAAAGGCATTAAAGCAGCGTATCCACATAGCGTAAAAGGAGCAACAT
AGTTAAGAATACCAGTCAATCTTTCACAAATTTTGTAATCCAGAGGTTGATTGTCGACTTAACGCGTTCAGGCACCGGGCTTGCGGGTCATGCACCAGGTGCGCG
GTCCTTCGGGCACCTCGACGTCGGCGGTGACGGTGAAGCCGAGCCGCTCGTAGAAGGGGAGGTTGCGGGGCGCGGAGGTCTCCAGGAAGGCGGGCACCCCGGC
GCGCTCGGCCGCCTCCACTCCGGGGAGCACGACGGCGCTGCCCAGACCCTTGCCCTGGTGGTCGGGCGAGACTCCGACGGTGGCCAGGAACCACGCGGGCTCCT
TGGGCCGGTGCGGCGCCAGGAGGCCTTCCATCTGTTGCTGCGCGGCCAGCCGGGAACCGCTCAACTCGGCCATGCGCGGGCCGATCTCGGCGAACACCGCCCCC
GCTTCGACGCTCTCCGGCGTGGTCCAGACCGCCACGGCGGCGCCGTCGTCCGCGACCCACACCTTGCCGATGTCGAGCCCGACGCGCGTGAGGAAGAGTTCTTGC
AGCTCGGTGACCCGCTCGATGTGGCGGTCCGGATCGACGGTGTGGCGCGTGGCGGGGTAGTCGGCGAACGCGGCGGCGAGGGTGCGTACGGCCCTGGGGACGTC
GTCGCGGGTGGCGAGGCGCACCGTGGGCTTGTACTCGGTCATGGTGGCCGTACGTCACGACACCTGAAATGGAAGAAAAAAACTTTGAACCACTGTCTGAGGCTT
GAGAATGAACCAAGATCCAAACTCAAAAAGGGCAAATTCCAAGGAGAATTACATCAAGTGCCAAGCTGGCCTAACTTCAGTCTCCACCCACTCAGTGTGGGGAA
ACTCCATCGCATAAAACCCCTCCCCCCAACCTAAAGACGACGTACTCCAAAAGCTCGAGAACTAATCGAGGTGCCTGGACGGCGCCCGGTACTCCGTGGAGTCAC
ATGAAGCGACGGCTGAGGACGGAAAGGCCCTTTTCCTTTGTGTGGGTGACTCACCCGCCCGCTCTCCCGAGCGCCGCGTCCTCCATTTTGAGCTCCCTGCAGCAG
GGCCGGGAAGCGGCCATCTTTCCGCTCACGCAACTGGTGCCGACCGGGCCAGCCTTGCCGCCCAGGGCGGGGCGATACACGGCGGCGCGAGGCCAGGCACCAGA
GCAGGCCGGCCAGCTTGAGACTACCCCCGTCCGATTCTCGGTGGCCGCGCTCGCAGGCCCCGCCTCGCCGAACATGTGCGCTGGGACGCACGGGCCCCGTCGCCG
CCCGCGGCCCCAAAAACCGAAATACCAGTGTGCAGATCTTGGCCCGCATTTACAAGACTATCTTGCCAGAAAAAAAGCGTCGCAGCAGGTCATCAAAAATTTTA
AATGGCTAGAGACTTATCGAAAGCAGCGAGACAGGCGCGAAGGTGCCACCAGATTCGCACGCGGCGGCCCCAGCGCCCAGGCCAGGCCTCAACTCAAGCACGA
GGCGAAGGGGCTCCTTAAGCGCAAGGCCTCGAACTCTCCCACCCACTTCCAACCCGAAGCTCGGGATCAAGAATCACGTACTGCAGCCAGGTGGAAGTAATTCA
AGGCACGCAAGGGCCATAACCCGTAAAGAGGCCAGGCCCGCGGGAACCACACACGGCACTTACCTGTGTTCTGGCGGCAAACCCGTTGCGAAAAAGAACGTTCA
CGGCGACTACTGCACTTATATACGGTTCTCCCCCACCCTCGGGAAAAAGGCGGAGCCAGTACACGACATCACTTTCCCAGTTTACCCCGCGCCACCTTCTCTAGGC
ACCGGATCAATTGCCGACCCCTCCCCCCAACTTCTCGGGGACTGTGGGCGATGTGCGCTCTGCCCACTGACGGGCACCGGAGCCAATTCCCACTCCTTTCAAGAC
CTAGAAGGTCCATTAGCTGCAAAGATTCCTCTCTGTTTAAAACTTTATCCATCTTTGCACCCGGGCCCCCTCGAGCCGGCGCCAAAGTGGATCTCTGCTGTCCCTG
TAATAAACCCGAAAATTTTGAATTTTTGTAATTTGTTTTTGTAATTCTTTAGTTTGTATGTCTGTTGCTATTATGTCTACTATTCTTTCCCCTGCACTGTACCCCCCAA
TCCCCCCTTTTCTTTTAAAATTGTGGATGAATACTGCCATTTGTCTCAAGATCTAGTTACGCCAAGCTTAAAAAAGCACCGACTCGGTGCCACTTTTTCAAGTTGAT
AACGGACTAGCCTTATTTTAACTTGCTATTTCTAGCTCTAAAACAGAGACGTACAAAAAAGAGCAAGAAGCTAAAAAAGATTTAAAAATTATTTTTAGCGCAGTT
AATGGAACAGGAACTAAATTTACCCCAAAAATATTACGTGAATCAGGATATAACGTTATTGAGGTTGAAGAGCATGCATTTGAAGATGAAACATTTAAAAATGTT
GTAAATCCAAATCCAGAATTTGATCCTGCATGAAAAATACCGCTTGAATATGGTATTAAACATGATGCAGATATTATTATTATGAATGACCCAGATGCTGACAGA
TTTGGAATGGCAATAAAACATGATGGTCATTTTGTAAGATTAGATGGAAATCAAACAGGACCAATTTTAATTGATTGAAAATTATCAAATCTAAAACGCTTAAAT
AGCATTCCAAAAAATCCGGCTCTATATTCAAGTTTTGTAACAAGTGATTTGGGTGATAGAATCGCTCATGAAAAATATGGAGTTAATATTGTAAAAACTTTAACTG
GATTTAAATGAATGGGTAGAGAAATTGCTAAAGAAGAAGATAACGGATTAAATTTTGTTTTTGCTTATGAAGAAAGTTATGGATATGTAATTGATGACTCAGCTA
GAGATAAAGATGGAATACAAGCTTCTATATTAATAGCAGAGGCTGCTTGATTTTATAAAAAACAAAATAAAACATTAGTAGACTATTTAGAAGATTTATTTAAAG
AAATGGGTGCATATTACACTTTCACTTTAAACTTGAATTTTAAACCAGAAGAAAAGAAATTAAAAATTGAACCATTAATGAAATCATTGAGAGCAACACCCTTAA
CTCAAATTGCTGGACTTAAAGTTGTTAATGTTGAAGACTACATCGATGGAATGTATAATATGCCAGGACAAGACTTACTAAAATTTTATTTAGAAGATAAGTCAT
GATTTGCTGTTCGCCCAAGTGGAACTGAACCTAAACTAAAAATTTATTTTATAGGTGTTGGTGAATCTGTTCAAAACGCTAAAGTTAAAGTAGACGAAATTATTAA
AGAATTAAAATTAAAAATGAATATATAGGAGAAAAAATGAAACTAAACAAATATATAGATCACACATTATTAAAACAAGATGCTACGAAAGCTGAAATTAAACA
ATTATGTGATGAAGCAATTGAATTTGATTTTGCAACAGTTTGTGTTAATTCATATTGAACAAGCTATTGTAAAGAATTATTAAAAGGCACAAATGTAGGAATAAC
AAATGTTGTAGGTTTTCCTCTAGGTGCATGCACAACAGCTACAAAAGCATTCGAAGTTTCTGAAGCAATTAAAGATGGTGCAACAGAAATTGATATGGTATTAAA
TATTGGTGCATTAAAAGACAAAAATTATGAATTAGTTTTAGAAGACATGAAAGCTGTAAAAAAAGCAGCTGGATCACATGTTGTTAAATGTATTATGGAAAATTG
TTTATTAACAAAAGAAGAAATCATGAAAGCTTGTGAAATAGCTGTTGAAGCTGGATTAGAATTTGTTAAAACATCAACAGGATTTTCAAAATCAGGTGCAACATT
TGAAGATGTTAAACTAATGAAGTCAGTTGTTAAAGACAATGCTTTAGTTAAAGCAGCTGGTGGAGTTAGAACATTTGAAGATGCTCAAAAAATGATTGAAGCAG
GAGCTGACCGCTTAGGAACAAGTGGTGGAGTAGCTATTATTAAAGGTGAAGAAAACAACGCGAGTTACTAAAACTAGCGTTTTTTTATTTTGCTCATTTTTATTAA
AAGTTTGCAAAAAGGAACATAAAAATTCTAATTATTGATACTAAAGTTATTAAAAAGAAGATTTTGGTTGATTTTATAAAGGTCATAGAATATAATATTTTAGCAT
GTGTATTTTGTGTGCTCATTTACAACCGTCTCCGGTGTTTCGTCCTTTCCACAAGATATATAAAGCCAAGAAATCGAAATACTTTCAAGTTACGGTAAGCATATGA
TAGTCCATTTTAAAACATAATTTTAAAACTGCAAACTACCCAAGAAATTATTACTTTCTACGTCACGTATTTTGTACTAATATCTTTGTGTTTACAGTCAAATTAAT
TCTAATTATCTCTCTAACAGCCTTGTATCGTATATGCAAATATGAAGGAATCATGGGAAATAGGCCCTCTCTGGGTCCCCTCGGGGTTGGGAGGTGGGTCTGAAAC
GAT

Supplementary Figure 2: The second ISD library of larger PCR-amplified dsDNA.
(A) Gel Electrophoresis of PCR-amplified dsDNA. The NEB 1 kb DNA Ladder was used for reference. **(B)** Sequences for each PCR-amplified dsDNA molecules. Primers used for each PCR-amplification were determined using the NCBI Primer Blast tool website, ncbi.nlm.nih.gov/tools/primer-blast/ and are highlighted in yellow for the forward primers and in red for the reverse primers.

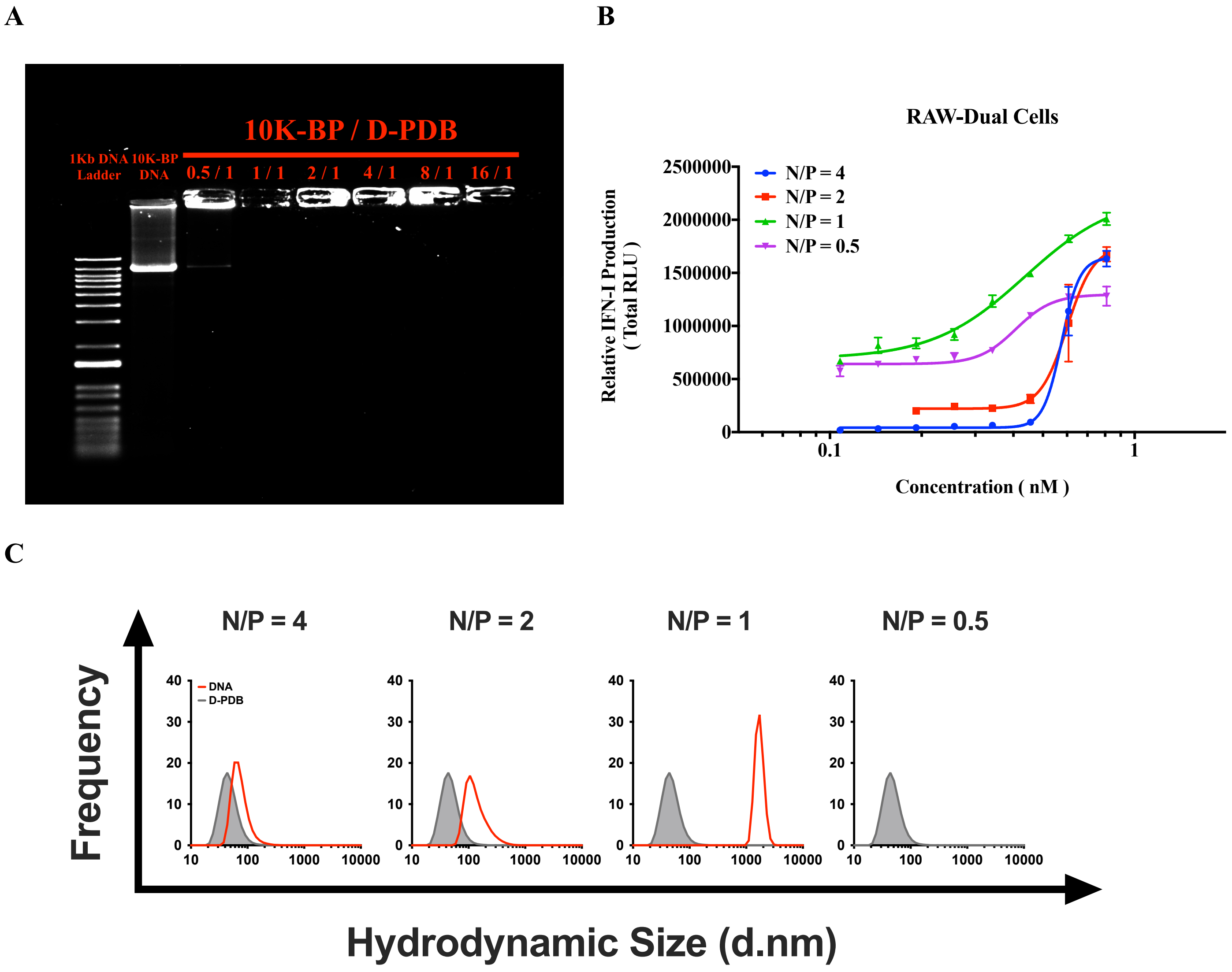

Supplementary Figure 3: Effects of N/P Charge Ratio.
(A) Agarose gel image. Lanes comprise 1 µg DNA mixed with the indicated amount of D-PDB. The TrackIt^{TM} 1 Kb Plus DNA Ladder was used for reference. PCR-amplified 10,000-BP dsDNA / D-PDB at varying N/P charge ratios was tested for DNA loading. **(B)** RAW-Dual reporter cell assay of PCR-amplified 5000-BP dsDNA / D-PDB at varying N/P charge ratios. **(C)** DLS analysis of PCR-amplified 5000-BP dsDNA / D-PDB at varying N/P charge ratios. Frequency indicates the number-based particle size distribution. Hydrodynamic size indicates the particle diameter in nm. N/P = 0.5 was not colloidally stable to collect an accurate size measurement.

A

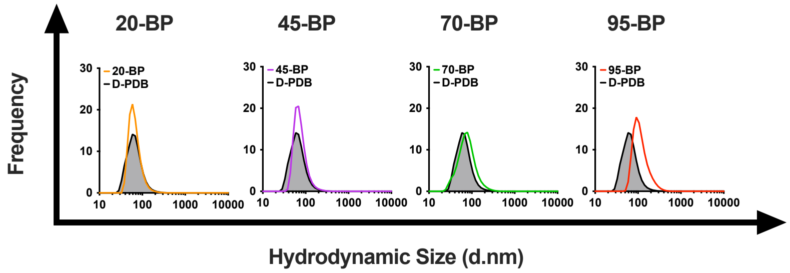

Malvern Zetasizer	Number PSD Peak (Mode)
D-PDB	59 nm
20-BP / D-PDB	59 nm
45-BP / D-PDB	68 nm
70-BP / D-PDB	79 nm
95-BP / D-PDB (NanoISD)	91 nm

B

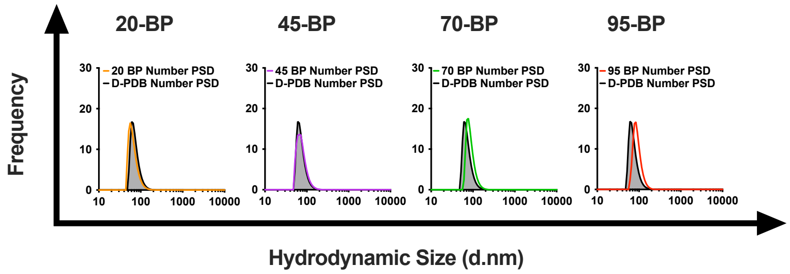

Anton Paar Litesizer	Number PSD Peak (Mode)
D-PDB	61 nm
20-BP / D-PDB	56 nm
45-BP / D-PDB	66 nm
70-BP / D-PDB	77 nm
95-BP / D-PDB (NanoISD)	84 nm

Supplementary Figure 4: DLS analysis of the synthetic, phosphorothioate-capped dsDNA library.
(A) Synthetic, phosphorothioate-capped dsDNA library complexed to D-PDB at an N/P charge ratio of 4. Data presented as number-based particle size distribution. Malvern Zetasizer used for quantification. **(B)** Synthetic, phosphorothioate-capped ISD library complexed to D-PDB at an N/P charge ratio of 4. Data presented as number-based particle size distribution. DLS using an Anton Paar Litesizer was used as a second form of particle size analysis.

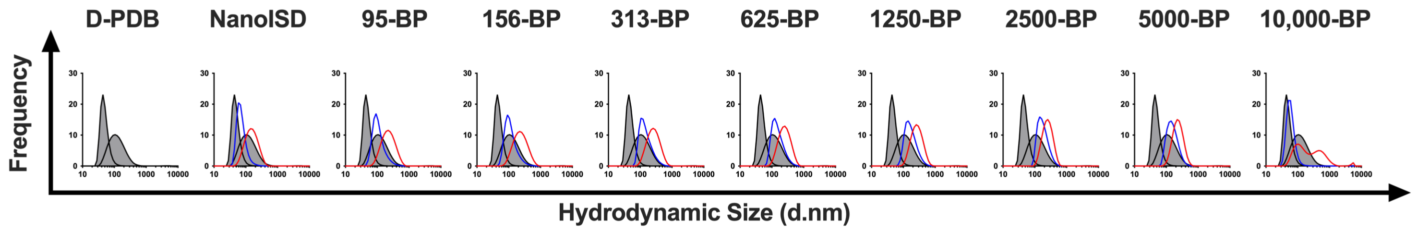

Malvern Zetasizer	Number PSD Peak (Mode)
D-PDB	44 nm
NanoISD	59 nm
95-BP / D-PDB	91 nm
156-BP / D-PDB	91 nm
313-BP / D-PDB	106 nm
625-BP / D-PDB	122 nm
1250-BP / D-PDB	142 nm
2500-BP / D-PDB	142 nm
5000-BP / D-PDB	142 nm
10,000-BP / D-PDB	N/A

Supplementary Figure 5: DLS analysis of the PCR-amplified dsDNA library.
PCR-amplified dsDNA library complexed to D-PDB at an N/P charge ratio of 4. Data presented as both number-based particle size distribution (blue) and intensity-based particle size distribution (red).

A

RAW-Dual Dose Response:

Treatment	Maximum Efficacy (RLU)	EC_{50} Values (nM)
95-BP / D-PDB	396,233	22.4
70-BP / D-PDB	385,838	31.3
45-BP / D-PDB	240,839	43.5
20-BP / D-PDB	84,923	112.9
95-BP	449	N/A
D-PDB	20,651	442.3
cGAMP	522,049	31,579.1

B

THP1-Dual Dose Response:

Treatment	Maximum Efficacy (RLU)	EC_{50} Values (nM)
95-BP / D-PDB	1,648,495	14.1
70-BP / D-PDB	1,089,124	21.8
45-BP / D-PDB	684,302	37.2
20-BP / D-PDB	493,610	115.1
95-BP	1,197	N/A
D-PDB	208,907	537.8
cGAMP	2,514,642	27,759.1

C

A549-Dual Dose Response:

Treatment	Maximum Efficacy (RLU)	EC_{50} Values (nM)
95-BP / D-PDB	1,983,308	19.1
70-BP / D-PDB	1,424,241	24.7
45-BP / D-PDB	749,267	45.0
20-BP / D-PDB	698,816	113.4
95-BP	0	N/A
D-PDB	652,675	535.6
cGAMP	1,695,993	38,503.3

Supplementary Figure 6: Maximum efficacy and EC_{50} values for data presented in *Figure 2*.

A

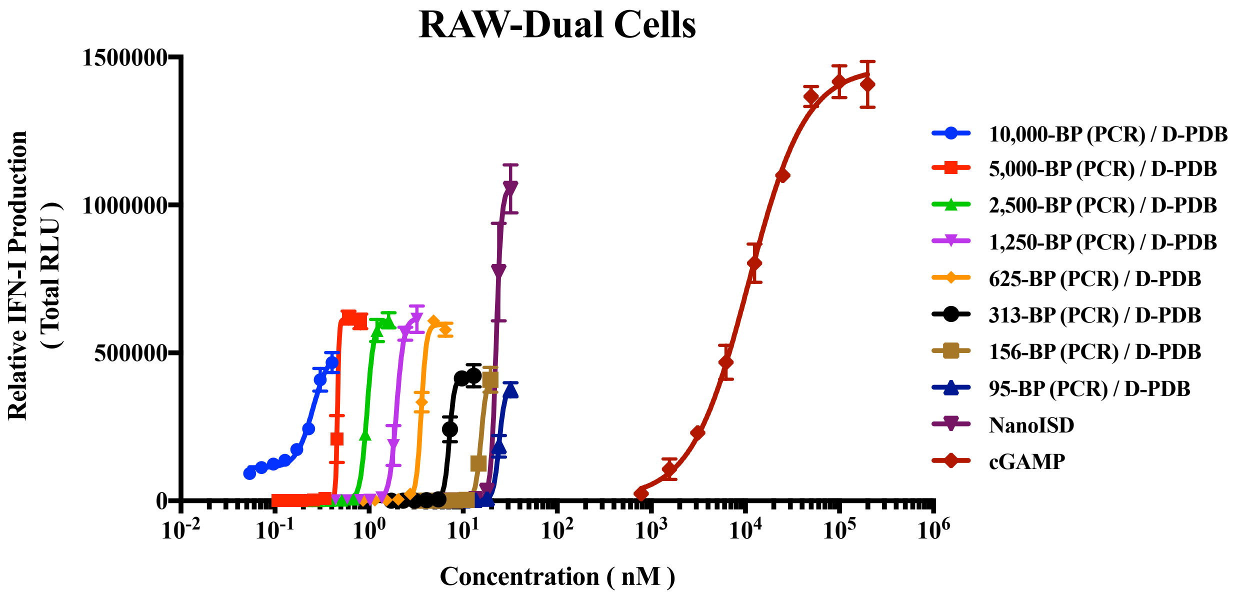

B

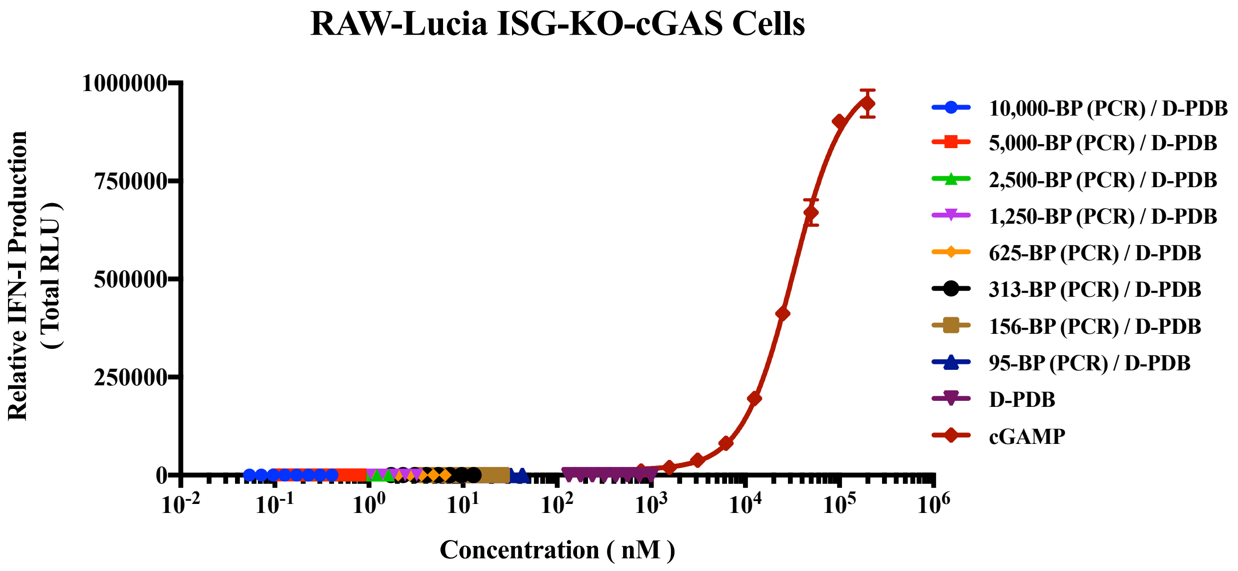

Supplementary Figure 7: Activity of the PCR-amplified dsDNA library with D-PDB.
(A) RAW-Dual IFN-I reporter cell assay of PCR-amplified dsDNA library complexed to D-PDB at an N/P charge ratio of 4. **(B)** RAW-Lucia ISG-KO-cGAS IFN-I reporter cell assay of PCR-amplified dsDNA library complexed to D-PDB at an N/P charge ratio of 4. The dose response curve for free D-PDB is positioned along the x-axis in terms of the molar amount of polymer chains rather than molar amount of loaded dsDNA, and each dose response that utilized the polymer was administered using equivalent D-PDB concentrations.

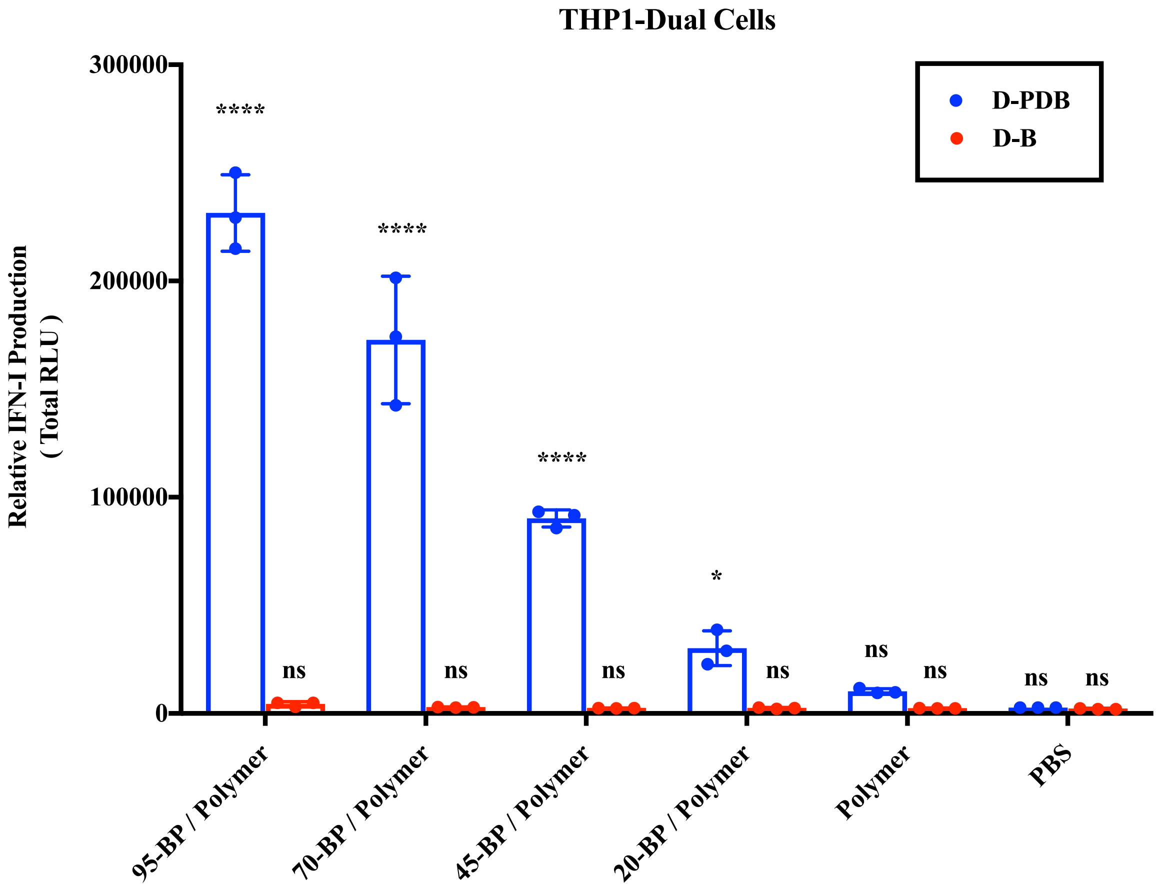

Supplementary Figure 8: Activity of the synthetic ISD library with D-PDB or D-B as carriers.
THP1-Dual IFN-I reporter cells were treated with 1.5 μg/mL DNA using an N/P charge ratio of 5. A two-way ANOVA with Sidak test was used for statistical analysis.

Nanoparticle	Zeta Potential (mV)
D-PDB	+ 16.27
20-BP / D-PDB	+ 9.49
45-BP / D-PDB	+ 15.50
70-BP / D-PDB	+ 13.10
95-BP / D-PDB	+ 14.87

Supplementary Figure 9: Zeta potential of D-PDB and the synthetic ISD library with D-PDB.

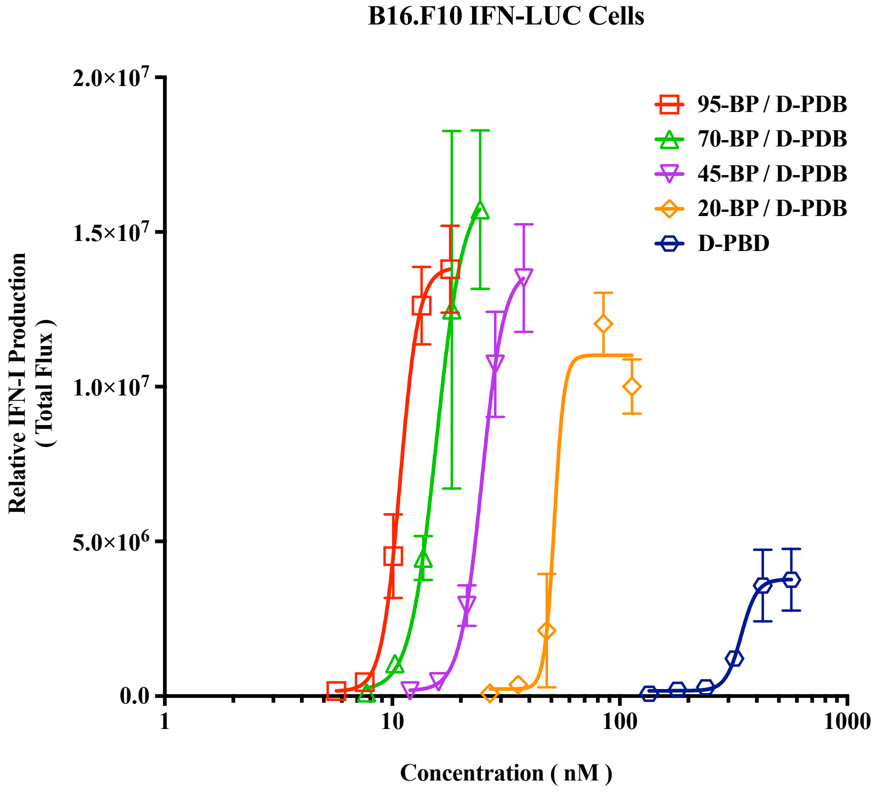

Supplementary Figure 10: Activity of the synthetic ISD library with D-PDB in B16.F10 IFN-LUC cells. B16.F10 IFN-LUC reporter cell assay of synthetic, variable-length ISD library complexed to D-PDB at an N/P charge ratio of 4. EC_{50} values for 95-BP / D-PDB, 70-BP / D-PDB, 45-BP / D-PDB, 20-BP / D-PDB, and D-PDB are 11 nM, 15 nM, 25 nM, 52 nM, and 339 nM, respectively. The dose response curve for free D-PDB is positioned along the x-axis in terms of the molar amount of polymer chains rather than molar amount of loaded dsDNA, and each dose response that utilized the polymer was administered using equivalent D-PDB concentrations.

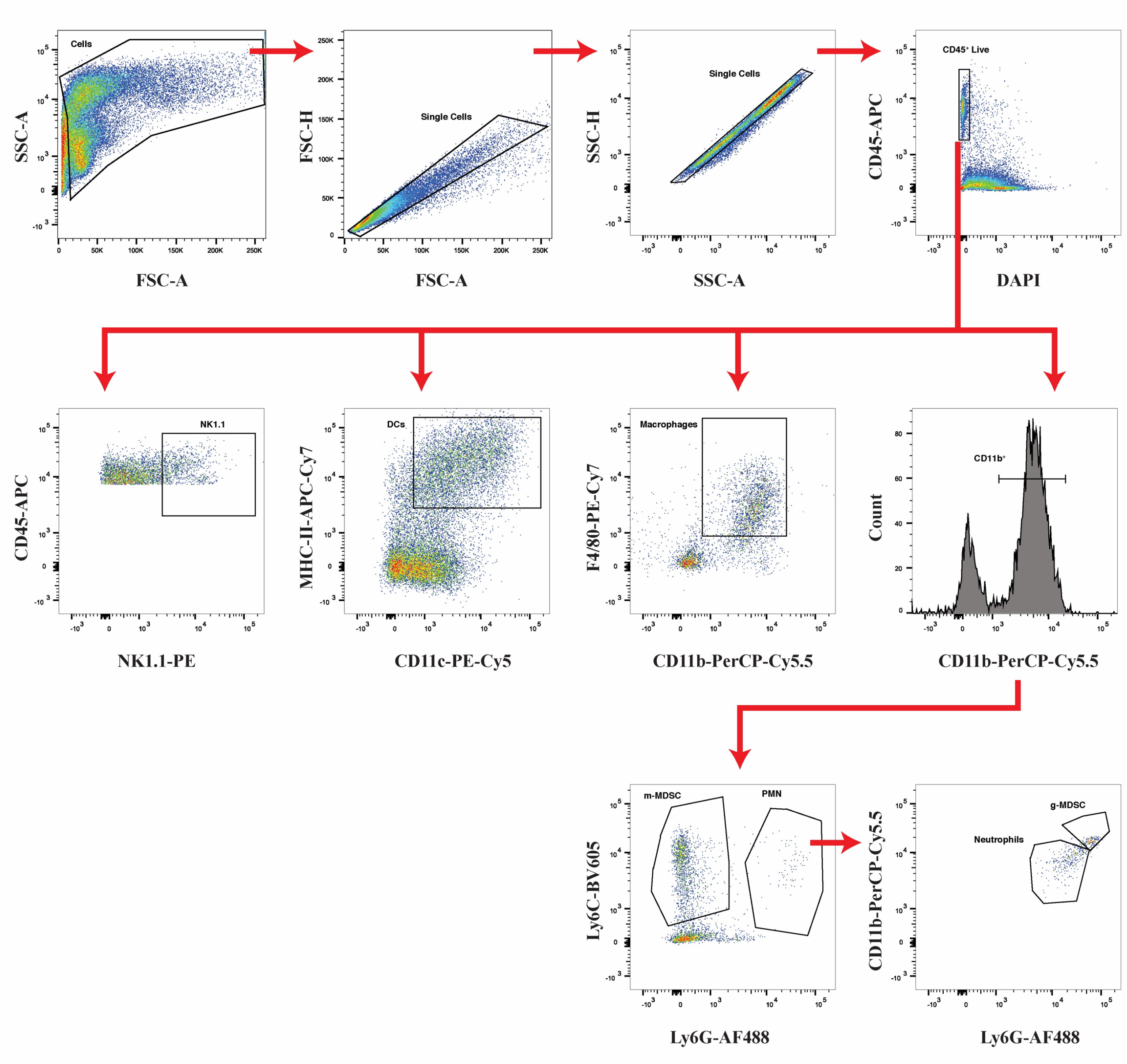

Supplementary Figure 11: Flow cytometry gating strategy for myeloid cell panel.

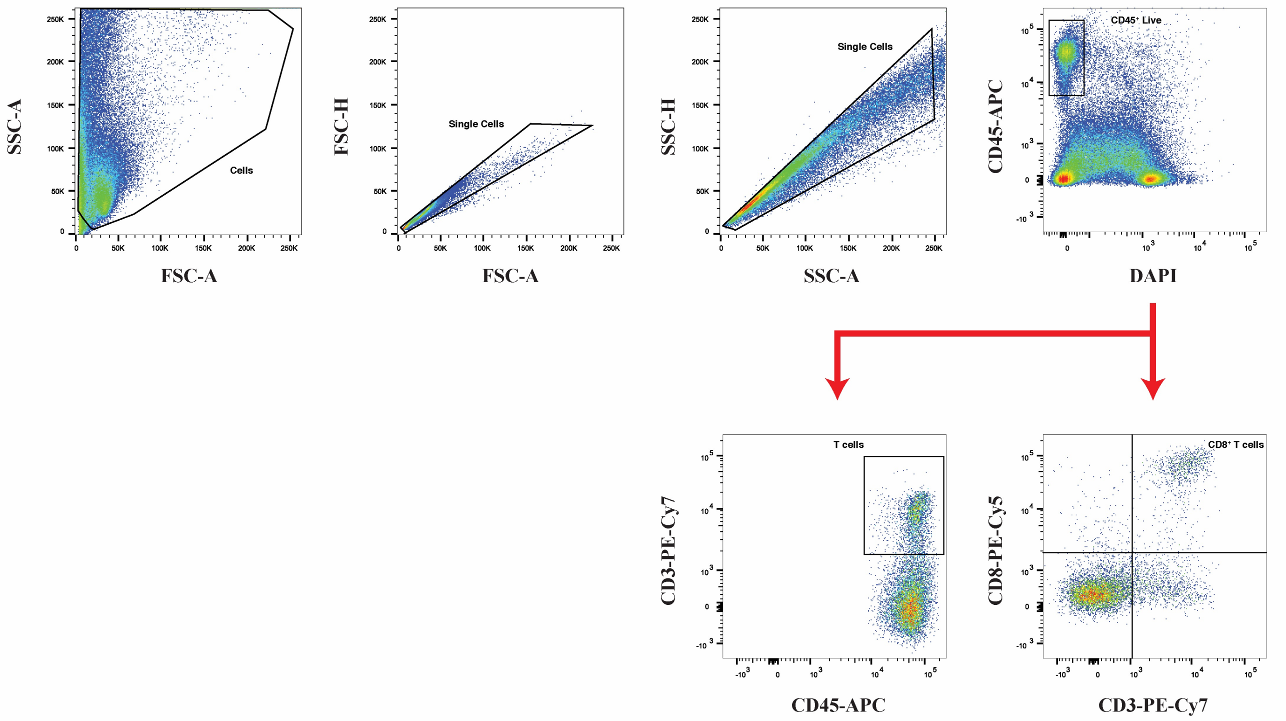

Supplementary Figure 12: Flow cytometry gating strategy for T cell panel.

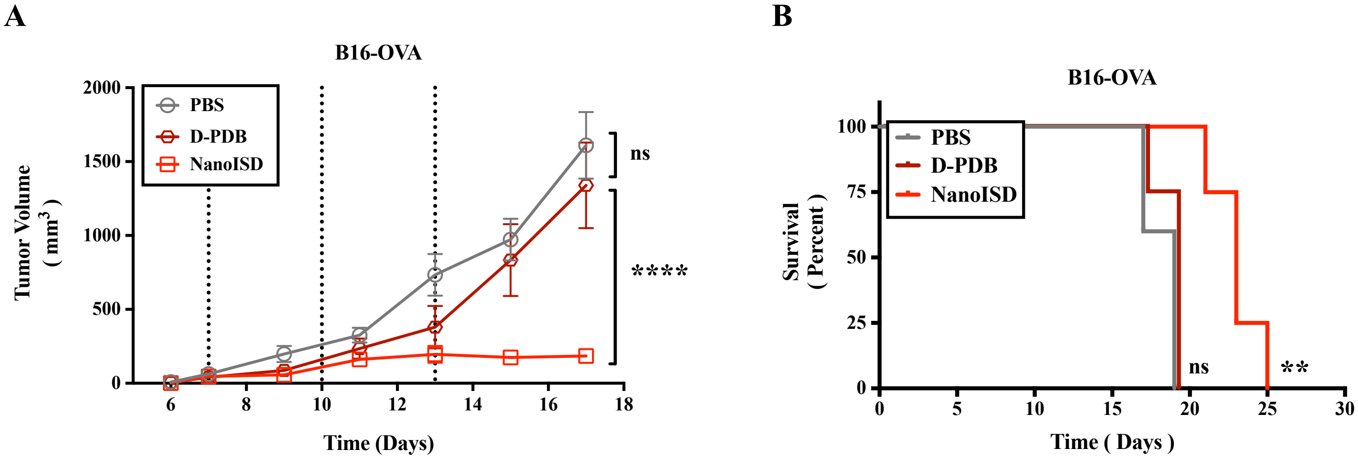

Supplementary Figure 13: Therapeutic effect of NanoISD in the B16-OVA tumor model.

(A) Tumor growth plot for B16-OVA tumors intratumorally treated with 100 µL of either PBS or NanoISD at a dose corresponding to 20 µg DNA (n = 4 or greater per treatment group). Treatments were administered 3 times q3d as indicated by the dotted lines. Tumor growth curves were truncated to the day that mice began to reach the study endpoint. A two-way ANOVA with Tukey test was used for statistical analysis. Statistics on the graph represent the analysis for the final day shown (*i.e.* day 17). **(B)** Kaplan-Meier Survival Curve for B16-OVA tumors intratumorally treated with 100 µL of either PBS, D-PDB, or NanoISD. Log rank (Mantel-Cox) test was used for statistical analysis.

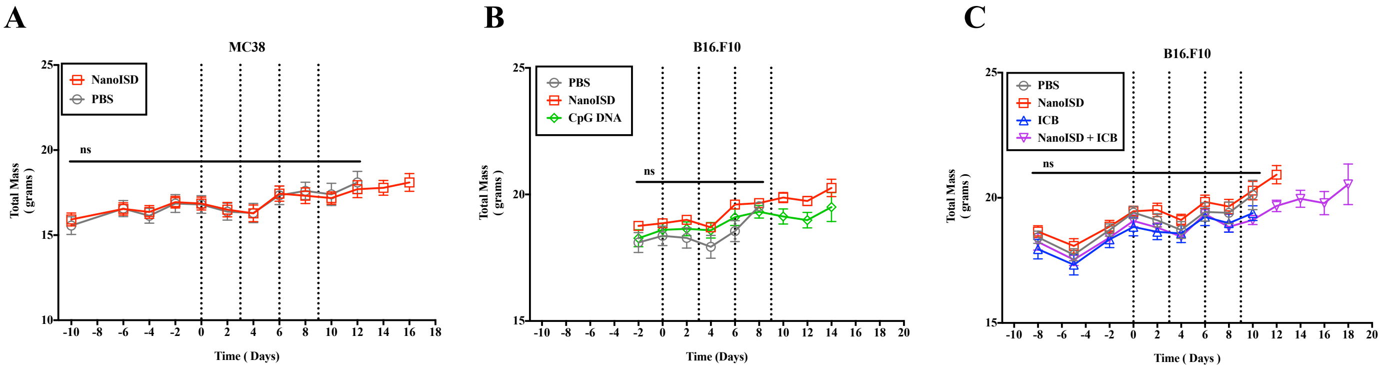

Supplementary Figure 14: Intratumorally injected NanoISD is well-tolerated.

(A) Total mouse weight over time for the mice with MC38 tumors corresponding to studies described in ***Figures 7a and 7b***. Intratumoral treatments were administered 4 times q3d as indicated by the dotted lines. A two-way ANOVA with Sidak test was used for statistical analysis. Statistics on the graph represent the analysis for each of the treatment groups relative to PBS. **(B)** Total mouse weight over time for the mice with B16-F10 tumors corresponding to studies described in ***Figures 7c and 7d***. Intratumoral treatments were administered 4 times q3d as indicated by the dotted lines. A two-way ANOVA with Tukey test was used for statistical analysis. Statistics on the graph represent the analysis for each of the treatment groups relative to PBS. **(C)** Total mouse weight over time for the mice with B16-F10 tumors corresponding to studies described in ***Figures 7e and 7f***. Intratumoral treatments were administered 4 times q3d as indicated by the dotted lines. A two-way ANOVA with Tukey test was used for statistical analysis. Statistics on the graph represent the analysis for each of the treatment groups relative to PBS.

Polymer	PAA (%)	DMAEMA (%)	BMA (%)	Molecular Weight (kDa)
1st Block	0	100	0	9.40
2nd Block	28	35	37	38.69

Supplementary Figure 15: D-PDB composition as determined by ^{1}H NMR analysis.

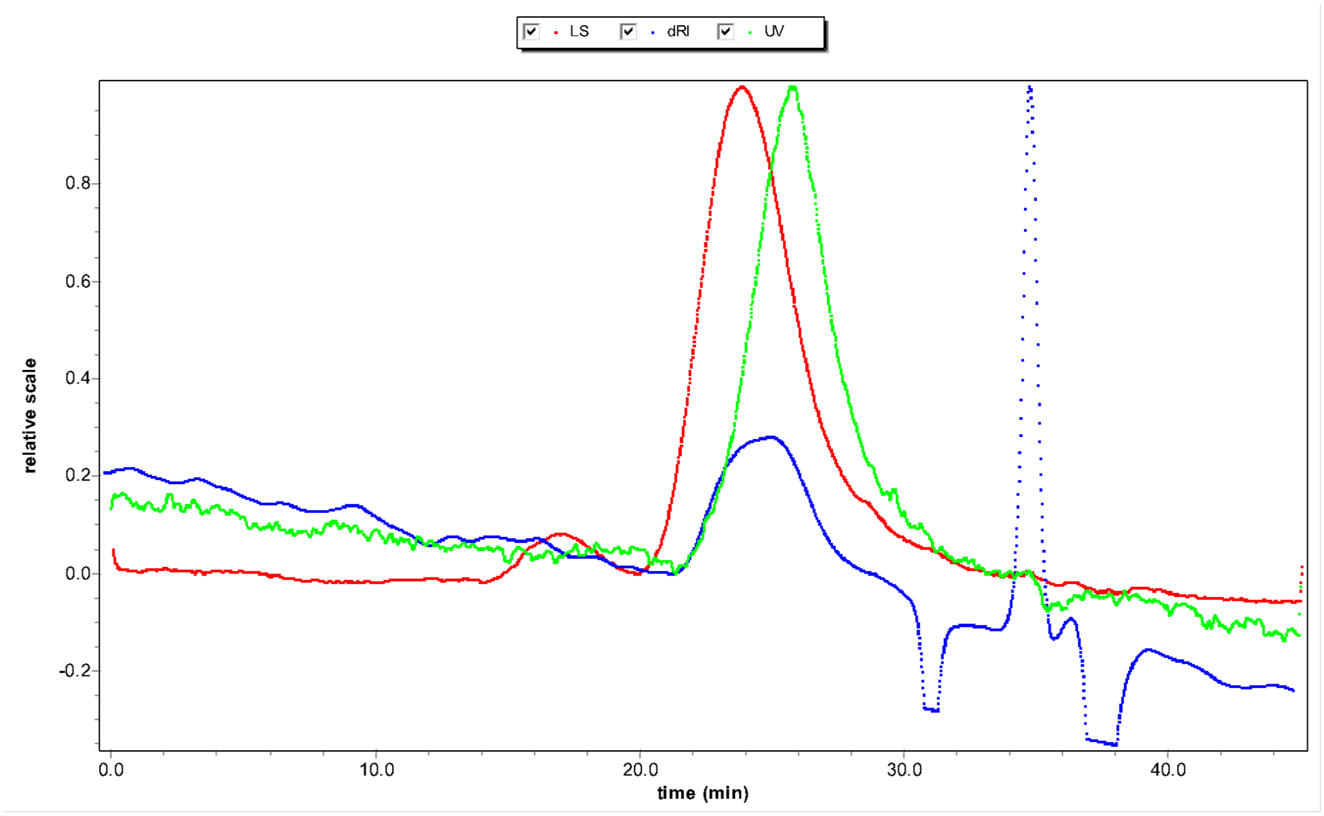

Supplementary Figure 16: GPC spectrum of D-PDB.
GPC analysis indicated that the total molecular weight of polymer was 41.56 kDa and the PDI was 1.136. Since ^{1}H NMR indicated the total molecular weight of D-PDB was 48.09 kDa, an average molecular weight of 44.8 kDa was used for all calculations regarding polymer concentration.
